# Case report: First treatment of acute ischaemic stroke in a patient on active rivaroxaban therapy using andexanet alfa and rtPA combined with early complete recovery

**DOI:** 10.3389/fneur.2023.1269651

**Published:** 2023-10-25

**Authors:** Bartosz Karaszewski, Sebastian Szczyrba, Bartosz Jabłoński, Dariusz Gąsecki, Piotr Kraszewski, Adam Wyszomirski, Robert Kowalski, Wioletta Kaliszan, Małgorzata Dąbrowska

**Affiliations:** ^1^Department of Adult Neurology, Faculty of Medicine, Medical University of Gdańsk, Gdańsk, Poland; ^2^Department of Adult Neurology, University Clinical Center in Gdańsk, Gdańsk, Poland; ^3^Clinical Research Support Center, Medical University of Gdańsk, Gdańsk, Poland; ^4^Central Pharmacy, University Clinical Center in Gdańsk, Gdańsk, Poland; ^5^Central Laboratory, University Clinical Center in Gdańsk, Gdańsk, Poland

**Keywords:** stroke, intravenous thrombolysis, anticoagulation, andexanet alfa, case report

## Abstract

Patients with non-large vessel occlusion acute ischemic stroke (NL-AIS) on oral anticoagulants (OAC) constitute the biggest portion among those who cannot receive any potential-reperfusion treatment even if they appear early in the hospital. We present the first case of therapy for NL-AIS in a patient with active anti-Xa anticoagulation, combining andexanet alfa and rtPA, who was recruited for STRoke On AntiCoagulants for Thrombolysis (acronym: STROACT), an ongoing therapeutic trial for non-LVO ischemic stroke on a DOAC. This is also the first report of the use of andexanet alfa-rtPA for AIS in a patient on rivaroxaban, which is the most frequently used non-vitamin K antagonist oral anticoagulant. The patient received the intravenous bolus of 800 mg of andexanet (contralateral arm), followed by a bolus of rtPA (10% of the calculated dose; ipsilateral arm), then a continuous infusion of andexanet at 8 mg/min for 120 min (contralateral arm), and rtPA (90% of the calculated dose; ipsilateral arm)—both stopped after completion of 38.9 and 74% of infusion dose, respectively, due to the severe adverse event related to the administration of rtPA. In this schema, both infusions are ongoing concurrently for approximately 60 min, and then andexanet is administered alone until the completion of the dose (altogether lasting approximately 3 h). The therapy was spectacularly effective, with early and complete improvement in NIHSS from 8 to 0 points in 70 min from the initiation of the therapy; mRS = 0. Obviously, a single case cannot drive any standard therapeutic decisions, but the experience we share in this article may help manage selected special clinical problems, especially when a patient's expected outcome is poor and there is no other way to help than experimentally. Additionally, it seems a valuable addition to recent meta-data on thrombolysis in anticoagulated patients.

**Trial registration:**

https://www.clinicaltrialsregister.eu. Identifier: 2020-004898-41. Date of registration: March 31, 2021.

## Introduction

More than 20% of acute ischemic strokes (AISa) are cardioembolic. The most common risk factor for blood clot formation in the heart, specifically in the left atrium, is atrial fibrillation (AF). AF, either symptomatic or asymptomatic, affects about 10–17% of the population in the age group >80 years (1), and the number is expected to increase (2). If AF is accompanied by one or more specific risk factors for AIS, oral anticoagulation (OAC) or, in special clinical situations, heparin is indicated (3, 4). However, although OACs, of which the most commonly used are rivaroxaban, dabigatran, and apixaban, reduce the risk of AIS by more than 80% (5), the risk in the latter is still significantly higher than that in the general population. Patients on an OAC with AIS due to large vessel occlusion (LVO) might be treated invasively with aspiration and/or mechanical thrombectomy, those with low OAC activity are still eligible for thrombolysis; however, most of them remain without any approved reperfusion therapeutic option as per the current clinical management high-class guidelines. STRoke on Oral AntiCoagulants for Thrombolysis (STROACT) is a multicentre, parallel-group, randomized, double-blind, placebo-controlled, non-commercial clinical trial to evaluate the efficacy and safety of reperfusion thrombolytic therapy with intravenous rtPA (alteplase) up to 4.5 h from the first symptoms of stroke (if the patient had been randomized and there was an explicit clinical justification for the delay in starting study intervention within 4.5 h window, the patient might continue in the study if rtPA (or rtPA placebo) could be administered within 6.0 h from AIS onset) in patients with non-large vessel occlusion (non-LVO) acute ischemic stroke (AIS) on a non-vitamin K antagonist anticoagulant (dabigatran, apixaban, or rivaroxaban [NOAC]) after administration of a specific antidote/reversal agent (idarucizumab for dabigatran, and andexanet alfa for apixaban and rivaroxaban) (EudraCT:2020-004898-41).

In this paper we present a case of—to our best knowledge—the first ever patient who developed AIS despite high anti-Xa activity, had no LVO, and was treated with andexanet alfa and rtPA.

## Case description

The STROACT is described elsewhere; however, for reference, the study protocol is provided in the Supplementary material. Here, we report the case of the first-ever patient treated with a four-step, i.v. andexanet-alfa and rtPA combined therapy, who was recruited to this project and had to be further unblinded.

A 61-year old, previously functionally fully independent man (pre-mRS = 0) was admitted to a primary stroke center with dysarthria, right-sided weakness (including facial asymmetry due to the central facial palsy), hemisensory loss and hemiataxia, scoring 8 on the National Institutes of Health Stroke Scale (NIHSS). The medical history of the patient included AF, hypertension, chronic heart failure, and dilated cardiomyopathy. He had an implanted cardioverter-defibrillator and, among chronically administered drugs, 20 mg of rivaroxaban daily, with the last dose taken at 9:00 a.m., approximately 40 min before stroke onset. Initial non-contrast CT with angiography showed neither acute/early tissue changes nor significant large vessel stenoses or occlusions (LVO). The patient was then transferred to our comprehensive stroke center (Department of Adult Neurology, University Clinical Center in Gdańsk) for further assessment and possible treatments, with the arrival at 12:00. Because of the high anti-Xa activity (plasma concentration of rivaroxaban was 243 ng/mL), based on the present guidelines, he was not considered for intravenous thrombolysis (IVT) (6, 7); instead, he was recruited for the STROACT study.

It is not entirely out of the question that the incident could be a transient ischemic attack (TIA) but its clinical characteristics—including severity and duration (before study treatment administration, the neurological deficit, measured using the NIHSS, did not change), and the therapy-associated recovery—are rather indicative for a stroke.

**Figure 1:**
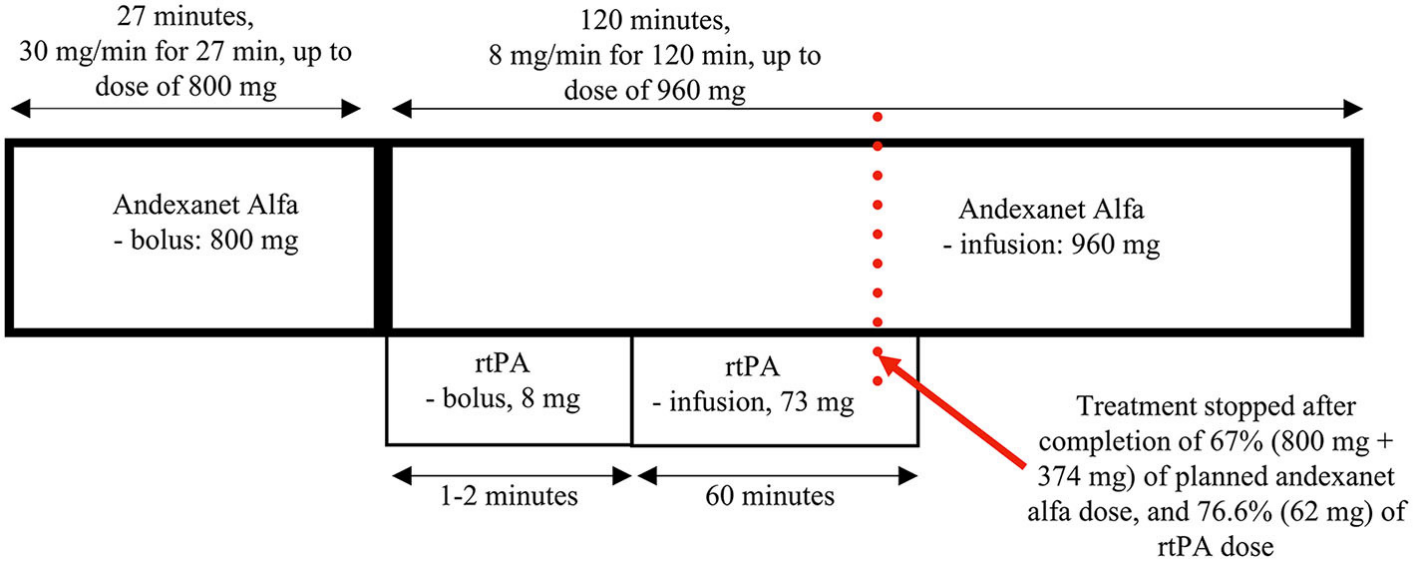
Schema of the proposed therapy with dosages as per the STROACT protocol.

The patient was recruited to the STROACT study and received sequential therapy composed of andexanet alfa and rtPA (alteplase) or (if randomized to be a “control”), placebo+placebo. In this study, high anti-Xa activity and a short time from the last intake of NOAC before the onset of stroke (<1 h) implied the selection of a high dose of antidote (andexanet alfa or placebo) (Figure 1). The patient received the antidote (or placebo) intravenous bolus of 800 mg (30 mg/min for 27 min; contralateral arm) at 2:41 p.m., followed by a bolus of rtPA (or placebo) (10% of the calculated dose; 8 mg; ipsilateral arm) at 3:12 p.m., continuous infusion of antidote (andexanet alfa or placebo) at 8 mg/min for 120 min (up to a dose of 960 mg; contralateral arm), and rtPA (or placebo) infusion (90% of the calculated dose; 73 mg; ipsilateral arm). In this therapeutic scheme, both infusions are ongoing concurrently for approximately 60 min, and then andexanet alfa (or placebo) is administered alone until completion of the dose.

Approximately 70 min after the initiation of the therapy, the patient's neurological deficit had markedly resolved with only slight remaining clumsiness of the hand; however, the NIHSS score was already 0. After another 15 min, the patient's general condition deteriorated, with hypotension (blood pressure down to 70/40 mmHg), dyspnea, flushed skin, and hives on the chest being observed, likely due to an anaphylactic reaction. The study treatment was then stopped immediately with a single intravenous dose of epinephrine (1 mg), breathing support by an oxygen mask, and parenteral fluids administered, with subsequent fast (5 min) clinical improvement. During deterioration, we did not observe recurrence of any initial neurological symptoms, and it did not result in any further complications. Nevertheless, the incidence qualified as a serious adverse event, which was assumed to be related to the administration of rtPA (also note that there was some skin redness around the area of its injection). Obviously, its occurrence revealed a result of the randomization for this subject (the risk of anaphylaxis after placebo is negligible); the patient was unblinded, the randomization outcome was confirmed (the active treatment), and proper extra information was added to the study protocol.

On discharge on the 9th day, the patient was asymptomatic, and thus the NIHSS score was still 0. Additionally, the follow up CT scan, performed 24 h after the initiation of the study treatment revealed no acute ischemic lesions (implanted cardioverter defibrillator prevented us from performing the MRI scan).

## Discussion

In this paper, we present the first case of acute non-LVO ischemic stroke in a patient receiving active anti-Xa anticoagulation, who was treated with a combination of intravenously administered andexanet alfa and rtPA. This is also the first use of andexanet alfa and rtPA to treat acute ischemic stroke in a patient on rivaroxaban, the most commonly used oral anticoagulant. In the STROACT trial, based on pharmaceutical characteristics and clinical research on the two agents, we proposed and used a special sequence of their administration that included a long bolus of andexanet, a bolus of alteplase, and simultaneous infusion of the antidote and thrombolytic (into opposite arms) for 60 min, and finally, the continued infusion of the remaining dose of the antidote, which altogether takes approximately 3 h. In the protocol, we also proposed two doses of andexanet alfa depending on the consumed dose of the anti-Xa agent and the time it was last administered (Figure 1).

In the STROACT trial, the first blood sample for determination of anti-Xa activity (corresponding to DOAC plasma concentration) is collected and processed immediately after obtaining all required forms.

Additionally, we are collecting blood in multiple time-points during and after the therapy, but any anti-Xa activities for the follow-up samples will be measured only during the first interim analysis (ie, for a major patient sample). It is worth noting that once andexanet has been administered, the testing needs some special technical lab solutions.

In the published literature, there has been only one report with andexanet alfa as part of the treatment for acute ischemic stroke; however, this was far different from the protocol proposed in STROACT, in a patient with LVO stroke (current evidence and most guidelines suggest direct endovascular therapy in these cases) and on apixaban (one of the three arms of the STROACT study also assumes recruitment of those on apixaban, although the unblinded-reported subject was on rivaroxaban) (8).

In the recent analysis, Meinel et al. (9), suggested that among selected patients with acute ischemic stroke and with recent DOAC ingestion, there was “insufficient evidence of excess harm associated with off-label IVT therapy.” However, despite a major practical value of this work including the consistent results among different selection strategies, it has important limitations regarding evidence-based medicine including its observational design and highly variable criteria to select patients that entered the analysis.

Obviously, a single case cannot drive any standard therapeutic decisions, but the experience we share in this article may help manage selected special clinical problems, especially when a patient's expected outcome is poor and there is no other way to help. The early release of these data was recommended by the Bioethical Committee in charge of the STROACT Project. Only after the results of the latter (that is, to the best of our knowledge, the only ongoing therapeutic trial in the world for ischemic stroke in anti-Xa-treated patients) are revealed, room might appear for discussing any standard use of andexanet alfa as a portion of the therapeutic schema in these subjects. This is because safety data for this combination are still poor, and it might result in various complications, including prothrombotic.

This work responds the European Stroke Organization concern that “a randomized-controlled trial on this topic is unlikely to ever be planned for financial and logistical reasons” (https://eso-stroke.org/can-we-apply-thrombolysis-for-stroke-in-patients-on-non-vitamin-k-oral-anticoagulants/). We have effectively applied for, registered in the EU, and are now recruiting patients to the clinical trial investigating the potential causative (reperfusion) therapy based on combined anticoagulant reversal and fibrinolytic agents.

The topic might be of interest also due to its epidemiological significance, since the non-LVO stroke patients on DOAC are the biggest portion among those who cannot receive any potential reperfusion therapy even if they appear in the hospital shortly after first stroke symptoms.

## Data availability statement

The original contributions presented in the study are included in the article/Supplementary material, further inquiries can be directed to the corresponding author.

## Ethics statement

The studies involving humans were approved by BioEthical Committee (Number NKBBN/75-702/2021). The studies were conducted in accordance with the local legislation and institutional requirements. The participants provided their written informed consent to participate in this study. Written informed consent was obtained from the individual(s) for the publication of any potentially identifiable images or data included in this article.

## Author contributions

BK: Conceptualization, Supervision, Writing—original draft, Writing—review and editing, Investigation. SS: Investigation, Writing—original draft, Writing—review and editing. BJ: Investigation, Writing—original draft, Writing—review and editing. DG: Investigation, Writing—original draft, Writing—review and editing. PK: Funding acquisition, Resources, Supervision, Writing—review and editing. AW: Formal analysis, Methodology, Software, Writing—review and editing. RK: Resources, Writing—review and editing. WK: Resources, Writing—review and editing. MD: Resources, Writing—review and editing.
