# Supplementary material for: Case report: First treatment of acute ischaemic stroke in a patient on active rivaroxaban therapy using andexanet alfa and rtPA combined with early complete recovery
Source: Front Neurol. 2023 Oct 25;14:1269651. doi: 10.3389/fneur.2023.1269651 (PMC10642203; doi:10.3389/fneur.2023.1269651)
Supplement: Supplementary file 1 [file Data_Sheet_1.PDF]

A multicentre, parallel group, randomised, double-blind, placebo-controlled, phase II study evaluating the efficacy and safety of reperfusion thrombolytic therapy with intravenous recombinant tissue plasminogen activator (rtPA) for ischaemic stroke in patients on the non-vitamin K antagonist oral anticoagulant after reversing anticoagulant activity with a specific antidote.

STROACT (**STR**oke on **O**ral **A**nti**C**oagulants for **T**hrombolysis)

Protocol Number: NBK241/1/2020

EudraCT Number: 2020-004898-41

Principal Investigator: Prof. Bartosz Karaszewski

Sponsor:

Medical University of Gdańsk

Marii-Skłodowskiej-Curie Street 3a, 80-210 Gdańsk

Funded by:

Medical Research Agency

Stanisława Moniuszki Street 1A, 00-014 Warszawa

Version Number: v.4.0

15 December 2022

Table of Contents

STATEMENT OF COMPLIANCE 6
NAME AND ADDRESS OF MONITOR (IF OTHER THAN SPONSOR) 6
NAME AND TITLE OF THE PERSON(S) AUTHORIZED TO SIGN THE PROTOCOL AND AMENDMENT(S) FOR THE SPONSOR 6
Key contacts: name, title, address, telephone number including Principal investigator 6
Name(s), address(es) of the clinical laborator(ies) or other medical and/or technical department(s)/institutions involved in the trial 6
Protocol signature page 7
1 PROTOCOL SUMMARY 8
1.1 Synopsis 8
1.1.1 Rationale 8
1.1.2 Overall Study Design 9
1.1.3 Objectives and Endpoints 10
1.1.4 Target Patient Population 12
1.1.5 Eligibility Criteria 12
1.1.6 Study interventions / Treatments 15
1.1.7 Study Duration 16
1.1.8 Study Sites 17
1.1.9 Sample Size 17
1.1.10 Statistical Analysis 18
1.2 Schema 18
1.3 Schedule of Activities (SoA) 21
2 INTRODUCTION 26
2.1 Current Research and Study Rationale 26
2.2 Background 27
2.3 Risk/Benefit Assessment 28
2.3.1 Known Potential Risks 28
2.3.2 Known Potential Benefits 29
2.3.3 Assessment of Potential Risks and Benefits 29
3 AIMS, OBJECTIVES AND ENDPOINTS 30
3.1 Primary Objective 30
3.2 Secondary Objectives 30
3.3 Exploratory Objectives 31
4 STUDY DESIGN 33
4.1 Overall Design 33
4.2 Blinding 34
4.3 Scientific Rationale for Study Design 34
4.4 Justification for Dose 35
4.4.1 Alteplase 35
4.4.2 Idarucizumab 35
4.4.3 Andexanet Alfa 35
4.5 End of Study Definition 36
4.5.1 Premature Study Termination 36
5 STUDY POPULATION 37
5.1 Inclusion Criteria 37
5.2 Exclusion Criteria 38
5.3 Lifestyle Considerations 39
5.4 Screen Failures 39
6 Study Procedures and Visits / PARTICIPANT RECRUITMENT PROCESS 41
6.1 Recruitment Setting 41
6.2 Screening 41
6.3 Informed Consent and Eligibility 42
6.3.1 Initial Information and Initial Approach 42

6.3.2 Consent Process ... 42
6.3.3 Consent/Assent and Other Informational Documents Provided to Participants ... 42
6.3.4 Eligibility ... 42
6.4 Randomisation ... 42
6.4.1 Timing of Randomisation ... 43
6.4.2 Treatment Allocation ... 43
6.4.3 Randomisation Process ... 43
6.4.4 Post-randomisation Actions ... 44
6.5 Study Visits And Procedures Overview ... 44
6.5.1 Visit 0 (Pre-treatment) – Screening, Enrolment and Randomisation ... 44
6.5.2 Visit 1 (Day 1) – Study Treatment Administration ... 46
6.5.3 Visit 2 (Day 2) – Post-Treatment Follow-Up 1 ... 47
6.5.4 Visit 3 (Day 7) – Post-Treatment Follow-Up 2 ... 48
6.5.5 Visit 4 (Day 30) – Post-Treatment Follow-Up 3 ... 49
6.5.6 Visit 5 (Day 90) – End of Study ... 49
6.5.7 Unscheduled Visit ... 50
7 STUDY INTERVENTION ... 51
7.1 Study Intervention(s) Admininstration ... 51
7.1.1 Study Intervention Description ... 51
7.1.2 Dosing and Administration ... 51
7.2 Trial Medicinal Product Preparation/Handling/Storage/Accountability ... 52
7.2.1 Manufactures, and Other Particulars of Medicinal Products ... 52
7.2.2 Product Storage and accountability ... 53
7.2.3 Preparation/Dispensing ... 53
7.3 Measures to Minimize Bias: Randomization and Blinding ... 53
7.3.1 Randomisation ... 53
7.3.2 Blinding 53
7.3.3 Unblinding / Unblinding procedures ... 54
7.4 Study Intervention Compliance ... 54
7.5 The Expected Duration of Subject Participation, Description of all Trial Periods Including Follow-Up, If Any 54
7.6 Concomitant Therapy ... 54
7.6.1 Post-Treatment Thromboembolism Prophilaxis ... 55
7.6.2 Rescue Medicine ... 55
8 STUDY INTERVENTION DISCONTINUATION AND PARTICIPANT DISCONTINUATION/WITHDRAWAL ... 56
8.1 Discontinuation / Interruption of Study Intervention ... 56
8.2 Participant Discontinuation/Withdrawal from the Study ... 56
8.3 Lost to Follow-Up ... 56
9 STUDY ASSESSMENTS AND PROCEDURES ... 57
9.1 Efficacy Assessments ... 57
9.1.1 National Institutes of Health Stroke Scale (NIHSS) ... 57
9.1.2 Modified Rankin Scale ... 57
9.1.3 Radiological Examinations ... 57
9.1.4 Additional Functional Assessments ... 58
9.1.5 Natriuretic Peptide ... 59
9.2 Safety Assessments ... 60
9.2.1 Events of Special Interest ... 60
9.2.2 Physical Examination ... 60
9.2.3 Vital Signs ... 61
9.2.4 Electrocardiograms ... 61
9.2.5 Clinical Safety Laboratory Assessments ... 61
9.2.6 Other Safety Assessments ... 61

9.3 Adverse Events and Serious Adverse Events ... 62
9.3.1 Detecting, Assessing and Monitoring Adverse Events AND Serious Adverse Events 62
9.3.2 Adverse Event Reporting ... 64
9.3.3 Serious Adverse Event Reporting ... 64
9.4 Unanticipated Problems ... 65
9.4.1 SARS-CoV-2 Pandemic ... 65
10 STUDY MANAGEMENT AND ADMINISTARTION ... 66
10.1 Clinical Monitoring of the Study ... 66
10.2 Clinical Data collection and Data Magement ... 66
10.3 Clinical Trial Oversight ... 66
10.3.1 Executive Committee ... 66
10.3.2 Steering Committee ... 66
10.3.3 Independent Data Monitoring Committee ... 66
10.4 Data Quality Assurance ... 67
11 STATISTICAL CONSIDERATIONS ... 68
11.1 General Considerations ... 68
11.1.1 Responsibilities ... 68
11.1.2 Statistical Analysis Plan (SAP) ... 68
11.1.3 Statistical methods ... 68
11.2 Statistical Hypotheses ... 68
11.3 Sample Size Determination and Planned Recruitment Rates ... 68
11.4 Populations for Analyses ... 69
11.4.1 Intent to Treat Population (ITT) ... 69
11.4.2 Per Protocol Population (PP) ... 69
11.4.3 Safety Analysis Population (SAF) ... 69
11.5 Statistical Analyses ... 69
11.5.1 General Assumtions ... 69
11.5.2 Efficacy Analyses ... 70
11.5.3 Other Analyses ... 72
11.5.4 Planned Interim Analyses ... 72
12 SUPPORTING DOCUMENTATION AND OPERATIONAL CONSIDERATIONS ... 74
12.1 Regulatory, Ethical, and Study Oversight Considerations ... 74
12.1.1 Case report forms (CRFs) ... 74
12.1.2 Trial Data and Documentation Held at Sites ... 74
12.1.3 Study Discontinuation and Closure ... 74
12.1.4 Confidentiality and Privacy ... 74
12.1.5 Future Use of Stored Specimens and Data ... 75
12.1.6 Key Roles and Study Governance ... 75
12.1.7 Safety Oversight ... 75
12.1.8 Clinical Monitoring ... 75
12.1.9 Protocol Deviations and Violations ... 75
12.1.10 Study Records Retention ... 76
12.1.11 Financing and Insurance ... 76
12.1.12 Publication and Data Sharing Policy ... 76
12.2 Abbreviations ... 76
13 References ... 78
14 APPENDICES ... 80
14.1 Advers Events: Definitions and Procedures for Recording, Evaluating, Follow-up, and Reporting 80
14.1.1 Definition of Adverse Events (AE) ... 80
14.1.2 Definition of Serious Adverse Events (SAE) ... 80
14.1.3 Events of Special Interest ... 81

14.1.4 Adverse Reaction (AR) ... 81
14.1.5 Suspected Unexpected Serious Adverse Reaction ... 81
14.1.6 Assessment of Adverse Events ... 81
14.2 Performance and Functional Assessment Forms ... 83
14.2.1 NIHSS Assesment Form ... 83
14.2.2 mRS Assessment Form ... 85
14.2.3 Barthel Index Assessment Form ... 87
14.2.4 MoCA Assessment Form ... 89
14.2.5 HADS Assessment Form ... 90
14.2.6 IQCODE Assessment Form ... 93

Figures and Tables

Figure 1. Study diagram. ... 18

Figure 2. Patient screening and enrolment. ... 19

STATEMENT OF COMPLIANCE

The trial will be carried out in accordance with International Conference on Harmonisation Good Clinical Practice (ICH GCP E6 (R2).

The protocol, informed consent form(s), recruitment materials, and all participant materials will be submitted to the Institutional Review Board (IRB) for review and approval. Approval of both the protocol and the consent form must be obtained before any participant is enrolled. Any amendment to the protocol will require review and approval by the IRB before the changes are implemented to the study. In addition, all changes to the consent form will be IRB-approved; a determination will be made regarding whether a new consent needs to be obtained from participants who provided consent, using a previously approved consent form.

The Principal Investigator will assure that no deviation from, or changes to the protocol will take place without prior agreement from sponsor, funding agency and documented approval from the Institutional Review Board (IRB), except where necessary to eliminate an immediate hazard(s) to the trial participants. All personnel involved in the conduct of this study have completed ICH GCP Training.

NAME AND ADDRESS OF MONITOR (IF OTHER THAN SPONSOR)

Not applicable.

NAME AND TITLE OF THE PERSON(S) AUTHORIZED TO SIGN THE PROTOCOL AND AMENDMENT(S) FOR THE SPONSOR

Sponsor: Gdański Uniwersytet Medyczny

Lead Principal Investigator

Prof. Bartosz Karaszewski
Department of Adult Neurology, University Clinical Centre, Medical University of Gdańsk, 7 Dębinki street, 80-952 Gdańsk, Poland

KEY CONTACTS: NAME, TITLE, ADDRESS, TELEPHONE NUMBER INCLUDING PRINCIPAL INVESTIGATOR

Prof. Bartosz Karaszewski
Department of Adult Neurology, Medical University of Gdańsk, University Clinical Centre, 7 Dębinki street, 80-952 Gdańsk, Poland

Phone/e-mail address: +48583492300, E-mail: bartosz@karaszewski.org

NAME(S), ADDRESS(ES) OF THE CLINICAL LABORATOR(IES) OR OTHER MEDICAL AND/OR TECHNICAL DEPARTMENT(S)/INSTITUTIONS INVOLVED IN THE TRIAL

Clinical Research Organisations (CRO):

50BIO COM Sp. z.o.o., Złota 59 Street, 00-120 Warsaw, Poland

BioStat Sp. z.o.o., Kowalczyka 17 Street, 44-206 Rybnik, Poland

PROTOCOL SIGNATURE PAGE

I have read and approved this protocol. My signature, in conjunction with the signature of the Investigator, confirms the agreement of both parties that the medical experiment will be conducted in accordance with the protocol and all applicable laws and regulations including, but not limited to, the International Conference on Harmonisation (ICH) Guideline for Good Clinical Practice (GCP), the EU Directive number 1924/2006, Polish Act of Food and Nutrition Safety dated 25 Aug 2006 [amended text], Polish Act on the Medical Profession dated 5 Dec 1996 [amended text], the ethical principles that have their origins in the Declaration of Helsinki, and applicable privacy laws.

Nothing in this document is intended to limit the authority of any Investigator participating in this clinical research to provide emergency medical care under applicable regulations.

**Lead Principal Investigator (Coordinating site)**		**Sponsor**	
Prof. Bartosz Karaszewski		Gdański Uniwersytet Medyczny	
B. Karaszewski	DEC 20, 2022	Signature Not Verified<br>Dokument podpisany przez Marcin Gruchała; GUMed<br>Data: 2022.12.22 10:40:17 CET	
Signature:	Date:	Signature:	Date:

Participating site signatures

I have read the attached protocol entitled "A multicentre, parallel group, randomised, double-blind, placebo-controlled, phase II study evaluating the efficacy and safety of reperfusion thrombolytic therapy with intravenous recombinant tissue plasminogen activator (rtPA) for ischaemic stroke in patients on the non-vitamin K antagonist oral anticoagulant after reversing anticoagulant activity with a specific antidote." and agree to abide by all provisions set forth therein.

I agree to comply with all applicable laws and regulations as declared in the clinical trial protocol.

I agree to ensure that the confidential information contained in this document will not be used for any other purpose other than the evaluation or conduct of the clinical investigation without the prior written consent of the Sponsor (Medical University of Gdańsk).

Principal Investigator (Participating site)

Name:

Signature: Date:

1 PROTOCOL SUMMARY

1.1 SYNOPSIS

Tittle of the Study

A multicentre, parallel group, randomised, double-blind, placebo-controlled, phase II study evaluating the efficacy and safety of reperfusion thrombolytic therapy with intravenous recombinant tissue plasminogen activator (rtPA) for ischaemic stroke in patients on the non-vitamin K antagonist oral anticoagulant after reversing anticoagulant activity with a specific antidote.

Short title: Reperfusion thrombolytic therapy for ischaemic stroke in patients on the non-vitamin K antagonist oral anticoagulants.

Acronym: **STROACT** (**STR**oke on **O**ral **A**nti**C**oagulants for **T**hrombolysis)

1.1.1 RATIONALE

Stroke is the most common cause of permanent complex disability in adults and one of the most common causes of death worldwide. Around 85% of all strokes are related to an acute focal cerebral ischemia (acute ischaemic stroke, AIS) that might be caused by acute occlusion or critical stenosis of one or more intracranial or extracranial arteries. More than 20% of AIS are cardioembolic, that is a condition in which blood clots located in the left atrium (or the left ventricle) under specific hemodynamic circumstances are pumped with the blood flow towards the brain circulation and occlude one or multiple arteries (extracranial, e.g. internal carotid artery or, more often, the intracranial – usually middle cerebral artery).

The most common risk factor for blood clot formation in the left atrium (or the left ventricle) is atrial fibrillation (AF) if combined with other selected conditions. Estimates of the Polish Neurological Society and the Polish Cardiac Society, as well as preliminary reports from researchers of the NOMED-AF project, suggest that AF (symptomatic and non-symptomatic) affects >23% of the population in the age group > 65 years. If AF is accompanied by one of the other specific risk factors of AIS, it is an indication for oral anticoagulation (OAC), and much less commonly for subcutaneous administration of heparin (CHADS2VASc classification).

The benefit-risk evaluation of OAC administration (reduction of AIS risk vs. increased risk of clinically relevant bleeding) in such defined patient population is clearly positive. However, although OAC therapy (most commonly used are the so-called new OAC [NOAC], i.e., rivaroxaban, dabigatran or apixaban, and rarely others) reduces the risk of AIS by more than 80%, and it still higher than in the general population.

The incidence of AIS in patients using OAC therapy in Poland has been roughly estimated at 8,000 annually, which includes approximately 5,000 patients using NOAC. Providing that other criteria are met, some of these patients might be treated invasively with aspiration and/or mechanical thrombectomy, some others will be still eligible for thrombolysis (low OAC activity), but most remain without any approved reperfusion therapeutic possibility as per current clinical management guidelines.

The aim of this study is to develop and assess the first causative (reperfusion) therapy for this group of patients based on combined anticoagulant reversal and fibrinolytic agents. STROACT clinical trial is a multicentre, parallel groups, randomised, double blind, placebo-controlled study evaluating efficacy and safety of a new intervention consisting of sequential intravenous administration of NOAC-specific antidote and thrombolysis with rtPA.

1.1.2 OVERALL STUDY DESIGN

STROACT is a multicentre, parallel-group, randomized, double-blind, placebo-controlled, non-commercial clinical trial to evaluate the efficacy and safety of reperfusion thrombolytic therapy with intravenous rtPA (alteplase) in patients with acute ischaemic stroke (AIS) on the non-vitamin K antagonist anticoagulant (NOAC: dabigatran, apixaban, or rivaroxaban) after administration of a specific antidote/reversal agent (idarucizumab for dabigatran, and andexanet alfa for apixaban and rivaroxaban). Outline of the study design and patient flow are presented in Section 1.2 Figure 1. Study diagram and Figure 2. Patient screening and enrolment.

Patients admitted to the neurological emergency unit with symptoms of acute ischaemic stroke (AIS), with no previous significant chronic disability (0-2 points on the modified Rankin Scale [mRS]), and on NOAC treatment due to any reason but mostly as a prevention of cardioembolic events, will undergo standard diagnostic procedures and may be considered for recruitment into the STROACT study. Informed consent for participation in the study (signature of the informed consent form [ICF]) must be obtained as soon as possible when the patient is considered as a candidate for the study.

After signing ICF and preliminary confirmation of eligibility for the study (see Section 1.1.5) patients will be allocated to one of the 3 parallel study groups based on currently used NOAC (see Section 1.2 Fig. 1) as follows: (A) – patients who were receiving dabigatran before the AIS ("patients on dabigatran"), (B) – patients on apixaban, and (C) patients on rivaroxaban.

All patients MUST have a pre-enrolment brain CT or MRI to exclude intracranial haemorrhage and some stroke "mimics" (see Section 1.1.5 and 1.3). In addition, all patients with $\geq$ 6 points in National Institutes of Health Stroke Scale (NIHSS) and others with symptoms that might suggest acute occlusion of a large artery (based on investigator's individual decisions), will undergo CT or MRI angiography of cerebral arteries to confirm or exclude large vessel occlusion (LVO); patients with LVO may be qualified for primary mechanical thrombectomy (MT) and will not be included into the study.

A blood sample will be obtained from each patient to determine anti- IIa/Xa activity (which is assumed to be directly proportional to the plasma concentration of NOAC) or blood concentration of dabigatran itself in the respective arm of the study. Patients with anticoagulant activity >50 ng/ml or the respective concentration of dabigatran (in the respective arm of the study) qualify to the investigated treatment, i.e., to NOAC-specific reversal agent administration followed by intravenous rtPA; patients with anticoagulant activity level $\leq$50 ng/ml (or with the respective concentration of dabigatran in the respective arm of the study) may qualify to receive thrombolysis on regular basis and eventually will not be considered for the STROACT study. However, in those on dabigatran, it is possible to enrol a patient into the study based solely on a patient's or witness' report on the last intake of the anticoagulant ie, when it was administered within the last 24 hours preceding initiation of the study treatment. In these cases it is still obligatory to collect (and then store) blood for (post-hoc) anti-IIa testing. The procedure is described in the Laboratory Manual.

In this study we do not use other laboratory tests that might mirror anticoagulant activity to guide enrolment of patients.

Eligible patients allocated to each study group will be randomised at 1:1 ratio to receive experimental treatment or corresponding placebo (see also Fig. 1 in Section 1.2). Study treatment is composed of sequential intravenous administration of NOAC-specific antidote (or placeboes) and the infusion of rtPA or corresponding placebo.

Standard diagnostic procedures, allocation to the study group and randomisation should be completed within 3.0 hrs from AIS onset to minimise the risk of delay to start the study treatment (thrombolysis with rtPA) in the optimal time window up to 4.5h, but no later than 6h from the AIS onset.

Randomisation scheme will include severity of the AIS at randomisation (<6 points in NIHSS, and ≥ 6 points in NIHSS) as a stratification factor in all 3 study groups (arms).

After receiving study treatment patients will be assessed at 24 (+/-4) hrs (Visit 2), 7 (+/-1) days (Visit 3), 30 (+/-2) days (Visit 4), and 90 (+/-3) days after rtPA administration and collected data will be recorded in the eCRF (please see Schedule of Assessments in section 1.3).

Prophylaxis of venous thromboembolism (VTE) and new cardioembolic events should be initiated and follow local standard of care and must be documented in patient's medical records and eCRF.

An Independent Data Monitoring Committee (IDMC) will be established before the start of patients' enrolment to monitor progress of the clinical study and to ensure patients' safety. This will include reviewing unblinded safety and the efficacy data and estimating the benefit-risk balance during the study and overseeing the overall conduct of the study including protocol compliance.

The composition and responsibilities of the IDMC will be defined in detail in the IDMC charter.

1.1.3 OBJECTIVES AND ENDPOINTS

This study aims to evaluate the efficacy and safety of intravenous thrombolysis with rtPA in patients with acute ischemic stroke on chronic NOAC treatment after neutralisation of the anticoagulant activity by administration of a specific reversal agent (antidote).

1.1.3.1 PRIMARY OBJECTIVE

**Objective**	**Outcome measure / Endpoint**
**Efficacy**	
To assess the efficacy of the investigational therapy in patients with acute ischaemic stroke on the non-vitamin K antagonist oral anticoagulants compared to placebo using modified Rankin Scale (mRS).	Proportion of patients with excellent and good functional outcome assessed with modified Rankin scale (mRS) (mRS 0-1 and 0-2, respectively) at 90 days (+/- 3 days) after study treatment administration.

1.1.3.2 SECONDARY OBJECTIVES

**Objectives**	**Outcome measures / Endpoints**
**Efficacy**	
To assess the efficacy of the investigational therapy in patients with acute ischaemic stroke on the non-vitamin K antagonist oral anticoagulants with pre-stroke functional status of 0-1 points in mRS compared to placebo using modified Rankin Scale (mRS).	Proportion of patients with excellent functional status assessed with modified Rankin scale (mRS) (mRS 0-1) at 90 days (+/- 3 days) after investigational treatment administration.

To assess the efficacy of the investigational therapy in patients with acute ischaemic stroke on the non-vitamin K antagonist oral anticoagulants compared to placebo using NIHSS score.	Change in NIHSS score from baseline assessed at 7 (+/-1 day) and 90 days (+/- 3 days) after investigational treatment administration.
**Safety**	
To assess the incidence of fatal events after the investigational therapy in patients with acute ischaemic stroke on the non-vitamin K antagonist oral anticoagulants compared to placebo.	Incidence of deaths:<br>- Deaths from any cause<br>- Deaths subdivided by cause<br>at 7 (+/-1 day), 30 (+/-2 days) and 90 days (+/ 3 days) after investigational treatment administration.
To assess the incidence of non-fatal events after the investigational therapy in patients with acute ischaemic stroke on the non-vitamin K antagonist oral anticoagulants compared to placebo.	Incidence of non-fatal events defined as<br>- Recurrent ischaemic stroke<br>- Haemorrhagic stroke (ICH or SAH)<br>- Neurological deterioration (NIHSS) at 7 (+/-1 day) and 90 days (+/- 3 days) after investigational treatment administration.
To evaluate safety of the investigational therapy in patients with acute ischaemic stroke on the non-vitamin K antagonist oral anticoagulants compared to placebo assessed with neuroimaging.	Rate and severity of early (symptomatic and asymptomatic) intracranial haemorrhage detected by neuroimaging:<br>- CT or MRI at 24hrs (+/- 4hrs) after investigational treatment administration<br>- CT or MRI at 7 days (+/- 1 day) after investigational treatment infusion<br>and assessed according to European Cooperative Acute Stroke Study (ECASS II) classification.<br>Note: Symptomatic Intracranial Haemorrhage (sICH) defined as deterioration of stroke severity in NIHSS of ≥ 4 points with parenchymal haemorrhage type 2 in neuroimaging.
To assess characteristics of major clinically significant extracranial bleedings after the investigational therapy in patients with acute ischaemic stroke on the non-vitamin K antagonist oral anticoagulants compared to placebo.	Incidence and severity of major extracranial haemorrhages defined as:<br>- fatal<br>- severe enough to require transfusion or surgery<br>- an absolute decrease in haemoglobin > 5 g/dL<br>- a decrease in haematocrit of > 15%<br>- bleeding in persistent or temporary serious disability)
To assess overall safety profile of the investigational therapy in patients with acute ischaemic stroke on the non-vitamin K antagonist oral anticoagulants compared to placebo.	Incidence and category of AEs reported during the study.

1.1.3.3 EXPLORATORY OBJECTIVES

Objective	Outcome measure / Endpoint

To assess BNP or NT-proBNP plasma levels after the investigational therapy in patients with acute ischaemic stroke on the non-vitamin K antagonist oral anticoagulants after administration of a specific reversal agent compared to placebo.	Change in BNP or NT-proBNP plasma concentration at Day 7 compared to pre-treatment levels.
To assess functional independence after the investigational therapy in patients with acute ischemic stroke on the non-vitamin K antagonist oral anticoagulants compared to placebo using Barthel Index scale.	Change in Barthel Index score measured at Day 90 compared to Day 7.
To assess cognitive function after the investigational therapy in patients with acute ischemic stroke on the non-vitamin K antagonist oral anticoagulants compared to placebo using MoCA scale.	Change on MoCA scale measured at Day 90 compared to Day 7.
To assess pre-stroke cognitive function after the investigational therapy in patients with acute ischemic stroke on the non-vitamin K antagonist oral anticoagulant compared to placebo using IQCODE scale.	Assessment of pre-stroke cognitive impairment measured by IQCODE scale at Day 7.
To assess “mental” condition (i.e. depression and anxiety) after the investigational therapy in patients with acute ischemic stroke on the non-vitamin K antagonist oral anticoagulants compared to placebo using HADS scale.	Change on HADS scale measured at Day 90 compared to Day 7.
To assess duration of the hospitalisation after the investigational therapy in patients with acute ischemic stroke on the non-vitamin K antagonist oral anticoagulants compared to placebo.	Number of days from the hospital admission to discharge.

1.1.4 TARGET PATIENT POPULATION

Previously functionally independent patients (0-2 points on the modified Rankin performance scale) taking one of the oral non-vitamin K antagonist oral anticoagulants (rivaroxaban, apixaban or dabigatran) due to any reason (most commonly as a prevention of stroke and systemic embolism in patients with nonvalvular atrial fibrillation) who develop an ischemic stroke, in the absence of other possibility of specific (reperfusion) AIS treatment (which in this case is equal to “beyond the medical criteria for mechanical thrombectomy”).

1.1.5 ELIGIBILITY CRITERIA

1.1.5.1 INCLUSION CRITERIA

To be eligible for enrolment patient **must meet all** the following inclusion criteria:

1. Obtaining informed consent to participate in the trial prior to randomisation.

   NOTE: Patients whose neurological deficit is severe enough to make it impossible to sign the consent form are allowed to give only their oral consent to participate in the study. However, this

consent should be additionally certified by the signature of two independent witnesses (who are neither family members of the patient nor the STROACT study staff) or by the signature of his/her legal representative. **Patients with aphasia and/or other speech disorders may be included into the study if following neurological assessment of the recruiting stroke physician, they are able to understand all important information about the study.**

2. Age ≥18 years.
3. Clinical diagnosis of acute ischemic stroke (sharply defined onset of first symptoms) resulting in a disabling neurological deficit.
4. Therapy with an oral anticoagulant that is the non-vitamin K antagonist oral anticoagulant (dabigatran, apixaban, or rivaroxaban) with laboratory confirmed therapeutic anti-IIa/anti-Xa activity measured as a plasma concentration > 50 ng/mL or the corresponding blood concentration of dabigatran (in the respective arm of the study). However, in patients on dabigatran, it is possible to enrol such a subject into the study based solely on a patient's or witness' report on the last intake of the anticoagulant ie, when it was administered within the last 24 hours preceding initiation of the study treatment. In these cases it is obligatory to collect and store blood for post-hoc anti-IIa testing.
5. Administration of study intervention (intravenous thrombolysis with alteplase or placebo) should be possible to start within 4.5 hours from AIS symptoms onset or the last time the patient was seen without symptoms, as per investigator's judgment.

   NOTE: If patient had been randomised and there was an explicit clinical justification for delay in staring study intervention within 4.5h window, patient might continue in the study if rtPA (or rtPA placebo) could be administered within 6.0h from AIS onset.

   NOTE: In patients recruited to STROACT study, in addition to the inclusion / exclusion criteria, apply all standard clinical practice indications and contraindications for rtPA administration in acute ischemic stroke unless stated otherwise in this protocol.

1.1.5.2 EXCLUSION CRITERIA

To be eligible for enrolment patient **must not meet** any of the following exclusion criteria:

1. Occlusion of a large intracranial vessel in CT/MR angiography (CTA/MRA), corresponding to the current acute neurological deficit being an indication for primary mechanical thrombectomy.

   NOTE 1: Patients who qualified to the mechanical thrombectomy cannot be enrolled to the STROACT study.
2. Significant disability prior to the current stroke event defined as >2 points on the modified Rankin Scale (mRS) and/or significant impairment of the cognitive function prior to AIS (the latter documented in patient's medical records).
3. Mild and rapidly improving neurological deficit with high probability of complete recovery.
4. Clinically severe stroke with >18 points in NIHSS.
5. Neuroimaging findings that might be responsible for acute neurological deficit ("stroke mimics") and/or are contraindications for standard thrombolytic treatment: such as intracranial and/or intracerebral bleeding, tumours, abscesses and other.
6. Treatment with the following anticoagulants:
   a. Oral vitamin K antagonist (warfarin, acenocumarol),
   b. Unfractionated heparin,
   c. Low molecular weight heparin, or
   d. Inhibitors of coagulation factor Xa or IIa other than dabigatran, rivaroxaban, or apixaban

7. Whole blood, and/or blood clotting factors (such as: prothrombin complex concentrate [PCC], recombinant factor VIIa [rVIIa], fresh frozen plasma [FFP]) administered within 7 days before study treatment initiation.
8. Anti-IIa/ Xa activity (which is assumed to be directly proportional to the NOAC plasma concentration) is ≤50 ng/mL or the respective concentration of dabigatran (in the respective arm of the study).
9. CT or MRI initial lesion volume >1/2 of the anatomical perfusion area of the middle cerebral artery (MCA), or anterior cerebral artery (ACA), or posterior cerebral artery (PCA).
10. Suspected subarachnoid haemorrhage based on specific symptomatology and/or physical examination (even if CT/MRI is normal).
11. Any history of subarachnoid or intracerebral haemorrhage, so not including previous (currently normal in neuroimaging) traumatic sub-or epidural hematomas > 6 months before the current acute stroke.
12. Any past (chronic) medical illnesses that significantly impairs patient's functional status down to mRS 3 points or more (thus not only related to CNS pathologies and including cognitive impairment), and/or with a poor prognosis (e.g., neoplasms individually assessed to be of poor prognosis).
    NOTE: Patients after treatment of intracranial aneurysm may be considered for recruitment into the STROACT trial if the procedure was performed > 3 months prior to randomisation.
13. History of major surgery / trauma within 2 months before the current acute stroke.
14. History of acute ischemic stroke or any other medical condition treated with intravenous thrombolysis, or ischemic stroke treated with mechanical thrombectomy, within the 72 hours preceding the current patient's stroke symptoms.
15. Recent (within 10 preceding days) traumatic external heart massage, obstetrical delivery, lumbar puncture, any puncture of a non-compressible blood vessel.
16. Recent (within 4 preceding weeks) myocardial infarction.
17. Severe trauma at the onset of acute ischemic stroke (e.g., skull fracture, long bone fracture, pelvic fracture).
18. Expected need for major surgery within 72 hours after randomisation (e.g., laparotomy, hip femoral/pelvic fracture surgery, endarterectomy).
19. Cerebral venous sinus thrombosis (CVST).
20. Pulmonary embolism.
21. Suspected infective endocarditis and/or pericarditis.
22. Acute pancreatitis.
23. Systemic or suspected cerebral vasculitis.
24. Documented active ulcerative gastrointestinal disease during the last 3 months, documented oesophageal varices.
25. Neoplasm with increased bleeding risk.
26. Severe liver disease including acute hepatic failure, cirrhosis with/or without portal hypertension.
27. Haemorrhagic retinopathy.
28. Haemorrhagic diathesis (e.g., von Willebrand disease, haemophilia, and similar inherited coagulopathies).
29. Platelet count <100,000/mm^3.
30. Active or recent severe, life-threatening bleeding.
31. Congenital or acquired coagulopathy presenting with:
    a. Prolonged aPTT above 30% of the upper limit of normal (local laboratory reference range),
    b. Increased INR ≥1.7
32. Blood glucose <50 mg/dl (2.8 mmol/l) or >400 mg/dl (22.2 mmol/l)

NOTE: Such patients may be enrolled into the STROACT trial if CT or MRI lesion corresponds to persisting acute neurological deficit even after restoration of blood glucose down to 250 mg/dl or less.

33. Severe high blood pressure, i.e., systolic blood pressure (SBP) > 185 mmHg or diastolic blood pressure (DBP) > 110 mmHg immediately before the study treatment administration or suspected need for aggressive medication use (e.g., labetalol, urapidil) to maintain blood pressure below these values during further reversal and/or thrombolytic treatment.
34. Pregnancy.
35. Predicted life expectancy <3 months.
36. Participation in another clinical trial at the time of randomisation or planned inclusion in another clinical trial within less than 90 days of randomisation, provided that protocols of these trials interfere pathophysiologicaly or formally and administratively with the STROACT study.
37. Previous participation in the current clinical trial.
38. Advanced renal failure (eGFR <30 mL/min/1.73m^2).
39. Active infection with SARS-CoV-2 (up to 10 days from the first positive testing with any recommended assay or from the first symptoms of infection or severe “long” COVID-19 / severe Post-COVID Neurological Syndrome).

1.1.6 STUDY INTERVENTIONS / TREATMENTS

Patients with AIS on NOAC (dabigatran, apixaban or rivaroxaban) who are eligible for the study will receive reperfusion thrombolytic treatment with intravenous rtPA (alteplase) following administration of the specific reversal agent / antidote (andexanet alfa for apixaban or rivaroxaban; idarucizumab for dabigatran).

The study intervention is defined as sequential therapy including administration of NOAC specific neutralising (reversal) agent / antidote (idarucizumab or andexanet) and rtPA (alteplase) or corresponding placebo.

Active treatment and placebos for antidote and rtPA (0.9% NaCl solution) will be prepared by the unblinded person only after confirmation by the investigator of the final intention to treat the randomised patient, after receipt of laboratory results of the anti-IIA or anti-Xa (must be >50 ng/mL) or dabigatran concentration. However, in patients on dabigatran, it is possible to enroll such a subject into the study based solely on a patient’s or witness’ report on the last intake of the anticoagulant ie, when it was administered within the last 24 hours preceding initiation of the study treatment. In these situations it is obligatory to collect and store blood for post-hoc anti-IIa testing. The procedure is described in the Laboratory Manual. People responsible for the preparation of investigational medicinal products will maintain blinding of the clinical study personnel to the treatment allocation (after randomisation of the patient in the eCRF the unblinded person will receive notification with treatment allocation). The tested treatments and/or placeboes will be prepared in places indicated by in-hospital PIs taking into account all local organizational specificities.

All components of the study intervention (combination / sequential therapy) are commercially available medicinal products. Dosage and administration will follow the current local prescribing information. Study groups with respective investigational treatment are outlined below.

Study treatment group	Antidote and rtPA dosing and administration
Study group A<br>(patients on dabigatran)	1. Antidote<br>Idarucizumab at a dose of 5 g (2 vials of 2.5 g/50 mL) will be administered intravenously as two consecutive infusions or over 5 to 10 minutes each, or as a rapid intravenous injection (bolus).<br>2. Reperfusion thrombolytic treatment<br>Alteplase at a dose of 0.9 mg/kg of body weight (maximum 90 mg) administered intravenously; 10% of the calculated dose in a rapid intravenous injection (bolus) within 1-2 minutes, and 90% of the calculated dose in an intravenous infusion over 1 hour (syringe pump).
Study group B<br>(patients on apixaban)	1. Antidote<br>Andexanet alpha will be administered as an intravenous bolus a:<br>• Low dose: target infusion rate of 30 mg/min for approximately 14 minutes (up to the dose of 400 mg), followed by a continuous infusion at 4 mg/min for 120 min<br>• High dose: target infusion rate of 30 mg/min for approximately 27 minutes (up to the dose of 800 mg), followed by a continuous infusion at 8 mg/min for 120 min<br>(administration in a syringe pump after reconstitution of all required vials).<br>2. Reperfusion thrombolytic treatment<br>Alteplase (rtPA) - the same dosing and administration instructions as in study group A.
Study group C<br>(patients on rivaroxaban)	1. Antidote<br>Andexanet alpha - the same dosing and administration instructions as for the study group B.<br>2. Reperfusion thrombolytic treatment<br>Alteplase (rtPA) - the same dosing and administration instructions as for the study group A, and B.

Dosing of andexanet alfa in the STROACT study (low dose or high dose) will be guided by anti-Xa activity (for either apixaban or rivaroxaban) and the time from the last intake of apixaban or rivaroxaban dose before AIS (please refer to the table below).

Anti-Xa activity	Time form the last dose of apixaban or rivaroxaban		
	**<4h or unknown**	**4-8h**	**≥8h**
>50ng/mL, <100ng/mL	High dose	Low dose	Low dose
≥100ng/mL	High dose	High dose	High dose

1.1.7 STUDY DURATION

Clinical trial timelines
First patient to be enrolled: 4Q2021
Last patients to be enrolled: 1Q2025
End of study (last patient visit): 2Q2025

The enrolment of new patients will be terminated when the minimum number of patients assumed in the sample size analysis for the primary endpoint will be achieved.

Study duration for participant

The expected duration of the study for participants is up to 90 (+/-3) days from enrolment the STROACT clinical trial (receiving rtPA bolus).

1.1.8 STUDY SITES

All clinical centres participating in STROACT study should have effective Stroke Unit and trained personnel to provide high quality, standardised specialty care for patients with acute stroke. It includes, but is not limited to:

- Written protocol/procedures for expedite diagnosis and treatment of patients with suspected acute stroke
- Immediate access to CT or MR brain scanning (24 hours a day)
- Specialised facility where thrombolysis may be administered, and the patient can be monitored according to study protocol.

There are several clinical centres in Poland which will participate in this clinical trial. The number of participating sites may change during the study if deemed appropriate by the Steering Committee.

1.1.9 SAMPLE SIZE

It is expected that approximately 300 patients will be enrolled/randomised in the study with approximately 100 patients per each patients' study group.

Group A (dabigatran): n=100

Group B (apixaban): n=100

Group C (rivaroxaban): n=100

1.1.9.1 SAMPLE SIZE CALCULATION

Due to nature of this study (proof of concept phase II trial), with effect size being unknown, following assumptions were made:

- Proportion of subjects randomized to receive IMP achieving primary endpoint will be 40% and this proportion will be identical in a placebo group,
- A non-inferiority margin d will be equal to 15%
- One sided significance level 0.2
- Power of 70%
- One interim analysis (with Hwang-Shih-DeCani α pending function)
- A drop-out rate of 10%.

Taking these into consideration, a minimal number of subjects per group is 46 – so 92 subjects in each study arm.

As no comparisons between study arms are planned, no multiplicity correction beyond that resulting from an interim analysis are planned.

Sample size calculations were made in R version 3.6 using gsDesign library version 3.1.1.

1.1.10 STATISTICAL ANALYSIS

Detailed description of study populations / subgroups, including rules for inclusion and exclusion to a given population, data analysis and results presentation will be provided in a statistical analysis plan (SAP), a separate document provided by the Biostat Sp. z o. o. prior to the final database lock.

1.1.10.1 INTERIM ANALYSES

An interim analysis is planned after half of the randomized subjects reach primary endpoint (functional status assessment with modified Rankin scale on day 90) in each study arm (3 interim analyses in the study in total).

1.1.10.2 IDMC ANALYSES

IDMC will review safety and efficacy data on the ongoing basis (i.e., adverse events of special interest) and periodically and it will be described in the Safety Management Plan specific for the STROACT study and IDMC charter.

In the study group (arm) B and C an interim safety analysis is planned after the first six patients compete study treatment with Visit 3 (Day 7) data available for all patients.

1.2 SCHEMA

The STROACT study comprises 3 parallel arms (study groups) based on NOAC received before the current AIS event and overall study design is shown in **Fig 1**. Patient screening and enrolment process is outlined in **Fig.2**.

Figure 1. Study diagram.

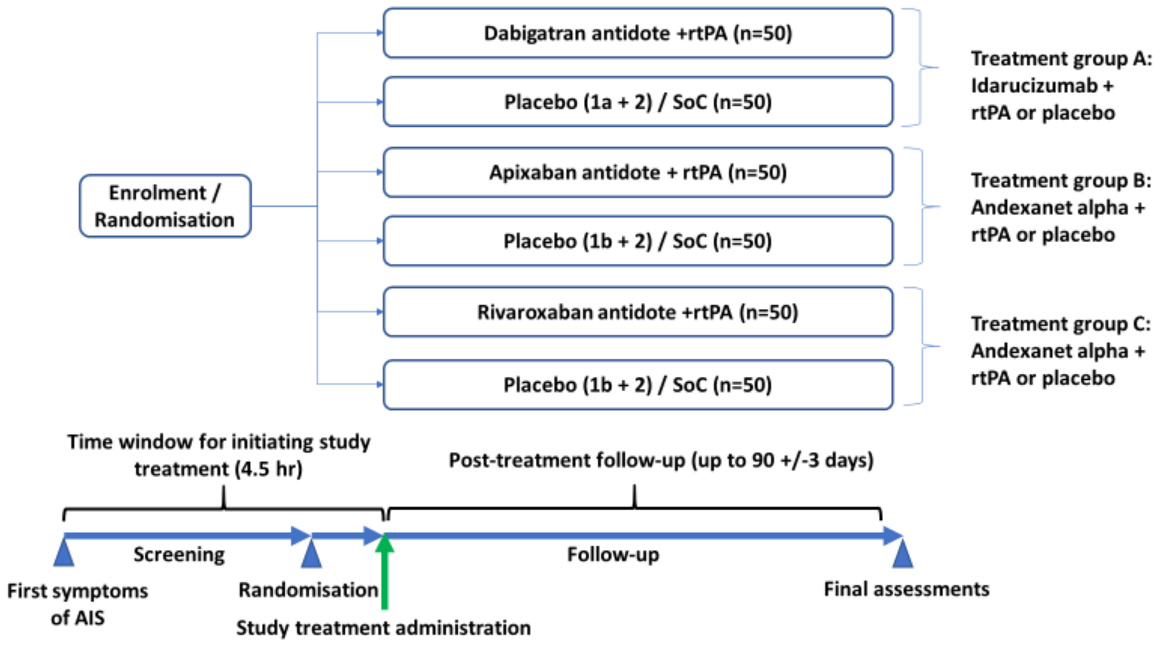

<u>Abbreviations:</u>
AIS – acute ischaemic stroke; rtPA – recombinant tissue plasminogen activator; SoC – standard of care

Figure 2. Patient screening and enrolment.

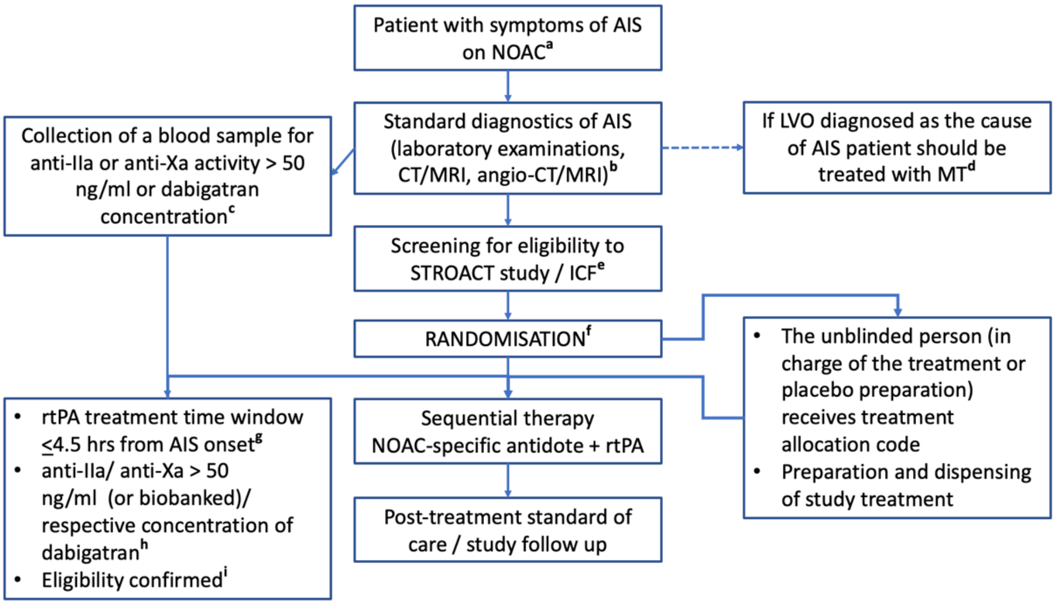

Abbreviations:

AIS – Acute Ischaemic Stroke; CT – Computed Tomography; LVO – Large Vessel Occlusion; MRI – Magnetic Resonance Imaging; MT – Mechanical Thrombectomy; NOAC – non-vitamin K antagonist oral anticoagulant; rtPA – recombinant tissue plasminogen activator; USG – ultrasound scan.

Annotations:

a – Patients with acute ischaemic stroke (AIS), with no previous significant disability (0-2 points on the modified Rankin Scale [mRS]), and on NOAC treatment due to any reason, usually as a prevention of cardioembolic events, are potential candidates for inclusion in the STROACT study.

b – The AIS standard diagnostic procedures must follow national / local guidelines on stroke diagnosis and management; if the stroke severity at admission in NIHSS is ≥ 6 points, then a large vessel occlusion (LVO) must be excluded in angio-CT or angio-MRI or invasive angiography (catheter) as LVO may be an indication for primary mechanical thrombectomy (MT).

c – Blood sample for determination of anti-IIa / anti-Xa activity or dabigatran blood concentration in the respective arm of the study (corresponding to NOAC plasma concentration) must be obtained as soon as possible. However, in patients on dabigatran, it is possible to enrol such a subject into the study based solely on a patient's or witness' report on the last intake of the anticoagulant ie, when it was administered within the last 24 hours preceding initiation of the study treatment. In these situations it is obligatory to collect and store blood for post-hoc anti-IIa testing. The procedure is described in the Laboratory Manual.

d – Patients with AIS due to LVO will not be included in the STROACT study (such patients should be considered for the primary mechanical thrombectomy [MT]).

e – Informed consent to participate in the STROACT study should be obtained as soon as possible after admission, if preliminary assessment indicates that the patient may be included; due to critical impact of time from AIS onset to initiating intravenous thrombolysis (IVT), final verification of participant's eligibility (all inclusion and exclusion criteria verified) can take place after randomisation and before initiating study treatment administration.

f – Randomisation will be performed in the eCRF. Randomisation should be performed within 3.0 hrs from AIS symptoms onset to allow timely preparation of the allocated study treatment (rtPA or corresponding placebo) to be initiated within 4.5 hr from the IS symptoms onset. After generating patient randomization number in the eCRF by the investigator, the unblinded person will receive notification about allocated study treatment and will prepare study treatment (active or placebo) based on notification generated via eCRF.

g – Infusion of the thrombolytic component of the investigated therapy scheme (rtPA or corresponding placebo) should be initiated within 4.5 hr (but not later than 6 hr) from the IS symptoms onset

h – Results of anti-IIa / anti-Xa activity testing (corresponding to NOAC plasma concentration) must be >50 ng/mL or appropriate dabigatran blood concentration in the respective arm of the study. However, in patients on dabigatran, it is possible to enrol such a subject into the study based solely on a patient's or witness' report on the last intake of the anticoagulant ie, when it was administered within the last 24 hours preceding initiation of the study treatment. In these situations it is obligatory to collect and store blood for post-hoc anti-IIa testing. The procedure is described in the Laboratory Manual.

i – To be administered with the study treatment or placebo, patient must meet all inclusion criteria and must not meet any of the exclusion criteria.

j – Study treatment is defined as sequential intravenous administration of NOAC-specific antidote (or placebo) and the infusion of rtPA (or corresponding placebo).

In the STROACT study, rtPA (both bolus and infusion) should be administered to a vain of the arm contralateral to the ischemic lesion (commonly paretic), whereas antidote (idarucizumab or both bolus and infusion of andexanet alfa) to a vain of the arm ipsilateral to the ischemic lesion. For many years of the use of rtPA for ischemic stroke, there have been no major or significant reports showing any differences between action of rtPA administered to ipsi- and contra-lateral arms (that might be potentially caused by some processes like for example local autonomic disturbances), whereas data on such potential associations for both anitidotes are poor.

k – After receiving study treatment patients will be assessed at 24 (+/4) hrs (Visit 2), 7 (+/-1) days (Visit 3), 30 (+/-2) days (Visit 4), and 90 (+/-3) days (Visit 5) after rtPA administration; study visits 0-3 are expected during patient hospitalisation (including emergency and stroke units); assessment visit 4 can be performed over the phone and visit 5 as an in-person visit in the hospital).

1.3 SCHEDULE OF ACTIVITIES (SOA)

**Study period**	**Screening / randomisation**	**Treatment phase**	**Post-treatment follow-up**			**End of study**
**Visit number**	**Visit 0**	**Visit 1**	**Visit 2**	**Visit 3**	**Visit 4**	**Visit 5**
**Visit day**	Day 1[a]	Day 1[b]	Day 2[c]	Day 7[c]	Day 30[c]	Day 90[c]
**Visit time window**	Pre- treatment	Treatment	±4 hrs	±1 day	±2 days	±3 days
**Type of study visit[d]**	Clinic	Clinic	Clinic	Clinic	Phone contact	Clinic
Informed consent[e]	X					
Inclusion / exclusion criteria	X					
Allocation to the study group[f]	X					
**Routine clinical procedures**						
Demography/baseline characteristics	X					
Medical/surgical history	X					
Concomitant treatments / medications	X					
Vital signs	X	X	X	X		(X) [q]
Physical examination	X					
Signs and symptoms of infection	X		X	X		(X) [q]
**Laboratory examinations**						
Haematology						
CBC and platelet count	X		X	X		
Coagulation parameters						
dabigatran concentration/ anti-IIa (in study group A)	X[h]	X[j]				
Anti-Xa (in study group B, C)	X[h]	X[i]				
INR, aPTT, fibrinogen	X		X	X		
Biochemistry panel						
Electrolytes	X		X			

**Study period**	**Screening / randomisation**	**Treatment phase**	**Post-treatment follow-up**			**End of study**
**Visit number**	**Visit 0**	**Visit 1**	**Visit 2**	**Visit 3**	**Visit 4**	**Visit 5**
**Visit day**	Day 1[a]	Day 1[b]	Day 2[c]	Day 7[c]	Day 30[c]	Day 90[c]
**Visit time window**	Pre- treatment	Treatment	±4 hrs	±1 day	±2 days	±3 days
**Type of study visit[d]**	Clinic	Clinic	Clinic	Clinic	Phone contact	Clinic
Glucose	X	X[t]	X			
Creatinine (eGFR)	X		X			
AlAT, AspAT, bilirubin	X		X			
Albumin	X		X			
CRP	X		X	X		
Troponin-I	X		X			
BNP/ NT-proBNP	X			X		
**Stroke clinical assessments**						
NIHSS	X		X	X		X
mRS	X			X	X	X
Barthel Index				X		X
MoCA				X		X
HADS				X		X
IQCODE				X		
CT	X		X			
MRI[k]	(X)[k, r]		(X) [r]	X		
CTA/MRA, angiography[l]	(X)[l, r]					
ECG	X[u]	X[u]	X[u]			
Echocardiography	X[s]	X[s]	X[s]	X[s]		
DUS of extracranial / intracranial arteries	X[s]	X[s]	X[s]	X[s]		
**Randomisation[m]**	X[m]					
Preparation of study treatment[n]	X[n]					

Study period	Screening / randomisation	Treatment phase	Post-treatment follow-up			End of study
**Visit number**	**Visit 0**	**Visit 1**	**Visit 2**	**Visit 3**	**Visit 4**	**Visit 5**
**Visit day**	Day 1[a]	Day 1[b]	Day 2[c]	Day 7[c]	Day 30[c]	Day 90[c]
**Visit time window**	Pre- treatment	Treatment	±4 hrs	±1 day	±2 days	±3 days
**Type of study visit[d]**	Clinic	Clinic	Clinic	Clinic	Phone contact	Clinic
**Study treatment administration[m]**						
NOAC-specific antidote (or placebo)		X				
rtPA (or placebo)		X[o]				
**Routine safety assessments**						
AEs	X	X	X	X	X	X
Safety endpoints		X[w]	X[w]	X[w]	X[w]	X[w]
Concomitant medications / therapy	X	X	X	X	X	X[p]
Pregnancy test[q]	X[q]					

<u>Abbreviations:</u>

AE – adverse event; BNP – brain natriuretic peptide; CBC – complete blood count; CRP – C-reactive protein; CT – Computed Tomography; CTA – CT angiography; DUS – Doppler ultrasound scan; ECG – Electrocardiogram; HADS – Hospital Depression and Anxiety Score; IS – Ischaemic Stroke; MoCA – Montreal Cognitive Assessment test; MRI – Magnetic Resonance Imaging; MRA – MRI angiography; mRS – modified Rankin Scale; NIHSS – NIH Stroke Scale; NOAC – non-vitamin K antagonist oral anticoagulant; NT-proBNP (N-terminal pro B-type natriuretic peptide); rtPA – recombinant tissue plasminogen activator;

<u>Annotations:</u>

a – AIS standard diagnostic (screening) procedures must follow national /local guidelines on stroke diagnosis and management; obtaining ICF; standard diagnostic procedures, allocation to the study group and randomisation should be completed **within 3.0 hrs of AIS onset.**

b – Study treatment includes sequential administration of NOAC specific antidote and thrombolytic treatment (rtPA) or corresponding placebo and administration of the thrombolytic component **should be initiated with 4.5** hr window (<u>no later than 6h</u>) from AIS onset. The visit ends when all components of the study treatment are administered.

c – Visit 2 must occur at 24 hour (± 4 hours), visit 3 must occur on day 7 (± 1 day), visit 4 must occur on day 30 (± 2 days), visit 5 must occur on day 90 (± 3 day) after the start of administration of the thrombolytic component of study rtPA (or an appropriate placebo).

d – Study visits 0-3 are expected during hospitalisation (including stay in the emergency unit and neurology department); assessment visits 4 can be performed over the phone and 5 as an in person visit in the center (hospital).

e – Patients whose worsening neurological deficit makes it impossible to sign the consent form may give their oral consent to participate in the study. Patients with aphasia and/or other speech disorders may be included in the study if in the opinion of the qualifying physician are able to understand the idea of the study. For detailed information please refer to **the procedure of obtaining informed consent described in the Clinical Trial Protocol (Section 6.3).**

f – After obtaining ICF patient will be allocated to one of the study group based on NOAC taken; group A – dabigatran; group B – apixaban, group C – rivaroxaban).

h – The first blood sample for determination of anti-IIa / anti-Xa activity or dabigatran blood concentration in the respective arm of the study (corresponding to NOAC plasma concentration) should be collected and processed immediately after obtaining ICF; patients with NOAC plasma concentration ≤50 ng/ml do not qualify for antidote administration and will be treated according to standard of care, which may include thrombolytic treatment. However, in patients on dabigatran, it is possible to enroll such a subject into the study based solely on a patient's or witness' report on the last intake of the anticoagulant ie, when it was administered within the last 24 hours preceding initiation of the study treatment. In these situations, it is obligatory to collect and store blood for post-hoc anti-IIa testing. The procedure is described in the Laboratory Manual.

i – The second blood sample for determination of anti-Xa (corresponding to NOAC plasma concentration) should be taken after NOAC-specific antidote bolus infusion (or corresponding placebo); the third blood sample should be collected after completion of rtPA (or corresponding placebo) administration.

j- Please note that in the STROACT trial we plan to estimate anti-IIa activity only in the pre-treatment stage of the study (a single measurement: either prospective ie, when treatment decisions are based on the blood test result, or retrospective ie, in cases where treatment decisions are based solely on drug administration history). Whereas anti-IIa 2nd (after infusion of NOAC-specific antidote bolus or corresponding placebo) and 3rd measurements (after completion of rtPA or corresponding placebo administration) are only facultative in this arm of the project. The second and third dabigatran blood measurements should not be performed.

k – Brain CT is preferred.

l – Mandatory examination for patients with **NIHSS ≥6** or other clinical suspicion of Large Vessel Occlusion (LVO) and qualifying the patient to mechanical thrombectomy (exclusion from STROACT study).

m – Randomisation should be performed within 3.0 hrs from IS symptoms onset to allow timely preparation of the allocated study treatment to be initiated within 4.5 hr (not later than 6.0h) from the IS symptoms onset; **if randomisation time is >3.0 hrs from the onset of IS symptoms investigator should consider patient withdrawal from the study.**

n – Preparation of the study treatment should be initiated immediately after receipt of the laboratory test results of anti-IIa or anti-Xa activity >50 ng/mL or dabigatran concentration for the respective arm of the study. However, in patients on dabigatran, it is possible to enrol such a subject into the study based solely on a patient's or witness' report on the last intake of the anticoagulant ie, when it was administered within the last 24 hours preceding initiation of the study treatment. The procedure is described in the Laboratory Manual.

o – Infusion of the thrombolytic component of the study treatment (rtPA or corresponding placebo) should be initiated within 4.5 hr from the AIS onset, but **no later than within 6 hours from AIS onset**, otherwise participant should be withdrawn from the study.

p – In case of initiating thromboembolic prophylaxis, i.e., starting antiplatelet agents, heparins or OAC/NOAC, the information about type of medication, dosage and time of initiating treatment should be entered in patient's medical records and on eCRF (see Section 7.6.1)

q – In women of childbearing potential urine pregnancy test must be performed or a blood pregnancy test if urine cannot be obtained. Females of childbearing potential and women in a postmenopausal state: females of childbearing potential and women with no menses for a period < 12 months must have a negative pregnancy test at Visit 0 and have to agree not to start a pregnancy from the signature of the informed consent up to the Final Visit, using an appropriate birth control method such as combined oestrogen-progestin containing hormonal contraceptives (e.g., oral, injectable, transdermal), progestin-only hormonal contraceptives (e.g., oral, injectable, implantable), intrauterine device (IUD) or intrauterine hormone-releasing system (IUS) in combination with male condom, bilateral tubal occlusion, vasectomised partner, sexual abstinence.

Woman of childbearing potential (WOCBP): i.e., fertile, following menarche and until becoming post-menopausal, unless permanently sterile. Permanent sterilization methods include hysterectomy, bilateral salpingectomy and bilateral oophorectomy.

Postmenopausal state is defined as no menses for 12 months without an alternative medical cause.

r – Assessments in brackets are optional (to allow data collection if available), and if they interfere with standard diagnostic assessments, they are not clinically justified, or may significantly delay initiation of investigational treatment may not be performed; missing data for these assessments will be not considered as protocol deviations.

s - The test should be performed at least once during hospitalization (V0-V3)

t - Obligatory only in cases where the screening value was beyond that defined in inclusion/exclusion criteria.

u - ECG must be performed preferably within the first 24 hours from AIS but not later than 72 hours.

w- Review of safety endpoints: any symptomatic intracranial haemorrhage (sICH), any life threatening extracranial bleeding, recurrent or new acute thromboembolic stroke, any life threatening thromboembolic complications, any significant allergic reactions assumed to be related with the administered substances (antidotes, rtPA).

2 INTRODUCTION

2.1 CURRENT RESEARCH AND STUDY RATIONALE

Stroke is the most common cause of permanent complex disability in adults and one of the most common causes of death worldwide. Around 85% of all strokes are related to acute focal cerebral ischemia (acute ischaemic stroke, AIS) that might be caused by acute occlusion or critical stenosis of one or more intracranialor extracranial arteries (Lindsay MP et al, 2019).

More than 20% of AIS are cardioembolic, most commonly due to atrial fibrillation (AF) (ESC Guidelines, 2016).

Estimates of the Polish Neurological Society (PNS) and the Polish Cardiac Society, as well as preliminary reports from researchers of the NOMED-AF project, suggest that AF (symptomatic and non-symptomatic) affects >23% of the population in the age group > 65 years.

If AF is accompanied by one of the other specific risk factors of AIS, it is an indication for oral anticoagulation (OAC) (CHADS2VASc classification) (Lip GY et al, 2010). The benefit-risk evaluation of OAC administration (reduction of AIS risk vs. increased risk of clinically relevant bleeding) in these patients is clearly positive. However, although OAC therapy (among which the most commonly used are the so-called new OAC [NOAC], i.e., rivaroxaban, dabigatran or apixaban, and rarely others) reduces the risk of AIS by more than 80%, it still significantly higher than in the general population (1-2% of the annual risk). The incidence of AIS in patients using OAC therapy in Poland has been roughly estimated at 8,000 annually, which includes approximately 5,000 patients on NOAC. Some of these patients might be treated invasively with aspiration and/or mechanical thrombectomy, others (OAC concentration measured by specific test below 50 ng/ml) might be still eligible for thrombolysis, but most of them remain without any approved reperfusion therapeutic possibility as per current clinical management guidelines (PNS Guidelines, 2019).

Recent approvals for NOAC-specific antidotes (idarucizumab, andexanet alfa) provide a therapeutic option for rapid reversal of NOAC anticoagulant effects for serious/life-threatening or uncontrolled bleedings, emergency surgery and/or other urgent procedures (RE-VERSE AD, ANNEXA-4) (Ondexxya SmPC, 2020) (Praxbind SmPC, 2020) (Connolly SJ et al, 2019) (Pollack CV et al, 2017). However, there is poor (dabigatran – idarucizumab) or no (apixaban / rivaroxaban – andexanet alfa) data from clinical trials/studies supporting efficacy and safety of the NOAC-specific antidote + intravenous rtPA sequential therapy.

The aim of this study is to develop and assess the first causative (reperfusion sequential) therapy for this group of patients based on combined anticoagulant reversal and fibrinolytic agents.

Idarucizumab is a humanized monoclonal antibody fragment with structural similarity to thrombin but lacks enzymatic activity. Idarucizumab irreversibly binds to dabigatran (approximately 350-fold stronger than thrombin) with rapid and complete reversal of anti-IIa activity and with no demonstrated pro-coagulation activity. The maximum reversal effect is observed few minutes after antidote administration – approximately 88-98% of anti-IIa activity reduction (Praxbind SmPC, 2020).

Andexanet alfa is a modified recombinant protein analogue of factor Xa. As a result of the removal of the gamma-carboxy-glutamic acid domain (Gla), the protein's ability to attach to the prothrombinase complex has been removed while retaining the native structure and binding factor Xa inhibitors with high affinity (binding and sequestration of the factor Xa inhibitors with minor contribution form the inhibition of tissue factor pathway inhibitor, TFPI). Andexanet alfa is the first approved specific reversal agent for apixaban and rivaroxaban which decreases anti-Xa activity

within two minutes of bolus administration and return to clinically significant levels approximately two hours after end of the infusion (Ondexxya SmPC, 2020).

The STROACT study, a randomized, placebo-controlled clinical trial, will investigate the therapy scheme that includes the two following components administered sequentially:

For the study group A (patients on dabigatran):

- NOAC-specific antidote to reverse the NOAC activity administered intravenously, followed by intravenous thrombolysis with rtPA (bolus and infusion immediately thereafter).

For the study group B (patients on apixaban) and C (patients on rivaroxaban):

- intravenous bolus of NOAC-specific antidote to reverse the NOAC activity, followed by
- intravenous infusion of NOAC-specific antidote initiated parallelly to rtPA bolus administration (if possible) or immediately before the rtPA bolus administration
- intravenous bolus of rtPA (preferably into the contralateral arm or another distant vain) administered parallelly to NOAC-specific antidote infusion initiation (if possible) or immediately after the NOAC-specific antidote infusion initiation
- intravenous infusion of rtPA (initiated immediately after the completion of the rtPA bolus).

The reperfusion treatment with alteplase (rtPA) will be administered intravenously according to the standard protocol at a dose of 0.9 mg/kg of body weight (maximum 90 mg); 10% of the calculated dose in a rapid intravenous injection (bolus) within 1-2 minutes, and 90% of the calculated dose in an intravenous infusion over 1 hour (syringe pump) after exclusion of potential contraindications in accordance with the guidelines of the American Heart Association / American Stroke Association 2018 and 2019, the European Stroke Organization 2019 and experts of the Polish Neurological Society 2019 (PNS Guidelines, 2019) (Actilyse SmPC, 2019).

So far, only a few clinical reports described the effects of administration of idarucizumab in patients with AIS prior to reperfusion therapy with intravenous thrombolysis. No data has been found on the use of andexanet alpha as proposed in this study.

2.2 BACKGROUND

The burden of cardiovascular and cerebrovascular diseases has been constantly growing over the last decade and is expected to increase further as the world population ages. There were over 13.7 million new stroke cases in 2016 with estimated 5.5 million deaths (Feigin VL et al, 2019). The age-standardized incidences of stroke were 185 (171.98-198.75) per 100 000 person-years, and 203 (189.24-218.16) per 100 000 person-years after age-adjustment. It is estimated that one in four adults will experience a stroke in their lifetime (Lindsay MP et al, 2019).

The incidence of AIS in Poland is estimated at 70,000 annually.

Ischemic stroke is defined as abrupt onset of neurological deficit related to acute focal cerebral ischemia that might be caused by acute occlusion or critical stenosis of one or more intracranial or extracranial arteries. The acute occlusion of the extracranial or cerebral artery results in substantial tissue hypoperfusion and results in ischemia, and then, potentially, infarction. Final volume of the infarcted brain depends on many factors, mainly on the size and anatomical characteristics of the occluded vessel, severity of cerebral blood flow reduction, time from onset to recanalization, various processes in the brain (brain tissue molecular characteristics), and many other local and systemic factors. The area of the ischemic brain considered as non-salvageable is called the "core". The hypoperfused tissue immediately surrounding the core is called "penumbra", which is the main portion of the "tissue at risk" – the potentially salvageable brain after recanalization and reperfusion.

The specific stroke treatment that might lead to recanalization and reperfusion is essential to improve clinical outcome and reduce mortality. The aim of reperfusion therapies in ischemic stroke is to regain functional excellence or independence defined as 0 or 1-2 points on modified Rankin performance scale, respectively. The introduction of two specific therapies for acute brain ischemia – intravenous thrombolysis (IVT) and mechanical thrombectomy (MT), combined with comprehensive, interdisciplinary care provided by stroke units, significantly improved chances for such an outcome (Powers WJ et al, 2019).

Intravenous thrombolysis with recombinant tissue plasminogen activator (rtPA) is the mainstay method of acute ischemic stroke therapy up to 4,5 hours from its onset, with the largest benefits associated with possibly earliest treatment initiation (Emberson J et al, 2014).

More than 20% of AIS are cardioembolic, most commonly due to atrial fibrillation (AF). If AF is accompanied by one of the other specific risk factors of AIS, it is an indication for oral anticoagulation (OAC), or, rarely, in special cases, for low molecular weight (LMWH) or unfractionated heparin (CHADS2VASc classification). The use of oral anticoagulants (OAC) is steadily increasing with the aging society and improving access to medical care (Hindricks G et al, 2020).

The incidence of AIS in patients using OAC therapy in Poland has been roughly estimated at 8,000 annually, which includes approximately 5,000 patients on NOAC. Some of these patients might be treated invasively with aspiration and/or mechanical thrombectomy, others (OAC concentration measured by specific test below 50 ng/ml) might be still eligible for thrombolysis, but most of them remain without any approved reperfusion therapeutic possibility as per current clinical management guidelines (PNS Guidelines, 2019).

The aim of this study is to develop and assess the first causative (reperfusion sequential) therapy for this group of patients based on combined anticoagulant reversal and fibrinolytic agents.

2.3 RISK/BENEFIT ASSESSMENT

The STROACT (STRoke on Oral AntiCoagulants for Thrombolysis) is the first ever study to assess the efficacy and safety of sequential reperfusion therapy with the following scheme: non-vitamin K antagonist oral anticoagulant fast-acting specific reversal agent and intravenous thrombolysis therapy with rtPA.

2.3.1 KNOWN POTENTIAL RISKS

Patients with AIS are the population with multiple and various comorbidities, and high total cardiovascular risk.

The most severe complication related to rtPA administration is symptomatic intracerebral haemorrhage (sICH), which occurs in 3-7% of all treated patients, and is associated with poor functional outcome (Hacke W et al, 2008) (Sandercock P et al, 2012) (Karaszewski B et al, 2015) (Yaghi S et al., 2014). Meta-analysis of nine trials of rtPA in ischemic stroke (Whiteley WN et al, 2016) showed increased risk of type 2 parenchymal haemorrhage (PH2), of haemorrhage as per SITS-MOST definition, and fatal intracerebral haemorrhage in IVT group.

In REVERSE-AD trial (A Study of the RE-VERSal Effects of Idarucizumab on Active Dabigatran), a multicentre, prospective, open-label study, patients with life-threatening or uncontrolled bleeding and patients who required emergency surgery or other urgent procedures, were treated with 5.0 g of intravenous idarucizumab. Median maximum reversal value was 100% in the first 4 hrs. Most patients achieved complete reversal as measured by ECT (82%), or dTT (99%). The adverse reactions reported in ≥5% of patients were constipation and nausea. In 33 (6.56%) patients reported thrombotic

events, and most of these patients were not on antithrombotic therapy at the time of the event (Pollack CV et al, 2017).

In ANNEXA-4 trial (The Andexanet Alfa, a Novel Antidote to the Anticoagulation Effects of Factor Xa Inhibitors), a single-group cohort study, patients with acute major bleeding – 64% intracranial and 26% gastrointestinal – were treated with andexanet alfa in intravenous bolus over 30 minutes, followed by continuous infusion for 120 min. Median anti-FXa activity decreased was 92% for both apixaban and rivaroxaban patients. There were 34 patients (10%) with a thrombotic event during 30-day follow-up period (Connolly SJ et al, 2019).

2.3.2 KNOWN POTENTIAL BENEFITS

Specific AIS treatment therapies – intravenous thrombolysis (IVT) and mechanical thrombectomy (MT), combined with comprehensive, interdisciplinary care provided by stroke units significantly improve chances for excellent and good functional outcomes (mRS 0-1 and 0-2, respectively).

Reversing NOAC activity with administration of fast-acting specific reversal agents (antidotes): idarucizumab for dabigatran, and andexanet alfa for apixaban or rivaroxaban, has been shown to be an effective and safe emergency treatment for major, life-threatening bleeding complications of NOAC therapy, or as a prevention of bleeding in patients requiring emergency surgery, or invasive procedures (Ondexxya SmPC, 2020) (Praxbind SmPC, 2020).

Taking that maximum reversal effect of idarucizumab is observed within few minutes from bolus administration, and high proportion of patients show complete neutralization of anticoagulant activity (Ondexxya SmPC, 2020) (Praxbind SmPC, 2020). it is possible that the sequential reperfusion intravenous therapy with rtPA soon after idarucizumab might result in outcomes similar to those observed in large rtPA stroke trials.

Similar benefits might be expected from reversing anti-Xa activities by andexanet alfa followed by administration of intravenous rtPA. However, there is no single empiric data of this therapeutic approach, and the mechanisms of action with all pharmacodynamic and pharmacokinetic characteristics, and potential harms related to not only bleeding, but also thromboembolic complications as assumed from complex pathophysiological relations, are different for andexanet alfa in comparison to idarucizumab.

2.3.3 ASSESSMENT OF POTENTIAL RISKS AND BENEFITS

The STROACT is the first ever study to assess the efficacy and safety of sequential reperfusion therapy with the following scheme: non-vitamin K antagonist oral anticoagulant fast-acting specific reversal agent and intravenous thrombolysis therapy with rtPA.

There is an urgent clinical need to develop and assess the first causative (reperfusion) therapy for ischemic stroke patients on NOAC with anti-IIa/anti-Xa activity > 50 ng/ml or respective blood concentration of dabigatran. To minimize all peri-procedural risks and maximize patients' safety, we developed detailed and clear inclusion and exclusion criteria in accordance with EBM. The main safety endpoint is the rate of symptomatic intracranial haemorrhages (sICH). To follow-up these, in the management protocol we incorporated two neuroimaging testing (scanning) during the first week after randomisation: the first CT/MRI is going to be performed at 24 hours ± 4 hours after rtPA bolus, and MRI - 7 days ± 1 day (24 hours) from rtPA bolus.

All hospitals participating in the STROACT study are comprehensive stroke centres (CSC) with extensive experience in specific acute ischemic stroke treatments.

Incoming clinical safety data will be monitored and reviewed by IDMC to reassure patients' safety and scientific integrity of the study.

3 AIMS, OBJECTIVES AND ENDPOINTS

This study aims to evaluate the efficacy and safety of the reperfusion thrombolytic therapy with rtPA in patients with acute ischaemic stroke (AIS) on non-vitamin K antagonist oral anticoagulants (NOAC), after neutralisation of the NOAC anticoagulant activity (administration of a specific antidote) who do not qualify for mechanical thrombectomy.

3.1 PRIMARY OBJECTIVE

**Objective**	**Outcome measure / Endpoint**
**Efficacy**	
To assess the efficacy of the investigational therapy in patients with acute ischaemic stroke on the non-vitamin K antagonist oral anticoagulants compared to placebo using modified Rankin Scale (mRS).	Proportion of patients with excellent and good functional outcome assessed with modified Rankin scale (mRS) (mRS 0-1 and 0-2, respectively) at 90 days (+/- 3 days) after study treatment administration.

3.2 SECONDARY OBJECTIVES

**Objectives**	**Outcome measures / Endpoints**
**Efficacy**	
To assess the efficacy of the investigational therapy in patients with acute ischaemic stroke on the non-vitamin K antagonist oral anticoagulants with pre-stroke functional status of 0-1 points in mRS compared to placebo using modified Rankin Scale (mRS).	Proportion of patients with excellent functional status assessed with modified Rankin scale (mRS) (mRS 0-1) at 90 days (+/- 3 days) after investigational treatment administration.
To assess the efficacy of the investigational therapy in patients with acute ischaemic stroke on the non-vitamin K antagonist oral anticoagulants compared to placebo using NIHSS score.	Change in NIHSS score from baseline assessed at 7 (+/-1 day), and 90 days (+/- 3 days) after investigational treatment administration.
**Safety**	
To assess the incidence of fatal events after the investigational therapy in patients with acute ischaemic stroke on the non-vitamin K antagonist oral anticoagulants compared to placebo.	Incidence of deaths:<br>- Deaths from any cause<br>- Deaths subdivided by cause<br>at 7 (+/-1 day), 30 (+/-2 days) and 90 days (+/- 3 days) after investigational treatment administration.
To assess the incidence of non-fatal events after the investigational therapy in patients with acute ischaemic stroke on the non-vitamin K antagonist oral anticoagulants compared to placebo.	Incidence of non-fatal events defined as<br>- Recurrent ischaemic stroke<br>- Haemorrhagic stroke (ICH or SAH)<br>- Neurological deterioration (NIHSS)<br>at 7 (+/-1 day), and 90 days (+/- 3 days) after investigational treatment administration.

To evaluate safety of the investigational therapy in patients with acute ischaemic stroke on the non-vitamin K antagonist oral anticoagulants compared to placebo assessed with neuroimaging.	Rate and severity of early (symptomatic and asymptomatic) intracranial haemorrhage detected by neuroimaging:<br>- CT or MRI at 24hrs (+/- 4hrs) after investigational treatment administration<br>- CT or MRI at 7 days (+/- 1 day) after investigational treatment infusion<br>and assessed according to European Cooperative Acute Stroke Study (ECASS II) classification.<br>Note: Symptomatic Intracranial Haemorrhage (sICH) defined as deterioration of stroke severity in NIHSS of ≥ 4 points with parenchymal haemorrhage type 2 in neuroimaging.
To assess characteristics of major clinically significant extracranial bleedings after the investigational therapy in patients with acute ischaemic stroke on the non-vitamin K antagonist oral anticoagulants compared to placebo.	Incidence and severity of major extracranial haemorrhages defined as:<br>- fatal<br>- severe enough to require transfusion or surgery<br>- an absolute decrease in haemoglobin > 5 g/dL<br>- a decrease in haematocrit of > 15%<br>- bleeding in persistent or temporary serious disability)
To assess overall safety profile of the investigational therapy in patients with acute ischaemic stroke on the non-vitamin K antagonist oral anticoagulants compared to placebo.	Incidence and category of AEs reported during the study.

3.3 EXPLORATORY OBJECTIVES

Objective	Outcome measure / Endpoint
To assess BNP or NT-proBNP plasma levels after the investigational therapy in patients with acute ischaemic stroke on the non-vitamin K antagonist oral anticoagulants after administration of a specific reversal agent compared to placebo.	Change in BNP or NT-proBNP plasma concentration at Day 7 compared to pre-treatment levels.
To assess functional independence after the investigational therapy in patients with acute ischemic stroke on the non-vitamin K antagonist oral anticoagulants compared to placebo using Barthel Index scale.	Change in Barthel Index score measured at Day 90 compared to Day 7.
To assess cognitive function after the investigational therapy in patients with acute ischemic stroke on the non-vitamin K antagonist oral anticoagulants compared to placebo using MoCA scale.	Change on MoCA scale measured at Day 90 compared to Day 7.
To assess pre-stroke cognitive function after the investigational therapy in patients with acute ischemic stroke on the non-vitamin K antagonist oral anticoagulant compared to placebo using IQCODE scale.	Assessment of pre-stroke cognitive impairment measured by IQCODE scale at Day 7.

To assess “mental” condition (i.e. depression and anxiety) after the investigational therapy in patients with acute ischemic stroke on the non-vitamin K antagonist oral anticoagulants compared to placebo using HADS scale.	Change on HADS scale measured at Day 90 compared to Day 7.
To assess duration of the hospitalisation after the investigational therapy in patients with acute ischemic stroke on the non-vitamin K antagonist oral anticoagulants compared to placebo.	Number of days from the hospital admission to discharge.

4 STUDY DESIGN

4.1 OVERALL DESIGN

STROACT is a multicentre, parallel group, randomized, double-blind, placebo-controlled, non-commercial clinical trial to evaluate the efficacy and safety of sequential reperfusion thrombolytic therapy with rtPA (alteplase) in patients with acute ischaemic stroke (AIS) on non-vitamin K antagonist oral anticoagulants (NOAC: dabigatran, apixaban, or rivaroxaban) after administration of a specific antidote (idarucizumab for dabigatran, and andexanet for apixaban or rivaroxaban). Outline of the study design and patient flow in the study is presented in Section 1.2 the graphic form in Fig 1 (Study diagram) and Fig. 2 (Patient screening and enrolment).

The STROACT study comprises 3 study groups with patient allocation based on NOAC taken before the AIS onset. Specific study interventions per study group are then as follows:

Study group A (patients receiving dabigatran and with high plasma anticoagulant activity): intravenous administration of idarucizumab followed by an infusion of rtPA (or corresponding placebo for each component of the intervention).

Study group B (patients receiving rivaroxaban and with high plasma anti-Xa activity): intravenous administration of andexanet alfa followed by an infusion of rtPA (or corresponding placebo for each component of the intervention).

Study group C (patients receiving apixaban and with high plasma anti-Xa activity): intravenous administration of andexanet alfa followed by an infusion of rtPA (or corresponding placebo for each component of the intervention).

Study participants allocated to A, B, or C study group, will be randomized in 1:1 ratio to receive either active treatment or placebo (comparator). All patients who enter the STROACT study, whether allocated to active treatment or control, must be managed according to local acute stroke care protocols, in the same clinical environment. Such protocols are not specified by the clinical trial protocol but will generally include the components of effective stroke unit care.

Patients with symptoms of an acute ischaemic stroke (AIS) admitted to the hospital with no significant disability prior to the current cerebrovascular incident (0-2 points on the modified Rankin Scale [mRS]), and on NOAC treatment as a prevention of thromboembolic events will undergo standard diagnostic procedures and may be considered for inclusion in the STROACT study. Informed consent for participation in the study (signature of the informed consent form [ICF]) must be obtained as soon as possible when the patient is considered a candidate for the study (see Section 6.3).

After signing ICF and preliminary confirmation of eligibility for the study (see Section 5 and 6) patients will be allocated to one of the 3 parallel study groups (A, B or C) based on NOAC being taken before the current cerebrovascular event.

All patients MUST have a pre-enrolment brain CT scan (or MRI) to exclude an intracranial haemorrhage. In addition, patients with stroke severity ≥6 points in NIHHS, or with other clinical indicators suggesting large vessels occlusion (LVO), for example vision, aphasia, and neglect (VAN) assessment, will undergo angio-CT (or angio-MRI) of cerebral arteries to confirm or exclude LVO; patients with LVO may qualify to mechanical thrombectomy (MT) and will not be included in the study.

A blood sample will be obtained to determine anti-IIa/anti-Xa activity (or respective blood concentration of dabigatran in the respective arm of the study), which is assumed to be directly proportional to the plasma concentration of NOAC. Patients with anticoagulant activity >50 ng/ml (or respective blood concentration of dabigatran in the respective arm of the study) qualify to the study treatment i.e., to NOAC-specific antidote administration (followed by intravenous rtPA therapy); patients with anticoagulant activity level ≤50 ng/mL may qualify to receive thrombolysis

and eventually will not be included in the STROACT study. However, in patients on dabigatran, it is possible to enrol such a subject into the study based solely on a patient's or witness' report on the last intake of the anticoagulant ie, when it was administered within the last 24 hours preceding initiation of the study treatment. In these situations it is obligatory to collect and store blood for post-hoc anti-IIa testing. The procedure is described in the Laboratory Manual.

Eligible patients allocated to each study group will be randomised at 1:1 ratio to receive experimental treatment or corresponding placebo (see section 6.4). Study treatment is composed of sequential intravenous administration of NOAC-specific antidote (or placebo) followed by the infusion of rtPA or corresponding placebo.

Standard diagnostic procedures, allocation to the study group and randomisation should be completed within 3.0 hrs from AIS symptoms onset to minimise the risk of delay to start of the study treatment. Randomisation scheme will include severity of the IS at randomisation (<6 points, and ≥ 6 points in NIHSS) as a stratification factor in all 3 study groups (arms).

Administration of rtPA should be initiated within 4.5h time window calculated from the onset of AIS symptoms but no later than 6h in specific circumstances (correctly performed randomisation process and administration of the antidote, unavoidable factors delaying administration of rtPA beyond 4.5 hours).

After receiving study treatment patients will be assessed at 24 (+/-4) hrs (Visit 2), 7 (+/-1) days (Visit 3), 30 (+/-2) days (Visit 4), and 90 (+/-3) days after rtPA bolus treatment administration and collected data will be recorded in the eCRF (please see Schedule of Assessments [SoA] in Section 1.3)

An Independent Data Monitoring Committee (IDMC) will be established before the start patients' enrolment to monitor progress of the clinical study and to ensure participants' safety. This will include reviewing unblinded safety and the efficacy data, estimating the benefit-risk balance during the study and supervising the overall conduct of the study.

The composition and responsibilities of the IDMC will be described in the IDMC charter.

4.2 BLINDING

The STROACT study is designed as double-blind placebo controlled clinical trial; therefore, neither patient nor investigator will know the allocated study treatment. For further details please refer to Section 7.3.2.

4.3 SCIENTIFIC RATIONALE FOR STUDY DESIGN

The aim of this study is to develop and assess the first causative (reperfusion) therapy for this group of patients based on combined anticoagulant reversal and fibrinolytic agents. The STROACT clinical trial is a multicentre, parallel groups, randomised, double blind, placebo-controlled study evaluating efficacy and safety of a new intervention consisting of sequential intravenous administration of NOAC-specific reversal agent and thrombolysis with rtPA.

The overall rationale for the STROACT study is summarised in Section 2.1. In this section rationale for the selected elements of study design are discussed in more detail.

The standard outcome measures proposed in the STROACT study include those recommended for clinical trials to evaluate the efficacy and safety of the investigational therapy and characterise risk-benefit in the high risk patient population with AIS on the NOAC treatment (Kerr DM et ala, 2012) (Meyer L et al, 2020).

The modified Rankin Scale (mRS) is a 7-level ordered categorical scale evaluating the degree of disability or dependency in the daily activities of patients who suffered a stroke or other causes of neurological disability with scores ranging from 0 (fully independent) to 6 (dead) (see Section 9.1.2 and Section 14.2.2). The mRS is the recommended outcome measure in randomized clinical trials to

assess the efficacy of proposed intervention and the proportion of patients with excellent and good functional status mRS 0-1 and 0-2, respectively at 90 days (+/-3 days) after study intervention administration (Nunn A et al, 2016) (Saver JL, 2011) (refs). The mRS has been selected as the outcome for the primary efficacy endpoint in the STROACT study.

The National Institutes of Health Stroke Scale (NIHSS) is a systematic assessment tool that provides a quantitative measure of stroke-related neurologic deficit (see Section 9.1.1 and Section 14.2.1); NIHSS assessment will be included in eligibility, for stratification at randomization and outcome measure (secondary endpoint).

The evaluation of the overall safety of the sequential therapy is critical for determining benefit-risk of the proposed treatment. The following outcomes will be monitored and evaluated during the study:

- Mortality (death from any cause, and subdivided by cause)
- Incidence of non-fatal events (recurrent ischaemic stroke, neurological deterioration assessed in NIHSS)
- Incidence and characteristics of early intracranial haemorrhage (detected in CT/MRI and assessed according to ECASS II classification)
- Incidence and severity of extracranial bleeding

For details of efficacy and safety outcome measures please refer to Section 3 and Section 9.

The IDMC will be established and provide oversight and guidance during the study conduct to ensure patient safety and data integrity of STROACT clinical trial (see Section 10.3.3), in addition to study oversight by EC and SC (see Section 10.3.1 and Section 10.3.2).

4.4 JUSTIFICATION FOR DOSE

Study intervention in the STROACT study is defined as a sequential intravenous administration of NOAC-specific reversal agent / antidote (idarucizumab or andexanet) and rtPA (alteplase) or corresponding placebo.

All components of the study intervention (combination / sequential therapy) are commercially available medicinal products. Dosage and administration will follow the current product SmPC /local prescribing information. For andexanet alfa dosing two levels of anti-Xa activity and time from the last dose will guide selection of low or high dose of andexanet alfa in the sequential therapy.

4.4.1 ALTEPLASE

Alteplase (rtPA) will be administered according to standard dosing protocol approved and recommended for thrombolysis in patients with AIS (Actilyse SmPC, 2019) (Powers WJ et al, 2019).

For detailed information on dosage and administration please refer to Section 7.1.2

4.4.2 IDARUCIZUMAB

Idarucizumab is a specific, fast acting reversal agent for dabigatran anticoagulant activity and will be used in according to the standard dosing instructions approved for emergency surgery/urgent procedures (Praxbind SmPC, 2020)

For detailed information on dosage and administration please refer to Section 7.1.2.

4.4.3 ANDEXANET ALFA

Andexanet alfa is a specific, fast-acting, reversal agent for apixaban and rivaroxaban anticoagulant activity and will be administered in according to the standard dosing instructions approved for emergency surgery/urgent procedures, adjusted for results of anti-Xa activity assessed at admission

and information about the last dose taken. Current dosing instructions for andexanet alfa refer to the dose of apixaban or rivaroxaban taken and time from the last dose (Ondexxya SmPC, 2020)., however availability of assays to determine specific NOAC drug concentrations correlating with anti-Xa activity and knowledge of PK/PD profile of apixaban and rivaroxaban can support optimal clinical decision-making on dosing of andexanet alfa before starting IVT with rtPA in order to minimise the potential thromboembolic risk associated with andexanet alfa administration.

Selection of andexanet alfa dose (low or high dose) in the STROACT study will be guided by anti-Xa activity level and the time from the last intake of apixaban or rivaroxaban dose before AIS (please refer to Table 1 below).

Table 1 Dosing of andexanet alfa in the STROACT study (low and high dose).

**Anti-Xa activity level**	**Time form the last dose of apixaban or rivaroxaban**		
	**<4h or unknown**	**4-8h**	**≥8h**
>50ng/mL, <100ng/mL	High dose	Low dose	Low dose
≥100ng/mL	High dose	High dose	High dose

For detailed information on dosage and administration please refer to Section 7.1.2.

4.5 END OF STUDY DEFINITION

Study will be completed once the last treated patient completes the last study visit (End of Study Visit).

4.5.1 PREMATURE STUDY TERMINATION

The study may be terminated before the planned end of study by the principal investigator's decision if it is found that the patients participating in the study are exposed to unnecessary health risks. Such decision should be based on IDMC recommendations, and the well-being of patients must always be put first in the considerations before decision about premature study termination is taken.

5 STUDY POPULATION

Previously functionally independent patients (0-2 points on the modified Rankin scale) taking one of the oral non-vitamin K antagonist oral anticoagulants (rivaroxaban, apixaban or dabigatran) as a prevention of cardioembolic and/or thromboembolic events, who despite maintaining adequate therapeutic anti-IIa/anti-Xa activity, which is directly proportional to the plasma NOAC concentration, have an ischemic stroke in the absence of another possibility of AIS specific treatment (i.e. primary mechanical thrombectomy treatment cannot be used – no occlusion of large cerebral/intracranial artery).

Detailed inclusion and exclusion criteria are provided in Section 5.1 and 0 respectively. Patients who do not meet the eligibility criteria requirements for the STROACT study are described as screen failures; refer to Section 5.4.

Prospective approval of protocol deviations to recruitment and enrolment criteria, also known as protocol waivers or exemptions, is not permitted.

5.1 INCLUSION CRITERIA

To be eligible for enrolment patient **must meet all** the following inclusion criteria:

1. Obtaining informed consent to participate in the trial prior to randomisation.

   NOTE: Patients whose neurological deficit is severe enough to make it impossible to sign the consent form are allowed to give only their oral consent to participate in the study. However, this consent should be additionally certified by the signature of two independent witnesses (who are neither family members of the patient nor the STROACT study staff) or by the signature of his/her legal representative. **Patients with aphasia and/or other speech disorders may be included into the study if following neurological assessment of the recruiting stroke physician, they are able to understand all important information about the study.**
2. Age ≥18 years.
3. Clinical diagnosis of acute ischemic stroke (sharply defined onset of first symptoms) resulting in a disabling neurological deficit.
4. Therapy with an oral anticoagulant that is the non-vitamin K antagonist oral anticoagulant (dabigatran, apixaban, or rivaroxaban) with laboratory confirmed therapeutic anti-IIa/anti-Xa activity measured as a plasma concentration > 50 ng/mL or the corresponding blood concentration of dabigatran (in the respective arm of the study). However, in patients on dabigatran, it is possible to enrol such a subject into the study based solely on a patient's or witness' report on the last intake of the anticoagulant ie, when it was administered within the last 24 hours preceding initiation of the study treatment. In these cases it is obligatory to collect and store blood for post-hoc anti-IIa testing.
5. Administration of study intervention (intravenous thrombolysis with alteplase or placebo) should be possible to start within 4.5 hours from AIS symptoms onset or the last time the patient was seen without symptoms, as per investigator's judgment.

   NOTE: If patient had been randomised and there was an explicit clinical justification for delay in staring study intervention within 4.5h window, patient might continue in the study if rtPA (or rtPA placebo) could be administered within 6.0h from AIS onset.

   NOTE: In patients recruited to STROACT study, in addition to the inclusion / exclusion criteria, apply all standard clinical practice indications and contraindications for rtPA administration in acute ischemic stroke unless stated otherwise in this protocol.

5.2 EXCLUSION CRITERIA

To be eligible for enrolment patient **must not meet** any of the following exclusion criteria:

1. Occlusion of a large intracranial vessel in CT/MR angiography (CTA/MRA), corresponding to the current acute neurological deficit being an indication for primary mechanical thrombectomy.

   NOTE 1: Patients who qualified to the mechanical thrombectomy cannot be enrolled to the STROACT study.
2. Significant disability prior to the current stroke event defined as >2 points on the modified Rankin Scale (mRS) and/or significant impairment of the cognitive function prior to AIS (the latter documented in patient's medical records).
3. Mild and rapidly improving neurological deficit with high probability of complete recovery.
4. Clinically severe stroke with >18 points in NIHSS.
5. Neuroimaging findings that might be responsible for acute neurological deficit ("stroke mimics") and/or are contraindications for standard thrombolytic treatment: such as intracranial and/or intracerebral bleeding, tumours, abscesses and other.
6. Treatment with the following anticoagulants:
   a. Oral vitamin K antagonist (warfarin, acenocumarol),
   b. Unfractionated heparin,
   c. Low molecular weight heparin, or
   d. Inhibitors of coagulation factor Xa or IIa other than dabigatran, rivaroxaban, or apixaban
7. Whole blood, and/or blood clotting factors (such as: prothrombin complex concentrate [PCC], recombinant factor VIIa [rVIIa], fresh frozen plasma [FFP]) administered within 7 days before study treatment initiation.
8. Anti-IIa/ Xa activity (which is assumed to be directly proportional to the NOAC plasma concentration) is ≤50 ng/mL or the respective concentration of dabigatran (in the respective arm of the study).
9. CT or MRI initial lesion volume >1/2 of the anatomical perfusion area of the middle cerebral artery (MCA), or anterior cerebral artery (ACA), or posterior cerebral artery (PCA).
10. Suspected subarachnoid haemorrhage based on specific symptomatology and/or physical examination (even if CT/MRI is normal).
11. Any history of subarachnoid or intracerebral haemorrhage, so not including previous (currently normal in neuroimaging) traumatic sub-or epidural hematomas > 6 months before the current acute stroke.
12. Any past (chronic) medical illnesses that significantly impairs patient's functional status down to mRS 3 points or more (thus not only related to CNS pathologies and including cognitive impairment), and/or with a poor prognosis (e.g., neoplasms individually assessed to be of poor prognosis).
    NOTE: Patients after treatment of intracranial aneurysm may be considered for recruitment into the STROACT trial if the procedure was performed > 3 months prior to randomisation.
13. History of major surgery / trauma within 2 months before the current acute stroke.
14. History of acute ischemic stroke or any other medical condition treated with intravenous thrombolysis, or ischemic stroke treated with mechanical thrombectomy, within the 72 hours preceding the current patient's stroke symptoms.
15. Recent (within 10 preceding days) traumatic external heart massage, obstetrical delivery, lumbar puncture, any puncture of a non-compressible blood vessel.
16. Recent (within 4 preceding weeks) myocardial infarction.
17. Severe trauma at the onset of acute ischemic stroke (e.g., skull fracture, long bone fracture, pelvic fracture).

18. Expected need for major surgery within 72 hours after randomisation (e.g., laparotomy, hip femoral/pelvic fracture surgery, endarterectomy).
19. Cerebral venous sinus thrombosis (CVST)
20. Pulmonary embolism.
21. Suspected infective endocarditis and/or pericarditis.
22. Acute pancreatitis.
23. Systemic or suspected cerebral vasculitis.
24. Documented active ulcerative gastrointestinal disease during the last 3 months, documented oesophageal varices.
25. Neoplasm with increased bleeding risk.
26. Severe liver disease including acute hepatic failure, cirrhosis with/or without portal hypertension.
27. Haemorrhagic retinopathy.
28. Haemorrhagic diathesis (e.g., von Willebrand disease, haemophilia and similar inherited coagulopathies).
29. Platelet count <100,000/mm^3.
30. Active or recent severe, life-threatening bleeding.
31. Congenital or acquired coagulopathy presenting with:
    a. Prolonged aPTT above 30% of the upper limit of normal (local laboratory reference range),
    b. Increased INR ≥1.7
32. Blood glucose <50 mg/dl (2.8 mmol/l) or >400 mg/dl (22.2 mmol/l)

    NOTE: Such patients may be enrolled into the STROACT trial if CT or MRI lesion corresponds to persisting acute neurological deficit even after restoration of blood glucose down to 250 mg/dl or less.
33. Severe high blood pressure, i.e., systolic blood pressure (SBP) > 185 mmHg or diastolic blood pressure (DBP) > 110 mmHg immediately before the study treatment administration or suspected need for aggressive medication use (e.g., labetalol, urapidil) to maintain blood pressure below these values during further reversal and/or thrombolytic treatment.
34. Pregnancy.
35. Predicted life expectancy <3 months.
36. Participation in another clinical trial at the time of randomisation or planned inclusion in another clinical trial within less than 90 days of randomisation, provided that protocols of these trials interfere pathophysiologicaly or formally and administratively with the STROACT study.
37. Previous participation in the current clinical trial.
38. Advanced renal failure (eGFR <30 mL/min/1.73m^2).
39. Active infection with SARS-CoV-2 (up to 10 days from the first positive testing with any recommended assay or from the first symptoms of infection or severe “long” COVID-19 / severe Post-COVID Neurological Syndrome).

5.3 LIFESTYLE CONSIDERATIONS

No specific lifestyle restrictions are defined other than

5.4 SCREEN FAILURES

Due to the critical impact of time elapsed from the AIS onset to initiating IVT, the intensity of standard diagnostic procedures, and the possibility of changes in patient’s medical condition over time, the final confirmation (verification) of eligibility to receive the study treatment will take place just prior initiating study treatment. Therefore, screen failures are defined as patients who signed the ICF to participate in the clinical trial (enrolled patients) but were not:

- randomized, or
- did not receive study treatment

due to not meeting all the inclusion criteria or meeting any of the exclusion criteria along screening and/or diagnostic procedures required before study treatment administration.

These participants should have the reason for screen failure / study withdrawal recorded in the eCRF as “eligibility criteria not fulfilled”.

A minimal set of screen failure information is required to ensure transparent reporting of screen failure participants to meet the Consolidated Standards of Reporting Trials (CONSORT) publishing requirements and to respond to queries from regulatory authorities. Minimal information includes demography, screen failure details, eligibility criteria, and any SAEs.

The enrolment and randomisation process are described in Section 6.

6 STUDY PROCEDURES AND VISITS / PARTICIPANT RECRUITMENT PROCESS

6.1 RECRUITMENT SETTING

The STROACT study will involve patients admitted to the hospital (neurological emergency unit) under emergency conditions (suspected AIS). All patients enrolled in the clinical trial, whether allocated to sequential treatment with NOAC-specific reversal agent + rt-PA or placebo control, must be managed according to local acute stroke care protocols, in the same clinical environment. Such protocols are not specified by the CTP but must follow up-to-date clinical guidelines referring to effective stroke unit care.

All clinical centres participating in the STROACT study should have an effective stroke care unit and trained personnel to provide high quality, standardised specialty care for patients with acute stroke. It includes, but is not limited to:

- Written protocol/procedures for expedite diagnosis and treatment of patients with suspected acute stroke
- Immediate access to CT or MR brain scanning (24 hours a day)
- Immediate access to laboratory (24 hours a day)
- Specialised facility where thrombolysis may be administered, and the patient monitored according to study protocol (preferably an acute stroke unit).

As a standard procedure after admission, intravenous access, monitoring of physiological variables, correction of any abnormalities, and where clinically appropriate, intravenous fluid therapy should be initiated.

6.2 SCREENING

At screening, after a patient has been identified as a potential study participant, the investigator must:

- Obtain informed consent to participate in the study from the patient or two independent witnesses (who are neither family members of the patient nor the STROACT study staff) or patient’s legal representative before performing any study specific / study related procedures (see Section 6.3).
- Preliminarily verify patient's eligibility against inclusion / exclusion criteria (see points 3.1 and 3.2)

  NOTE: Due to critical impact of time from AIS onset to initiating IVT, intensity of standard diagnostic procedures, and possibility of patient medical changes over time, confirmation of eligibility at screening is considered “conditional” (based on results of standard diagnostic procedures and best judgment of the investigator). Final confirmation of participant’s eligibility must be verified before initiating study treatment administration.

- Perform study required procedures and examinations (procedures for Visit 0 are described in Section 6.5.1).
- Assign a unique patient number to each eligible patient using the eCRF / web-based system.

The investigator should keep a record of patients (screening log) for whom informed consent (ICF) has been obtained, and preliminary assessment of eligibility for the study has been carried out.

In case patient is classified as screen failure or withdraws his/her consent to participate in the study, his/her patient number cannot be used again.

6.3 INFORMED CONSENT AND ELIGIBILITY

6.3.1 INITIAL INFORMATION AND INITIAL APPROACH

Participants must be informed that their participation is voluntary, and they are free to refuse to participate and may withdraw their consent at any time and for any reason during the study. Participants or their legally authorised representative will be required to sign a statement of informed consent.
Informed consent form (ICF) must follow ICH guidelines and meet the requirements of applicable local regulations, and the IRB/IEC or study centre.

6.3.2 CONSENT PROCESS

The investigator or his/her representative will explain the nature of the study to the participant or his/her legal representative and answer all questions regarding the study.

Participant must be informed that participation in the clinical trial is voluntary and they are free to refuse to participate and may withdraw their consent at any time and for any reason during the study. Participants or their legally authorised representative or two independent witnesses will be required to sign a statement of informed consent that meets applicable laws and regulations.

Patients whose worsening neurological deficit makes it impossible to sign the consent form may give their oral consent to participate in the study. This consent should be additionally certified by the signature of two independent witnesses (who are neither family members of the patient nor the STROACT study staff) or patient's legal representative. **Patients with aphasia and/or speech disorders may be included in the study if in the opinion of the qualifying physician they are able to understand the idea of the study.**

Informed consent to participate in the study from the patient or his/her legal representative must be obtained before performing any study specific / study related procedures.

6.3.3 CONSENT/ASSENT AND OTHER INFORMATIONAL DOCUMENTS PROVIDED TO PARTICIPANTS

A copy of the ICF(s) must be provided to the participant or the participant's legally authorised representative.
Patients will receive information card confirming her/his participation in the STROACT clinical trial.

6.3.4 ELIGIBILITY

Due to critical impact of time from AIS onset to IVT initiation, final verification of participant's eligibility (all inclusion and exclusion criteria verified) will take place after randomisation and before initiating study treatment administration.

6.4 RANDOMISATION

Subjects allocated to A, B, or C study group (arm), defined by NOAC taken before AIS onset (see Section 4.1), will be randomized in 1:1 ratio to receive either active treatment or placebo (comparator).

A stratified variable block length randomization based on a computer-generated randomization schedule integrated into the eCRF software will be used (see Section 7.3.1).

Stratification will consider severity of the IS at randomization (<6 points in NIHSS, and ≥6 points in NIHSS).

6.4.1 TIMING OF RANDOMISATION

The magnitude of the therapeutic effect expected for intravenous thrombolytic reperfusion treatment depends on the time from the AIS symptoms onset to therapy initiation. Randomisation should be performed after receipt of laboratory results of the anti-IIA or anti-Xa activity assessment (must be >50 ng/mL) or corresponding blood concentration of dabigatran, and verification of other eligibility criteria (see Section 6.4.3). However, in patients on dabigatran, it is possible to enrol such a subject into the study based solely on a patient's or witness' report on the last intake of the anticoagulant ie, when it was administered within the last 24 hours preceding initiation of the study treatment. In these situations it is obligatory to collect and store blood for post-hoc anti-IIa testing. The procedure is described in the Laboratory Manual.

Randomisation performed within up to 3.0 hours after the onset of the first symptoms of AIS should allow to initiate IVT with alteplase (rtPA) within 4.5 h window, but no later than 6 h from the AIS onset.

6.4.2 TREATMENT ALLOCATION

Eligible patients in each study group will be allocated to receive study intervention (active treatment or placebo as a comparator) which is defined as follows:

- Active treatment: sequential intravenous administration of NOAC-specific antidote (idarucizumab or andexanet) and rtPA (alteplase) infusion, or
- Comparator treatment: sequential intravenous administration of NOAC-specific antidote placebo (infusion of 0.9% NaCl) and rtPA corresponding placebo (infusion of 0.9% NaCl).

6.4.3 RANDOMISATION PROCESS

Randomisation will be performed by the investigator (or trained designee) in the eCRF using the predefined randomisation algorithm built into the eCRF software and is available 24/7 (see Section 7.3.1).

The investigator will enter specific patient information to allow identification of the participant (date of birth) and confirm that information to perform randomisation is available (listed below).

- All eligibility criteria are met at the time of randomisation
- Patient should be able to start study treatment (i.e., thrombolysis) within 4.5h, but not later than within 6h from AIS onset
- Results of anti-IIa or anti-Xa activity/ blood concentration of dabigatran as per NOAC taken before AIS must be available before randomisation. However, in patients on dabigatran, it is possible to enrol such a subject into the study based solely on a patient's or witness' report on the last intake of the anticoagulant ie, when it was administered within the last 24 hours preceding initiation of the study treatment. In these situations it is obligatory to collect and store blood for post-hoc anti-IIa testing. The procedure is described in the Laboratory Manual.

The AIS NIHSS severity score must be provided to perform randomisation (stratification factor) and unique patient randomisation number will be generated.

Once patient have been randomised, the eCRF system will generate an automatic notification informing the unblinded personnel of the appropriate to prepare allocated study treatment accordingly.

The eCRF completion manual including detailed instructions how to perform randomisation of a patient will be provided to each study site.

6.4.4 POST-RANDOMISATION ACTIONS

After generating patient randomization number in the eCRF by the investigator, unblinded personnel will receive notification about allocated study treatment and will prepare study treatment (active or placebo) based on notification generated via eCRF.

Administration of sequential therapy should be initiated immediately after receiving masked study medications ready for use.

Administration of rtPA (second component of the study intervention) should be initiated within 4.5h time window calculated from the onset of AIS symptoms, but no later than 6h in specific circumstances (i.e. randomisation was performed correctly but unavoidable factors caused delay in administration of rtPA beyond 4.5 hours). Delay of the rtPA bolus administration beyond 6h from the AIS onset is not allowed.

Details of preparing active and placebo treatment will be included in the Pharmacy Manual provided to each study site.

6.5 STUDY VISITS AND PROCEDURES OVERVIEW

The STROACT study includes screening period, treatment administration and post-treatment follow-up.

Due to significant time constraints for appropriate standard AIS treatment initiation, or may benefit from participating in the study, informed consent should be obtained as soon as possible (see Section 6.3).

An absolute condition for carrying out any activities specifically related to the study is the prior obtaining of informed consent from the person enrolled in the study.

6.5.1 VISIT 0 (PRE-TREATMENT) – SCREENING, ENROLMENT AND RANDOMISATION

The following examinations and procedures should be performed in the patient considered for enrolment and randomisation in the study:

1. Medical history with demographic information (obtained from the patient, his family, or the Emergency Medical Team responsible for patient's transfer to the hospital), including:
   a. concomitant diseases and medications taken; it is <u>important to determine when the last dose of the oral anticoagulant was taken</u>
   b. functional status prior to the current acute stroke (based on modified Rankin Scale [mRS])
   c. the exact time of onset of symptoms of the current IS of or the time when the patient was last seen without these symptoms
2. Verification of inclusion/ exclusion criteria.
3. ECG.
   <u>NOTE 1</u>: ECG must be performed once preferably within the first 24 hours from AIS but not later than 72 hours.
4. Physical examination.
5. Vital signs (BP and HR).
6. Signs and symptoms of infection(s).
7. Neurologic examination based on National Institutes of Health Stroke Scale (NIHSS).
8. Establishment of intravenous access (if not present yet); two are preferred for enrolled patients within groups B and C i.e., those on apixaban and rivaroxaban).

9. Collection of blood sample for the following lab tests:
    a. Anti-IIa activity for dabigatran or anti-Xa for rivaroxaban or apixaban or blood concentration of dabigatran.
       NOTE 1: However, in patients on dabigatran, it is possible to enrol such a subject into the study based solely on a patient's or witness' report on the last intake of the anticoagulant ie, when it was administered within the last 24 hours preceding initiation of the study treatment. In these situations it is obligatory to collect and store blood for post-hoc anti-IIa testing. The procedure is described in the Laboratory Manual.
    b. Additional coagulation parameters: INR, APTT, fibrinogen
    c. Complete blood count (CBC) with platelet count
    d. Blood glucose concentration
    e. Biochemistry panel including electrolytes, serum creatinine (eGFR), CRP
    f. Troponin I concentration
    g. Transaminases (AlAT, AspAT) and bilirubin
    h. Albumin
    i. BNP/ NT-proBNP plasma concentration

10. Brain imaging – contrast-free CT scan (whenever possible with an assessment of the severity of early ischemic lesions preferably using the ASPECTS scale) or MRI (minimum requirements are DWI, SWI and FLAIR sequences, or equivalents).
11. DUS of extracranial / intracranial arteries.
    NOTE 1: The test should be performed at least once during hospitalization (V0-V3).
12. Echocardiography.
    NOTE 1: The test should be performed at least once during hospitalization (V0-V3).

The patient must be closely monitored, and any AEs/ Ars must be recorded in the patient's medical record and in the eCRF.

NOTE 1: If the patient presents with ≥ 6 points on the NIHSS scale, or the investigator finds a composition of symptoms suggestive of large vessel occlusion (LVO), it is necessary to expand the neuroimaging with CT or MRI angiography of carotid / vertebral / circle of Willis arteries to assess the indication for primary mechanical thrombectomy.

NOTE 2: If patient's blood pressure is exceeding > 185/110 mmHg, the blood pressure should be lowered by pharmacological means prior to randomisation and/or study treatment administration (e.g., labetalol or urapidil given in boluses and/or continuous infusion using an infusion pump. In the absence of sustained satisfactory response, i.e. inability to achieve blood pressure ≤185/110 mmHg, or suspected need for aggressive medications use (labetalol, urapidil) to maintain blood pressure below this level during further reversal and thrombolytic treatment, excludes the patient from the study (see exclusion criterion #30).

NOTE 3: The patient should be included in the study and randomized as soon as possible after the onset of stroke symptoms (time of randomisation should not exceed 3.0 h time window), so that thrombolytic treatment (rtPA) or corresponding placebo can be administered within 4.5 hours window from the onset of AIS symptoms (but no later than 6 hours in special circumstances).

NOTE 4: Most of activities / procedures required in the STROACT study protocol are included in the standard of care protocols expected to be carried out in patients admitted to the hospital with preliminary diagnosis of stroke. If any of them have already been performed as part of a routine

clinical evaluation (i.e., not specifically for the purposes of this clinical trial), there is no need to repeat them after obtaining the patient's informed consent.

6.5.2 VISIT 1 (DAY 1) – STUDY TREATMENT ADMINISTRATION

After obtaining lab results of anti-IIa activity for dabigatran (or dabigatran concentration itself) or anti-Xa for rivaroxaban or apixaban confirming high anticoagulation activity (>50 ng/ml) study treatment can be administered. However, in patients on dabigatran, it is possible to enrol such a subject into the study based solely on a patient's or witness' report on the last intake of the anticoagulant ie, when it was administered within the last 24 hours preceding initiation of the study treatment. In these situations it is obligatory to collect and store blood for post-hoc anti-IIa testing. The procedure is described in the Laboratory Manual. Blinded study treatment will be dispensed by unblinded personnel according to the treatment allocation instruction received via eCRF notification (see Section 6.4.3).

Study treatment will be administered according to the study protocol as a as sequential intravenous administration of NOAC-specific antidote (idarucizumab or andexanet) and tPA (alteplase) or corresponding placeboes for each of the study treatment components. **Please refer to dosing instructions in Section 7.1.2.**

1. Vital signs. BP and HR should be monitored and recorded:
   - in the 1. hour
   - in the 2. hour
   - in the 3. hour
   - in the 4. hour
   - in the 5. hour
   - in the 6. hour

   from the start of study treatment administration.
2. Two consecutive blood sample for anti-Xa activity for rivaroxaban or apixaban must be collected:
   - after completion of antidote (or antidote placebo) bolus injection
   - after completion of rtPA (or corresponding placebo) infusion.

   Measurements anti-IIa 2nd and 3rd are only facultative.

The start and completion time of each study treatment component, time of blood sample collection for assessment of anti-IIa/Xa activity must be recorded in patient medical records and in eCRF.

3. Review of concomitant medications.
4. DUS of extracranial / intracranial arteries (if not done before).

   NOTE 1: The test should be performed at least once during hospitalization (V0-V3).
5. Echocardiography if not done before).
   NOTE 1: The test should be performed at least once during hospitalization (V0-V3).
6. Collection of blood sample for measure of blood glucose concentration. Obligatory only in cases where the screening value was beyond that defined in inclusion/exclusion criteria.
7. ECG (if not done before).
   NOTE 1: ECG must be performed once preferably within the first 24 hours from AIS but not later than 72 hours.
8. Review of safety endpoints:
   1) any symptomatic intracranial haemorrhage (sICH),

2) any life threatening extracranial bleeding,
3) recurrent or new acute thromboembolic stroke,
4) any life threatening thromboembolic complications,
5) any significant allergic reactions assumed to be related with the administered substances (antidotes, rtPA).

Patient must be closely monitored during the study treatment administration and any AEs/Ars recorded in patient's medical records and in eCRF.

6.5.2.1 SAFETY MEASURES DURING IVT ADMINISTRATION

Patient must be closely monitored during study treatment administration for signs and symptoms of intracranial and/or extracranial bleeding and other potential complications.

One of the most dangerous complications of intravenous thrombolysis treatment is intracranial bleeding. According to SITS-MOST Register, a clinical deterioration of ≥ 4 points in NIHSS assessment of stroke severity associated with presence in neuroimaging examination of PH2 type bleeding (parenchymal haemorrhage type 2) according to ECCASS II classification is indicative of secondary symptomatic haemorrhagic transformation (symptomatic Intracranial Haemorrhage, sICH).

In the event of a suspected acute intracranial bleeding during the intravenous administration of thrombolytic treatment (i.e., when the patient experienced a severe headache, a sharp increase in blood pressure, nausea, vomiting, aggravation of an existing neurological deficit) - the infusion of rtPA should be stopped immediately and an urgent additional neuroimaging examination (head CT) should be performed.

In case of suspicion of other types of bleeding (to other areas of the body) - urgent imaging and laboratory diagnostics should be performed and applicable guidelines should be followed.
Similarly, in the event of a suspected new thromboembolic incident - urgent neuroimaging diagnostics should be performed, and applicable clinical guidelines should be followed.
For ECASS II classification of haemorrhagic transformation please refer to Section 9.1.3.3.

6.5.3 VISIT 2 (DAY 2) – POST-TREATMENT FOLLOW-UP 1

The following examinations and procedures should be performed at Visit 2:

1. Vital signs (BP and HR).
2. Signs and symptoms of infection(s).
3. Neurologic examination based on National Institutes of Health Stroke Scale (NIHSS).
4. Collecting blood sample for the following tests:
    - Coagulation parameters: INR, APTT, fibrinogen
    - Complete blood count (CBC) with platelet count
    - Blood glucose concentration
    - Biochemistry panel including electrolytes, serum creatinine (eGFR), CRP
    - Troponin I concentration
    - Transaminases (AlAT, AspAT) and bilirubin
    - Albumin.
5. Brain imaging: contrast-free CT scan (or MRI).
6. Review of concomitant medications.
7. DUS of extracranial / intracranial arteries (if not done before).

NOTE 1: The test should be performed at least once during hospitalization (V0-V3).

8. Echocardiography if not done before). NOTE 1: The test should be performed at least once during hospitalization (V0-V3).
9. Review of safety endpoints:
    1) any symptomatic intracranial haemorrhage (sICH),
    2) any life threatening extracranial bleeding,
    3) recurrent or new acute thromboembolic stroke,
    4) any life threatening thromboembolic complications,
    5) any significant allergic reactions assumed to be related with the administered substances (antidotes, rtPA).

The patient must be closely monitored, and any AEs/ Ars must be recorded in the patient's medical record and in the eCRF.

6.5.4 VISIT 3 (DAY 7) – POST-TREATMENT FOLLOW-UP 2

The following assessments / procedures shall be carried out on day 7 (± 1 day [=24 hours]) after the acute phase study treatment:

1. Vital signs (BP and HR).
2. Signs and symptoms of infection(s).
3. Performance status assessments including:
    a. patient's neurological status using NIHSS scale
    b. patient’s performance using mRS
    c. patient evaluation using Barthel scales, MoCA, IQCODE and HADS questionnaires.
4. Collecting blood sample for the following tests:
    - Coagulation parameters: INR, APTT, fibrinogen
    - Complete blood count (CBC) with platelet count
    - Biochemistry panel: CRP
    - BNP/ NT-proBNP.
5. MRI of the head/brain.
6. Review of adverse events.
7. Review of safety endpoints:
    1) any symptomatic intracranial haemorrhage (sICH),
    2) any life threatening extracranial bleeding,
    3) recurrent or new acute thromboembolic stroke,
    4) any life threatening thromboembolic complications,
    5) any significant allergic reactions assumed to be related with the administered substances (antidotes, rtPA).
8. Review of concomitant medications.
9. DUS of extracranial / intracranial arteries (if not done before).
   NOTE 1: The test should be performed at least once during hospitalization (V0-V3).
10. Echocardiography if not done before).
    NOTE 1: The test should be performed at least once during hospitalization (V0-V3).

Data should be entered on the eCRF.

6.5.5 VISIT 4 (DAY 30) – POST-TREATMENT FOLLOW-UP 3

The patient's assessment should be carried out with patient and/or caregiver. A visit to the clinic is allowed but can be conducted as a phone call.

The following assessments shall be carried out / information obtained on day 30 (±2 days) after the acute phase study treatment:

1. Patient's performance using mRS scale.
2. Patient's whereabouts (own home / family care, care home, hospital, others).
3. Review of adverse events.
4. Review of concomitant medications.
5. Review of safety endpoints:
    1) any symptomatic intracranial haemorrhage (sICH),
    2) any life threatening extracranial bleeding,
    3) recurrent or new acute thromboembolic stroke,
    4) any life threatening thromboembolic complications,
    5) any significant allergic reactions assumed to be related with the administered substances (antidotes, rtPA).

Phone call contacts must be documented in patient medical records and entered on eCRF accordingly.

6.5.6 VISIT 5 (DAY 90) – END OF STUDY

The patient's assessment should be carried out with patient and/or caregiver. A visit to the clinic is strongly preferred but in exceptional situations can be conducted as a phone call (the reasons for not visiting the center (hospital) should be given in detail).

The following assessments shall be carried out / information obtained on day 90 (±3 days) after the acute phase study treatment (End of Study Visit):

1. Vital signs (BP and HR).
2. Signs and symptoms of infection(s).
3. Performance status assessments including:
    a. Patient's neurological status with the NIHSS scale
    b. Patient's performance using mRS
    c. Patient evaluation using Barthel scales, MoCA and HADS questionnaires.
4. Review of adverse events.
5. Review of safety endpoints:
    1) any symptomatic intracranial haemorrhage (sICH),
    2) any life threatening extracranial bleeding,
    3) recurrent or new acute thromboembolic stroke,
    4) any life threatening thromboembolic complications,
    5) any significant allergic reactions assumed to be related with the administered substances (antidotes, rtPA).
6. Review of concomitant medications.

Phone call contacts must be documented in patient medical records and entered on eCRF accordingly.

6.5.7 UNSCHEDULED VISIT

Additional (unscheduled) visits may take place between visits scheduled in the protocol to assess the occurrence and effects of adverse reactions or endpoints, as well as when a patient withdraws from the study.

7 STUDY INTERVENTION

7.1 STUDY INTERVENTION(S) ADMININSTRATION

7.1.1 STUDY INTERVENTION DESCRIPTION

Patients with AIS on NOAC (dabigatran, apixaban or rivaroxaban) who are eligible for the study will receive reperfusion thrombolytic treatment with intravenous rtPA (alteplase) following administration of the specific reversal agent / antidote (idarucizumab for dabigatran; andexanet alpha for apixaban and rivaroxaban).

Study intervention is defined as sequential therapy including administration of NOAC specific neutralising (reversal) agent / antidote (idarucizumab or andexanet) and rtPA (alteplase) or corresponding placebo.

Placebo to antidote and rtPA (0.9% NaCl solution) will be prepared *ex tempore* immediately after randomisation.

All components of the study intervention (combination / sequential therapy) are commercially available medicinal products. Dosage and administration will follow the current local prescribing information.

7.1.2 DOSING AND ADMINISTRATION

All components of the study intervention (combination / sequential therapy) are commercially available medicinal products. Dosage and administration will follow the current local prescribing information.

In the STROACT study, rtPA (both bolus and infusion) should be administered to a vain of the arm contralateral to the ischemic lesion (commonly paretic), whereas antidote (idarucizumab or both bolus and infusion of andexanet alfa) to a vain of the arm ipsilateral to the ischemic lesion. For many years of the use of rtPA for ischemic stroke, there have been no major or significant reports showing any differences between action of rtPA administered to ipsi- and contra-lateral arms (that might be potentially caused by some processes like for example local autonomic disturbances), whereas data on such potential associations for both anitidotes are poor.

Table 2 Study groups and investigational treatment.

Study treatment group	Antidote and rtPA dosing and administration
Study group A<br>(patients on dabigatran)	1. Antidote<br>Idarucizumab at a dose of 5 g (2 vials of 2.5 g/50 mL) will be administered intravenously as two consecutive infusions or as a rapid intravenous injection (bolus) over 5 to 10 minutes each.<br><br>2. Reperfusion thrombolytic treatment<br>Alteplase (rtPA) administered intravenously at a dose of 0.9 mg/kg (maximum 90 mg), of which 10% of the calculated dose in a rapid intravenous injection (bolus) within 1-2 minutes, and the remainder, i.e. 90% of the calculated dose in an intravenous infusion within 1 hour (syringe pump).
Study group B<br>(patients on apixaban)	1. Antidote<br>Andexanet alpha will be administered as an intravenous bolus a:<br>• Low dose: target infusion rate of 30 mg/min for approximately 14 minutes (up to the dose of 400 mg), followed by a continuous infusion at 4 mg/min for 120 min<br>• High dose: target infusion rate of 30 mg/min for approximately 27 minutes (up to the dose of 800 mg), followed by a continuous infusion at 8 mg/min for 120 min<br>(administration in a syringe pump after reconstitution of all required vials).<br><br>2. Reperfusion thrombolytic treatment<br>Alteplase (rtPA) - the same dosing and administration instructions as in study group A.
Study group C<br>(patients on rivaroxaban)	1. Antidote<br>Andexanet alpha - the same dosing and administration instructions as for the study group B.<br><br>2. Reperfusion thrombolytic treatment<br>Alteplase (rtPA) - the same dosing and administration instructions as for the study group A, and B.

7.2 TRIAL MEDICINAL PRODUCT PREPARATION/HANDLING/STORAGE/ACCOUNTABILITY

7.2.1 MANUFACTURES, AND OTHER PARTICULARS OF MEDICINAL PRODUCTS

All medications used as study intervention are commercially available products.

Hospital pharmacy of each study centre is responsible for acquisition, handling, storage, and accountability of medications used as study intervention unless the local logistics of the hospital pharmacotherapies is differently structured. Detailed instructions specific for the STROACT study will be provided in the Hospital Manual to each participating site.

Tested Product	Pharmaceutical Form and Content	Trade name /Manufacturer
Idarucizumab	Solution for injection containing 2.5 g of idarucizumab in 50 mL glass vial.	Praxbind<br>Boehringer Ingelheim
Andexanet alpha	Powder for preparing solution for infusion. Each vial contains 200mg of andexanet alpha, after reconstitution the concentration is 10mg/ml.	Ondexxya<br>Portola Netherlands B.V.

Alteplase	Powder in vials for preparing alteplase solution. Vials may contain 10, 20 or 50mg of alteplase (rtPA). Vials are supplied with solvent for preparing solution for injection and infusion.	Actilyse<br>Boehringer Ingelheim

For more details, please refer to the respective SmPC and/or local prescribing information provided by the manufacturer with commercial supplies.

Placebo control will be prepared from commercial supplies of 0.9% NaCl as per local suppliers to each study centre.

Hospital pharmacy of each study centre is responsible for the medicinal products (study treatment components) acquisition.

7.2.2 PRODUCT STORAGE AND ACCOUNTABILITY

All products should be stored in a safe place under appropriate conditions according to the manufacturer instructions (Please refer to respective SmPC and/or approved local prescribing information).

Hospital pharmacy of each study centre is responsible for medicinal products storage and accountability unless the local logistics of the hospital pharmacotherapies is structured otherwise. Detailed instructions specific for the STROACT study will be provided in the Hospital Manual to each participating site.

7.2.3 PREPARATION/DISPENSING

After patient has been randomized via eCRF based randomization system the information about allocation will be automatically generated and released to the unblinded personnel responsible for study treatment preparation and dispensing.
For technical details of each component of the study intervention preparation for administering to the participant please refer to the Hospital Pharmacy Manual.

7.3 MEASURES TO MINIMIZE BIAS: RANDOMIZATION AND BLINDING

7.3.1 RANDOMISATION

Randomization procedure is to be performed to minimize bias in the assignment of subjects to treatment groups. Subjects in each study group (A, B, C - based on NOAC taken before AIS event; see Section 4.1) will be randomized in 1:1 ratio to either placebo or active treatment. A stratified variable block length randomization based on a computer-generated randomization schedule integrated in the eCRF software will be used. Stratification will consider severity of the IS at randomization (<6 points in NIHSS, and >6 points in NIHSS).

7.3.2 BLINDING

This study is designed as double-blind clinical trial. Once the patient has been finally qualified to receive study treatment the unblinded personnel will prepare both components of the sequential treatment. The treating physician will receive ready to use syringes for administration to the patient, which will look the same for the active treatment and placebo.

Labelling of syringes will allow for determination of antidote and IVT components to reassure correct sequential intravenous administration while maintaining double blind of study treatment.

For further details please refer to Hospital Pharmacy Manual.

7.3.3 UNBLINDING / UNBLINDING PROCEDURES

Unblinding of the study should be avoided in order scientific integrity of the study. IDMC will evaluate on the ongoing basis the data collected during the study to assess patient safety, treatment effectiveness, potential risk-benefit ratio and ensure the validity and integrity of the study and will have access to the unblinded data.

An accidental unblinding, if occurs, should be reported to the site PI, and Lead PI, as soon as it has been identified.

The randomised allocation to the study treatment (active treatment or placebo) should be unblinded only in health-related emergency occurs, and when the knowledge of patient's treatment allocation is necessary for the implementation of appropriate diagnostic and/or therapeutic procedures.

Individual randomisation codes indicating the allocation to the treatment group of each randomised patient will be available to the investigator through the eCRF. The detailed procedure for unblinding will be described in the eCRF user manual provided to each of the participating centres.

In the case unblinding (requested or accidental), the investigator/PI shall prepare a report describing its cause and course and send it to the study coordinating centre/LPI.

7.4 STUDY INTERVENTION COMPLIANCE

The study intervention will be administered in hospitalized patients and supervised by qualified and trained personnel, therefore assumed patient compliance is 100%.

The calculated and administered doses of the study intervention (sequential administration of NOAC-antidote and rtPA) as well as timing and duration of their administration will be recorded in patient's medical records and eCRF. Any variations from the CTP will be reviewed and reported for further analyses.

7.5 THE EXPECTED DURATION OF SUBJECT PARTICIPATION, DESCRIPTION OF ALL TRIAL PERIODS INCLUDING FOLLOW-UP, IF ANY

Clinical trial timelines

First patient to be enrolled: 4Q2021

Last patients to be enrolled: 1Q2025

End of study (last patient visit): 2Q2025

The enrolment of new patients will be terminated when the minimum number of patients assumed in the sample size analysis for the primary endpoint to be achieved.

Study duration for the participant

The expected duration of the study for participants who received study treatment is up to 90 (+/- 3) days from being enrolled in the STROACT clinical trial (receiving rtPA bolus).

7.6 CONCOMITANT THERAPY

Concomitant therapy can be defined as any medical procedure or medication other than the study intervention, or standard procedure in diagnosis or management medical condition under study. Concomitant medications are defined as any medication or vaccine, including over the counter or prescription medicines, vitamins, and/or herbal supplements, that the participant is receiving at the time of enrolment or receives during the study.

Prior concomitant therapies and any new concomitant therapies that might significantly modify hemostatic parameters e.g. heparines, antiplatelet agents, anticoagulants, drugs interfering with anticoagulants if the latter are administered, introduced during study duration should be recorded in patient's medical records and entered on the eCRF. The following information must be recorded in patient's medical records and eCRF:

- Reason for use
- Dates of administration including start and end dates
- Dosage information including dose and frequency

There are no protocol specific restrictions on concomitant therapies / medications, other than specified in exclusion criteria (see Section 5.2).

7.6.1 POST-TREATMENT THROMBOEMBOLISM PROPHILAXIS

The risk of venous thromboembolism (VTE) is elevated in the first one to three months after stroke, due in part to stroke-related immobility.

Moreover, patients being previously treated with dabigatran have underlying disease that predispose them to thromboembolic events. Reversing NOAC therapy exposes patients to the thrombotic risk of their underlying disease. To reduce this risk, resumption of anticoagulant therapy / implementation of thromboembolism prophylaxis after AIS should be considered as soon as medically appropriate.

7.6.2 RESCUE MEDICINE

Alteplase related intracranial haemorrhage

Intracranial haemorrhage should be suspected if any of the following occur during the infusion of the sequential therapy or within 24 hours of randomisation:

- neurological deterioration.
- new headache.
- new acute hypertension.
- new nausea or vomiting.
- sudden decrease in conscious level or acute psychosis.

If any of these events occur, any rt-PA infusion should be stopped, and the patient examined for possible reasons for the deterioration. Blood should be taken to measure prothrombin time (PT), activated partial thromboplastin time (APPT), fibrinogen, and full blood count. CT scanning must be performed immediately, irrespective of the allocated treatment. If CT scanning confirms intracranial haemorrhage, rt-PA must not be restarted. Management should follow local protocols and will usually require consultation with a haematologist and a neurosurgeon. For patients who have received rt-PA there is no reliable evidence available to recommend any one treatment strategy over another, but the infusion of fresh frozen plasma or fresh blood is recommended and if necessary, synthetic antifibrinolytics may be administered (please refer to Actilyse SmPC).

8 STUDY INTERVENTION DISCONTINUATION AND PARTICIPANT DISCONTINUATION/WITHDRAWAL

8.1 DISCONTINUATION / INTERRUPTION OF STUDY INTERVENTION

It may be necessary for a participant to permanently discontinue study intervention. If study intervention is permanently discontinued, the participant will remain in the study to be evaluated for efficacy and safety parameters as appropriate.

Study treatment may be permanently discontinued because of:

- Informed decision of the patient
- The investigator's decision resulting from:
  - Finding of incorrect randomisation of the patient, due to which there is a risk to the health or life of the patient (non-compliance with the criteria for inclusion / exclusion of the patient in the study).
  - Adverse reactions that the investigator believes may lead to a deterioration in the patient's health (irrespective whether related or not to the treatment used).

    Temporary suspension of study treatment administration should occur if during the second phase of treatment (rtPA or placebo) if systolic blood pressure rises above 185 mmHg and/or diastolic blood pressure above 110 mmHg.

If the blood pressure cannot be lowered below 185/110 mmHg within 15 minutes, then the study treatment should be permanently discontinued.

NOTE: Premature termination of the study treatment for any reason does not mean termination of the patient's participation in the study - the patient should be further evaluated according to the study protocol (at day 2, 7, 30 and 90).

8.2 PARTICIPANT DISCONTINUATION/WITHDRAWAL FROM THE STUDY

The patient (or her/his legally authorised representative) has the right to withdraw consent to participate in the study at any time. Discontinuation of acute phase therapy (intravenous thrombolysis / placebo) does not mean automatic termination of participation in the study.

If a participant (or her/his legally authorised representative) withdraws consent, they will be specifically asked if they are withdrawing consent to all further participation in the study (treatment, assessments and follow-up) or withdrawing from treatment/assessments only and will allow further follow-up.

8.3 LOST TO FOLLOW-UP

A study participant will be considered lost to follow-up if he or she repeatedly fails to return for scheduled visits and no contact has been established with such patient or her/his legal representative or relatives by the time the study is completed (see Section 4.5), such that there is insufficient information to determine the participant’s status at that time. Before a participant is deemed lost to follow-up, the investigator or designee must make every effort to regain contact with the participant or his legal representative / relatives (where possible, 3 telephone calls and, if necessary, a certified letter to the participant’s last known mailing address or local equivalent methods). These contact attempts should be documented in the participant’s medical record.

9 STUDY ASSESSMENTS AND PROCEDURES

9.1 EFFICACY ASSESSMENTS

9.1.1 NATIONAL INSTITUTES OF HEALTH STROKE SCALE (NIHSS)

The National Institutes of Health Stroke Scale (NIHSS) is a systematic assessment tool that provides a quantitative measure of stroke-related neurologic deficit, and is valid for predicting lesion size and can serve as a measure of stroke severity; the NIHSS has been shown to be a predictor of both short and long term outcome of stroke patient.

The NIHSS will be used as a clinical stroke assessment tool to evaluate and document neurological status in acute stroke patients in the STROACT study. Stroke severity according to NIHSS scores will be stratified as follows:

- Very Severe: >25
- Severe: 15 – 24
- Mild to Moderately Severe: 6 – 14
- Mild: 1 – 5

NIHSS score (<6 points in NIHSS, and ≥6 points in NIHSS) will be the stratification factor used in randomisation.
NIHSS assessment form is included as Appendix in Section 14.1.1.

9.1.2 MODIFIED RANKIN SCALE

Modified Rankin Scale (mRS) is a functional assessment scale that measures the degree of disability or dependence of people who have suffered a stroke.

Score	Patient performance status description
0 points	no symptoms
1 point	no significant disability: able to carry out all usual activities, despite some symptoms
2 points	low degree (slight) disability: able to look after own affairs without assistance, but unable to carry out all previous activities (symptoms slightly change the current way of life and do not limit the independent functioning)
3 points	moderate disability: needs some help, but able to walk unassisted (symptoms significantly change the current way of life and prevent completely independent functioning)
4 points	moderately severe disability: unable to attend to own bodily needs without assistance, and unable to walk unassisted (symptoms definitely prevent independent life, but there is no need for constant care and assistance of the other person)
5 points	severe disability: needs constant nursing care and attention, bedridden, incontinent (patient is completely dependent on the caregiving support system)
6 points	dead

The mRS Assessment Form (Polish version) is provided in Appendix in Section 14.1.2.

9.1.3 RADIOLOGICAL EXAMINATIONS

Brain imaging and neurovascular imaging in selected patients are required in diagnostic process. All enrolled patients MUST have a pre-randomisation brain scan to exclude intracranial haemorrhage and stroke mimics. Although CT scanning is preferred, MR brain imaging is allowed provided there is sufficient radiological support in the hospital to interpret the scans. However, the MRI protocol must include the following sequences: T2 or FLAIR to exclude stroke mimics, T2* or SWI to exclude ICH, Diffusion Weighted Imaging (DWI) to identify areas of diffusion restriction that might correspond to recent ischemic pathologies (cytotoxic oedemas of territorial or subterritorial anatomic characteristics).

9.1.3.1 CT SCAN

CT scans should cover the entire brain from the foramen magnum to the vertex with 4 – 5mm thick slices through the posterior fossa and 8 – 10mm thick for the cerebral hemispheres, with no slice gap. Scans should be windowed on a width of 80 Hounsfield Units (HU) and a centre level of 35 – 40 HU. This is particularly important if scans are to be sent as printed film. All patients (irrespective of treatment allocation) MUST have a follow-up scan at 24-48 hours.

In addition, a repeat scan is required if the patient deteriorates neurologically, or intracranial haemorrhage is suspected for any reason.

9.1.3.2 MRI SCAN

Although CT scanning is preferred, MR brain imaging is allowed provided there is sufficient radiological support in the hospital to interpret the scans and a gradient echo (T_2*) is included to exclude haemorrhage (haemorrhage can be overlooked on several other types of MR imaging sequence) and Diffusion Weighted Imaging (DWI) is required to identify the recent infarct.

9.1.3.3 ECASS II CLASSIFICATION OF SECONADRY INTRACRANIAL BLEEDING

Haemorrhagic transformation may be composed of two different processes (haemorrhagic infraction - petechial haemorrhages, and parenchymal haematoma). European Cooperative Acute Stroke Study (ECASS) divides haemorrhagic transformation into four subtypes.

Table 3 ECASS classification (haemorrhagic transformation within 36 hours of AIS onset).

Category	Description
HI1	Haemorrhagic infarction type 1: - petechial haemorrhages at the infarct margins
HI2	Haemorrhagic infarction type 2: petechial haemorrhages throughout the infarct; no mass-effect attributable to the haemorrhages
PH1	parenchymal hematoma type 1: ≤30% of the infarcted area; minor mass effect attributable to the haematoma (compression of adjacent structures)
PH2	parenchymal hematoma type 2: >30% of infarct zone; substantial mass effect attributable to the haematoma (significant compression of adjacent structures)

9.1.4 ADDITIONAL FUNCTIONAL ASSESSMENTS

9.1.4.1 BARTHEL INDEX

The Barthel Index of Activities of Daily Living (ADLs) is a rating scale for the measurement of activity limitations in patients with neuromuscular and musculoskeletal conditions in an inpatient and

outpatient rehabilitation setting. It has been used with rehabilitation patients to predict length of stay and to indicate the amount of nursing care needed.

The BI consists of 10 items that measure a person's daily functioning to assess functional independence in the domains of personal care and mobility. The items include feeding, transfers from bed to wheelchair and to and from a toilet, grooming, walking on a level surface, going up and down stairs, dressing, continence of bowels and bladder. The Barthel Index (BI) ranges from 0 (most disability) to 100 (no disability).

The BI assessment will be performed at Day 7 +/- 1 day (Visit 3) and Day 90 +/- 3 days (Visit 5), to determine an early post-acute phase performance status and at the end of the observation period (Day 90 +/- 3 days, EOS).

BI Assessment Form (Polish version) is provided in Appendix in Section 14.1.3.

9.1.4.2 MONTRAL COGNITIVE ASSESSMENT (MOCA)

Cognitive function will be assessed using Montreal Cognitive Assessment – a tool designed to assess attention, executive functioning, language, memory, and orientation. This test will be performed on Day 7 +/- 1 day following application of the investigational treatment.

MoCA Assessment Form (Polish version) is provided in Appendix in Section 14.1.4.

9.1.4.3 HOSPITAL ANXIETY AND DEPRESSION SCALE (HADS)

Mood disorders are commonly seen in patients with cerebrovascular disease including stroke survivors and are associated with increased morbidity and mortality. Hospital Anxiety and Depression Scale (HADS) assessment will be used. HADS scale consists of two subscales with 7 questions each using for point scale (where 0 points indicates lowest and 21 highest possible level of anxiety or depression).

HADS assessment will be performed at Day 7 +/- 1 day (Visit 3) and Day 90 +/- 3 days (Visit 5), to determine an early post-acute phase level of depression and anxiety symptoms and at the end of the observation period (Day 90 +/- 3 days, EOS).

HADS Assessment Form (Polish version) is provided in Appendix in Section 14.1.5.

9.1.4.4 IQCODE SCALE

Informant Questionnaire on Cognitive Decline in the Elderly (IQCODE) is a tool used to assess cognitive impairment in older people. The IQCODE should be used to supplement the other patient administered tools (e.g. Barthel Index); to increase sensitivity and specificity or used in situations where the patient is unable to complete the assessment. IQCODE assessment will be performed at Day 7 +/- 1 day (Visit 3).

IQC Assessment Form (Polish version) is provided in Appendix in Section 14.1.6.

9.1.5 NATRIURETIC PEPTIDE

Brain natriuretic peptide (BNP), also known as B-type natriuretic peptide or ventricular natriuretic peptide is a hormone secreted by cardiomyocytes in the heart ventricles in response to stretching caused by increased ventricular blood volume. It is postulated that BNP can serve as a prognostic biomarker of AIS (Refs.)

B-type natriuretic peptide (BNP) and N-terminal pro-BNP (NT-proBNP) assist in the diagnosis of heart failure (HF). The difference between BNP and NT-proBNP is that one is biologically active as a hormone - BNP and NT-proBNP is biologically inactive. BNP has a much shorter half-life, thus

NT-proBNP, as a consequence of longer half-life, circulates in higher concentrations in the bloodstream.

The diagnostic sensitivity and specificity of BNP and NT-proBNP is comparable; both provide prognostic information in patients with heart failure, coronary artery disease, and valvular heart disease.

Change in BNP or NT-proBNP concentration from baseline (Visit 0) till Day 7 +/- 1 day (Visit 3) will be an exploratory endpoint, and the prognostic value of BNP or NT-proBNP concentration during AIS course and correlation with actual treatment will be evaluated.

9.2 SAFETY ASSESSMENTS

Planned time points for all safety assessments are provided in the SoA.

Safety points will be assessed during each post-treatment visit (V2 - V5) and are defined in the respective sections of the protocol (descrption of standard visit procedures).

9.2.1 EVENTS OF SPECIAL INTEREST

Events of Special Interest (ESI) are those events thought to be associated, or potentially associated with the investigational treatment and/or AIS course. In the STROACT study there are prespecified AEs / events which are considered as ESI. These events will be monitored during the study and will be analysed as the critical safety endpoints comparing the study treatment and placebo groups.

1. Mortality recorded on the day of visit 3, 4, 5 (equal to 7-, 30- and 90 days after AIS)
2. Cerebrovascular events at 7-, 30- and 90-days post AIS:
   - Recurrent ischaemic stroke
   - Neurological deterioration (NIHSS).
3. Incidence of intracranial bleeding (symptomatic and asymptomatic) assessed with CT/MRI and assessed according to European Cooperative Acute Stroke Study (ECASS II) classification at 24h +/- 4 hours and 7 days +/- 1 day after study treatment administration (please see Section 9.1.3.3).
4. Incidence and severity of major extracranial haemorrhage defined as:
   - fatal
   - severe enough to require transfusion or operation
   - an absolute decrease in haemoglobin >5 g/dL
   - a decrease in haematocrit of >15%
   - bleeding associated with persistent or serious disability).
5. Post-treatment thromboembolic and cardiovascular events.

9.2.1.1 INTRACRANIAL HAEMORRHAGES

In the Safe Implementation of Thrombolysis in Stroke-Monitoring Study (SITS-MOST) symptomatic Intracranial Haemorrhage (sICH) is defined as the deterioration of stroke severity in NIHSS of ≥ 4 points with parenchymal haemorrhage type 2 (PH2) in neuroimaging (see Section 9.1.3.3).

9.2.2 PHYSICAL EXAMINATION

The physical examination will be performed at timelines as specified in the SoA (Section 1.3) and will include an assessment of the following: general appearance, respiratory, cardiovascular, abdomen skin, head, and neck (including ears, eyes, nose, and throat), lymph nodes, thyroid, musculoskeletal (including spine and extremities), urogenital, dermatological, gastrointestinal, endocrine, hematologic/lymphatic, and neurological systems.

Targeted physical examinations are to be used by the investigator based on clinical observations and symptomatology.

Physical examination will be performed at timelines as specified in the SoA (Section 1.3); investigators should pay special attention to clinical signs related to previous serious illnesses, new or worsening abnormalities may qualify as AEs, see Section 9.3.1 for details.

9.2.3 VITAL SIGNS

Vital signs will be performed at timelines as specified in the SoA (Section 1.3). Body temperature, heart rate, respiratory rate, and blood pressure will be assessed.

9.2.4 ELECTROCARDIOGRAMS

Single 12-lead ECGs will be performed at timelines as specified in the SoA (Section 1.3) after the participant has been resting in supine position for at least 3 minutes and recorded while the participant remains in that position using an ECG machine that automatically calculates the heart rate and measures PR, QRS, QT, and QT interval corrected by Fridericia's formula (QTcF) intervals.

9.2.5 CLINICAL SAFETY LABORATORY ASSESSMENTS

Table 4 Clinical laboratory assessments.

Haematology/Haemostasis (whole blood)	Clinical Chemistry (serum or plasma)
Haematology: CBC (including platelets count)	Electrolytes (Na, K, Cl)<br>Blood glucose<br>Kidney function: creatinine (eGFR)<br>Liver tests: AsPAT, AlAT, bilirubin, albumin<br>Inflammation: CRP<br>Other: Troponin I
Haemostasis:<br>• INR, aPTT, fibrinogen<br>• Anti-IIa activity/ dabigatran concentration for dabigatran[a] or anti-Xa for rivaroxaban and apixaban	Other: Plasma BNP/ NT-proBNP

[a] In patients on dabigatran, it is possible to enroll such a subject into the study based solely on a patient's or witness' report on the last intake of the anticoagulant ie, when it was administered within the last 24 hours preceding initiation of the study treatment. In these situations, it is obligatory to collect and store blood for post-hoc anti-IIa testing. The procedure is described in the Laboratory Manual.

The investigator should assess the available results regarding clinically relevant abnormalities. Any clinically significant abnormal laboratory values should be repeated as clinically indicated and recorded on the eCRF. Situations in which laboratory safety results should be reported as AEs are described in Section 9.3.1.

9.2.6 OTHER SAFETY ASSESSMENTS

All patients enrolled in the clinical trial, must be managed according to local acute stroke care protocols, in the same clinical environment; other safety assessments not specified in this CTP are allowed as per local protocols and investigator's judgment.

9.3 ADVERSE EVENTS AND SERIOUS ADVERSE EVENTS

The principal investigator is responsible for ensuring that all staff involved in the study are familiar with the content of this section and applicable appendices. All staff and clinicians in contact with patients participating in the STROACT study are responsible for detecting, documenting, recording, and reporting events that meet the definition of an AE/SAE.

Adverse Event (AE) definition

An adverse event (AE) is defined as the development of any untoward medical occurrence in a patient / study participant administered a medicinal product / intervention and which does not necessarily have a causal relationship with this treatment. An AE can therefore be any unfavourable and unintended sign (for example an abnormal laboratory result) and symptom (for example nausea, pain), or disease temporally associated with the use of a medicinal (investigational) product / intervention, whether related or not related to the medicinal (investigational) product / intervention.

For additional information please refer to Section 14.1.1.

Serious Adverse Event (SAE) definition

A serious adverse event (SAE) is defined as an AE occurring during the study that fulfils one or more of the following criteria:

- Results in death
- Is (immediately) life-threatening,
- Requires in-patient hospitalization or prolongation of existing hospitalization,
- Results in persistent or significant disability/incapacity, or
- Is a congenital anomaly or birth defect
- Is an important medical event that may jeopardise the patient or may require medical treatment to prevent one of the outcomes listed above

For additional information please refer to Section 14.1.2.

9.3.1 DETECTING, ASSESSING AND MONITORING ADVERSE EVENTS AND SERIOUS ADVERSE EVENTS

It is the responsibility of the PI at each participating study centre, to evaluate each AE for seriousness, causality, severity, and expectedness.

Investigators should refer to the locally approved prescribing information and Summary of Product Characteristics (SmPC) of NOAC-specific antidote (idarucizumab and andexanet alpha), and alteplase (rtPA). Review of the safety information available in each SmPC and in the literature is provided in the STROACT Investigator's Brochure (IB).

Caution should be paid not to introduce bias when detecting AEs and/or SAEs. Open-ended and non-leading verbal questioning of the patient (or caregiver, or legal representative) is the preferred method to inquire about AEs.

After the initial AE/SAE report, the investigator is required to proactively follow each participant at subsequent visits/contacts. All SAEs/non-serious AEs/AEs of special interest (as defined in Sections 14.1.1, 14.1.2, 14.1.3), should be followed until resolution, stabilisation, the event is otherwise explained, or till the end of study, or the patient is lost to follow-up.

Any AE/SAE/abnormal laboratory findings that are ongoing at the time of study treatment discontinuation or any new treatment related events within 30 days of study treatment, must be followed up to resolution or until the event becomes stable (or returns to baseline) or is unlikely to

resolve further in the opinion of the investigator.

9.3.1.1 SERIOUSNESS AND SEVERITTY ASSESSMENT

Severity and seriousness of the AE will be assessed by the PI or designee. Severity is not synonymous with seriousness.

Severity assessment

The National Cancer Institute CTCAE latest version (CTCAE version 5.03) will be used for assessing AE intensity (severity of event) for all events with an assigned CTCAE grading. For those events without assigned CTCAE grades, the recommendation in the CTCAE criteria on converting mild, moderate, and severe events into CTCAE grades should be used.

A copy of the CTCAE can be downloaded from the website http://ctep.cancer.gov.

Seriousness assessment

To categorise an AE as serious it must meet one or more of the criteria:

- Results in death
- Is (immediately) life-threatening,
- Requires in-patient hospitalization or prolongation of existing hospitalization,
- Results in persistent or significant disability/incapacity, or
- Is a congenital anomaly or birth defect
- Is an important medical event that may jeopardise the patient or may require medical treatment to prevent one of the outcomes listed above

For additional explanations of the above criteria please refer to Appendix Section 14.1.2.

9.3.1.2 CAUSALITY ASSESSMENT

A guide for the assessment and interpretation of the causality question is provided in Appendix Section 14.1.6.1.

In general causality rating against the study intervention(s) in the STROACT study will be defined as “unlikely”, “possible”, “probable”, or “definite”.

9.3.1.3 ADVERSE EVENT VARIABLES

The following variables will be collected for each AE:

- AE (verbatim).
- The date and time when the AE started and stopped.
- CTCAE grade/maximum intensity or CTCAE grade/changes in CTCAE grade/intensity.
- Whether the AE is serious or not (Section 14.1.1 and Section 14.1.2).
- Investigator causality rating against the study intervention(s) (unlikely”, “possible”, “probable”, or “definite”).
- Action taken with regard to study intervention(s).
- AE caused participant’s withdrawal from study (yes or no).
- Administration of treatment for the AE.
- Outcome.

In addition, the following variables will be collected for SAEs:

- Date AE met criteria for SAE.

- Date investigator became aware of SAE.
- Seriousness criteria.
- Date of hospitalisation.
- Date of discharge.
- Probable cause of death.
- Date of death.
- Autopsy performed.
- Causality assessment in relation to study procedure(s).
- Causality assessment to other medication.

The grading scales defined in NCI CTCAE will be used for all events with an assigned CTCAE grading. For those events without assigned CTCAE grades, the recommendation in the CTCAE criteria that converts mild, moderate, and severe events into CTCAE grades should be used. (CTCAE can be assessed and/or downloaded from the Cancer Therapy Evaluation Program website http://ctep.cancer.gov).

9.3.1.4 EXPECTEDNESS

Expectedness assessment is deciding whether the adverse event presented is listed (expected) or not listed (unexpected) in the appropriate section of the SmPC.

Suspected Unexpected Serious Adverse Reactions must be reported in an expedite manner to regulatory authorities, and also assessed by the IDMC.

For definition of Suspected Unexpected Adverse Drug Reaction please refer to Appendix Section 14.1.5

9.3.2 ADVERSE EVENT REPORTING

Adverse events will be reported by the participant (or, when appropriate, by a caregiver, or the participant's legally authorised representative). The term AE includes both serious and non-serious AEs and can include a deterioration of a pre-existing medical occurrence. An AE may occur at any time, including pre-treatment phase, when no study intervention has been administered. The definitions of an AE or SAE are provided in Section 14.1.

In the STROACT study AEs and SAEs will be collected from the time of signature of the ICF, throughout the treatment period and until end of study.

All reported AEs must be entered into eCRF.

9.3.3 SERIOUS ADVERSE EVENT REPORTING

All SAEs must be reported whether or not considered causally related to the study intervention, or to the study procedure(s). All SAEs will be recorded in the eCRF.

If any SAE occurs in the course of the study, then investigators or other site personnel inform the CRO (50Bio) representatives within one day i.e. immediately, but no later than 24 hours of when she or he becomes aware of it.

Expectedness of SAE has to be determined as soon as possible (see Section 9.3.1.4 and 14.1.5)

9.4 UNANTICIPATED PROBLEMS

9.4.1 SARS-COV-2 PANDEMIC

The investigator must carry out a risk assessment for each participant and the results should be included in the patient's medical records. The investigator should keep the participant and/or legal representatives informed of the impact of the coronavirus pandemic on the participation in the study.

If deemed appropriate, investigators in conjunction with statutory study governance bodies (EC, SC and IDMC) may initiate changes to the study conduct and/or protocol if they consider that the situation requires implementation of urgent safety measures.

In such situation both participants, and Ethics Committees / regulatory authorities should be notified by the Sponsor as soon as possible.

10 STUDY MANAGEMENT AND ADMINISTARTION

10.1 CLINICAL MONITORING OF THE STUDY

CRO (50Bio) will be delegated with responsibility for clinical monitoring of the study according to CRO's Standard Operating Procedures.

Please refer also to Section 10.4 (Data Quality Assurance).

10.2 CLINICAL DATA COLLECTION AND DATA MAGEMENT

Patient's clinical data specified by the CTP will be collected via web-based eCRF. The eCRF will be designed, set up by Biostat Ltd, according to CRO's SOPs.

Data review and management process (including generation and resolution of queries) will be described in the Data Management Plan.

10.3 CLINICAL TRIAL OVERSIGHT

10.3.1 EXECUTIVE COMMITTEE

The Executive Committee (EC) is composed of Sponsor's representatives/ co-ordinating centre investigators and includes:
Prof. Bartosz Karaszewki (Chair, Lead Principal Investigator)
Dr. Dariusz Gąsecki
Dr. Sebastian Szczyrba
Dr. Bartosz Jabłoński
Dr. Adam Wyszomirski

The EC will be responsible for designing, interpreting, supervising and presenting the study results at international congresses and publishing it in medical journals, including any corrections and amendments to the study protocol.

10.3.2 STEERING COMMITTEE

The Steering Committee (SC) will be composed of Principal Investigators from all participating centres where the study will be conducted and will be supervised by the EC.

SC members will be responsible for the implementation of the STROACT clinical trial procedures and the conduct of the study in their centres.

10.3.3 INDEPENDENT DATA MONITORING COMMITTEE

An Independent Data Monitoring Committee (IDMC) will be appointed before study start, and will be responsible for ensuring patient safety, by estimating the benefit-risk balance during the study, for the overall conduct of the study and for reporting to the study EC.

The IDMC will analyse periodically and on an ongoing basis incoming safety data (i.e., safety endpoints, serious adverse events, premature withdrawals from the study due to the adverse event), and efficacy data both overall and for individual centres.

In the event of safety concerns which would require a change in the study conduct, the DMC is obliged to report this incident to the study EC.

Details of IDMC responsibilities and operating procedures will be provided in IDMC Charter.

10.4 DATA QUALITY ASSURANCE

Proper study conduct oversight at individual site, including quality assurance and control is the ultimate responsibility of each centre PI. In addition, the study / country level oversight will be within specified responsibilities for EC, SC and IDMC (see Section 10.3).

The Principal Investigator at each centre will ensure that appropriate training relevant to the study is given to study team, and that any new information relevant to the performance of this study is forwarded to the staff involved. The Principal Investigator will maintain a record of all study team members involved in the study (medical, nursing, and other staff).

Sponsor will delegate the following responsibilities to CROs (third parties):

- Clinical monitoring, i.e., CRO study monitors will perform ongoing source data verification to confirm that data entered into the eCRF by authorised site personnel are accurate, complete, and verifiable from source documents; that the safety and rights of participants are being protected; and that the study is being conducted in accordance with the currently approved study protocol, laws, and regulations.
- Regulatory management and reporting (i.e., submission of regulatory documents, applications, amendments and notifications to IRB/EC and other Competent Authorities; regulatory safety reporting – SAE, SUSAR etc)
- Data management of this study including quality checking of the data

All participant data relating to the STROACT study will be recorded on the eCRF. The investigator is responsible for verifying that data entries are accurate and correct by electronically signing the eCRF.

The investigator must maintain accurate documentation (source data) that supports the information entered in the eCRF.

During the study conduct, a CRO representative (as Sponsor designee) will conduct periodic monitoring visits to the Investigational site. Investigator and respective institution will allow 50Bio CRO monitors direct access to source documents to perform this verification.

The sponsor assumes accountability for actions delegated to other individuals (eg, Contract Research Organisations).

11 STATISTICAL CONSIDERATIONS

Statistical analyses will follow the principles of the Guidelines ICH Topic E3 and ICH Topic E9 as well as Biostat's SOPs.

11.1 GENERAL CONSIDERATIONS

11.1.1 RESPONSIBILITIES

Statistical Analysis Plan and all statistical analyses will be performed by Biostat Sp. z o. o.

11.1.2 STATISTICAL ANALYSIS PLAN (SAP)

Detailed description of study populations / subgroups, including rules for inclusion and exclusion to a given population, data analysis and results presentation will be provided in a statistical analysis plan (SAP), a separate document provided by the Biostat Sp. z o. o. prior to final database lock.

11.1.3 STATISTICAL METHODS

This section describes the statistical analyses as foreseen at the time of planning the study. Any known deviations from the planned analyses, the reason for such deviations and all alternative / additional statistical analyses that may be performed as well as the final statistical analysis will be described in the final Statistical Analysis Plan (SAP) before completion of data collection. All later deviations and / or alterations will be summarised in the Clinical Study Report (CSR).

All data collected will be analysed descriptively. Standard descriptive statistic methods will be applied including number of patients, arithmetic mean, standard deviation, upper and lower quartiles, minimum, median and maximum. For categorical variables tables of frequencies (absolute and relative frequencies) will be presented. Number of patients for whom data is missing will be provided where appropriate.

Reported adverse events and comorbidities will be coded using MedDRA dictionary (version 23.1) and all adverse event summaries will present preferred terms and System Organ Class (SOC).

All data up to the time of study completion/withdrawal from study will be included in the analysis, regardless of duration of treatment.

Detailed description of study populations, including rules for inclusion and exclusion to a given population, data analysis and results presentation will be provided in a statistical analysis plan (SAP), a separate document provided by the BioStat Sp. z.o. prior to final database lock.

11.2 STATISTICAL HYPOTHESES

The null hypothesis assumes that proportion of subjects randomized to study treatment achieving a primary endpoint (p) is lower than proportion in placebo group (p0):

H0: $p\text{-}p0 \leq \delta$

Ha: $p\text{-}p0 > \delta$,

where δ is a non-inferiority margin (-15%).

11.3 SAMPLE SIZE DETERMINATION AND PLANNED RECRUITMENT RATES

Due to nature of this study (proof of concept phase II trial), with effect size being unknown, following assumptions were made:

- Proportion of subjects randomized to receive IMP achieving primary endpoint will be 40% and this proportion will be identical in a placebo group
- A non-inferiority margin δ will be equal to 15%
- One sided significance level 0.2
- Power of 70%
- One interim analysis (with Hwang-Shih-DeCani α pending function)
- A drop-out rate of 10%.
  Taking these into consideration, a minimal number of subjects per group is 46 – so 92 subjects in each study arm.

Sample size calculations were made in R version 3.6 using gsDesign library version 3.1.1.

11.4 POPULATIONS FOR ANALYSES

11.4.1 INTENT TO TREAT POPULATION (ITT)

A subset of all enrolled subjects consisting of subjects that were randomized independently of the fact if they have received study treatment. This population will be used for baseline characteristics and efficacy analysis.

11.4.2 PER PROTOCOL POPULATION (PP)

A subset of ITT population consisting of subjects that have functional status assessed with modified Rankin scale at 90 days (+/- 3 days) after study treatment administration. This population will be the main population used for efficacy analysis.

11.4.3 SAFETY ANALYSIS POPULATION (SAF)

A subset of intent to treat analysis population consisting of subjects that received study treatment. This population will be used for all safety analyses.

11.5 STATISTICAL ANALYSES

11.5.1 GENERAL ASSUMTIONS

11.5.1.1 STATISTICAL SIGNIFICANCE ASSUMTPIONS

Statistical significance level will be equal to 0.20 for purpose of primary endpoint analysis and 0.05 otherwise. P values ≥0.001 will be reported to 3 decimal places; p-values less than 0.001 will be reported as "<0.001". The mean, standard deviation, and any other statistics other than quantiles, will be reported to one decimal place greater than the original data. Quantiles, such as median, or minimum and maximum will use the same number of decimal places as the original data. Estimated parameters, not on the same scale as raw observations (e.g. regression coefficients) will be reported to 3 significant figures.

11.5.1.2 BASELINE DESCRIPTIVE STATISTICS

Age, sex, NIHSS, mRS, and Barthel index will be presented for Visit 0 overall and for each treatment arm and treatment group (study treatment and placebo).

11.5.1.3 TABULATION OF INDIVIDUAL PARTICIPANT DATA

Data will be listed as documented. Relevant generated and transformed variables will be listed next to the original data items. Any imputed value will be flagged.

In all listings the patient identifier and the randomized treatment group will be included. The patient identifier consists of the study centre and the patient number, additionally a flag specifying analysis set will be provided.

In general, patient listings will be sorted by patient identifier and visit (if applicable), unless otherwise stated.

Patient listings of data that is collected independently from visits (e.g., adverse events, medical histories, or medication) will be sorted by patient identifier, day of onset or start day of administration, duration and MedDRA preferred term or WHO base substance name, respectively.

Missing values in the listings will be represented as NA (not available) for both text and numerical data. In case of partially missing dates (when day or day and month is unknown) missing data will be represented as series of 9s – for example 2017-05 will be presented as 2017-05-99. Any imputed data will be presented separately from raw data or flagged accordingly to be able to review all data as collected.

11.5.2 EFFICACY ANALYSES

11.5.2.1 ANALYSIS OF THE PRIMARY ENDPOINT

Primary endpoint analysis for each study arm (dabigatran, apixaban, or rivaroxaban) will be performed independently. Proportion of subjects achieving primary endpoint for active treatment and placebo as well as difference of proportions together with 82% Confidence Interval (CI) will be calculated. One-sided noninferiority test with 15% margin for difference in proportions will be performed. A p value lower than 0.176 will be considered as statistically significant.

11.5.2.2 ANALYSIS OF THE SECONDARY ENDPOINT(S)

For each study arm (A, B or C) proportion of subjects randomized to placebo and active treatment and proportion difference together with 95% CI will be presented. Unadjusted and adjusted (with subject age) odds ratios will be shown. P value lower than 0.05 will be considered as statistically significant (α=0.05).

11.5.2.3 OTHER EFFICACY ANALYSES

Exploratory endpoints include additional cognitive and functional status assessments:

These endpoints will be analysed using descriptive statistics of the total scores presented by treatment in each study arm and overall, in both tabular and graphical form. In case of the latter, boxplots will be used for analysis of exploratory functional assessments.

- Barthel Index (see Section 9.1.4.1) - change from baseline Day 7 (Visit 3) and Day 90 (Visit 5) will be assessed using ANCOVA with baseline Barthel Index score and treatment as independent variables. LS mean difference between placebo and active drug with 95% confidence interval will be provided. If either the equal variance or the normality assumption appears to be grossly violated, other methods including an ANCOVA on ranks model or an appropriate transformation of endpoint might be considered.
- MoCA (see Section 9.1.4.2) - change from baseline (Day 7) to Day 90 90 will be analysed in the same way as Barthel Index.
- HADS (see Section 9.1.4.3) assessment of mental condition collected on Day 7 and Day 90 will be analysed in the same way as Barthel Index.
- IQCODE assessment on Day 7 (see Section 9.1.4.4)
- Brain natriuretic peptide (see Section 9.1.5) change between baseline (Screening visit) and Day 7 will be analysed in the same way as Barthel Index.

11.5.2.4 SAFETY ANALYSES

Safety data will be summarized for the safety population.

11.5.2.5 KEY SAFETY ENDPOINTS

Rate of early (symptomatic, and asymptomatic) intracranial haemorrhages detected with neuroimaging, incidence of major extracranial haemorrhages, deaths, recurrent ischaemic strokes, neurological deteriorations will be analysed in the same way as secondary endpoints.

11.5.2.6 ADVERSE EVENTS ANALYSIS

Any undesirable signs, symptoms or medical conditions occurring or worsening of pre-existing conditions between signing informed consent and the first administration study medication are considered as pre-treatment AEs. Undesirable signs, symptoms or medical conditions or worsening of pre-existing conditions occurring after the first administration of study medication are considered as treatment emergent adverse events (TEAEs).

A TEAE will be analysed as related to study medication (i.e. as adverse drug reaction, ADR) if the relationship to study treatment was documented as “unlikely”, “possible”, “probable”, or “definite” or if the relationship to study treatment is missing.

An overview table presenting the incidence of the following AE categories will be presented. Percentage of patients in each category will be compared between treatments as a difference in proportions between treatments including a 95% CI based on the (Agresti and Caffo, 2000).

All AEs

- Pre-treatment AEs
- Serious AEs (SAEs) (including pre-treatment SAEs)
- Treatment emergent SAEs (TESAEs)
- Severe TEAEs
- Related TEAEs
- Related severe TEAEs
- TEAEs leading to permanent discontinuation (i.e. withdrawal) of study medication
- Related TEAEs leading to permanent discontinuation of study medication
- AEs leading to death (i.e., outcome of AE is fatal)
- TEAEs leading to death
- Related TEAEs leading to death

All treatment emergent AEs (TEAEs) and all serious TEAEs will be tabulated by MedDRA SOC and PT presenting the number and percentage of patients reporting the AE and the number of AEs reported. Additional tables will distinguish the events by whether the AE was related to study medication and by maximum severity.

All AEs documented in the eCRF will be listed by patient. Data listings will include patient ID, treatment group, verbatim term, preferred term, system organ class, start and stop date and relative day of the AE, severity, medications (yes/no), seriousness, action taken with study medication, relationship to study medication, and outcome will be provided. TEAEs will be flagged as such. In addition, separate listings for all SAE’s, deaths and AEs leading to discontinuation (action taken with study treatment = drug withdrawal or AE is reason for early termination) will be presented.

11.5.2.7 SAFETY LABORATORY DATA

Laboratory data will be summarized by type of laboratory test after all results are converted to standard SI units. Descriptive statistics will be calculated for each laboratory parameter at baseline

and at each scheduled time point. Shift tables using the categories 'lower than normal range', 'within normal range' and 'higher than normal range' will be provided for each parameter, comparing the baseline value to all post-baseline values.

Listings of each laboratory parameter will be provided, marking changes from norm. A separate listing will be produced for clinically significant abnormal results.

11.5.2.8 OTHER SAFETY DATA

Vital signs summaries will take form of tables presenting descriptive statistics for BP and HR as well as shift tables of result categories presenting changes when compared to baseline (Screening visit). Listings flagging abnormal values will be also produced.

ECG variables that will be analysed include heart rate, QTc intervals and ECG findings. In case of heart rate values □50 bpm will be classified as abnormal low, while values ≥100 will be classified as abnormal high.

ECG summaries will take form of tables for raw data and changes from baseline (values collected during screening) as well as percentage of abnormal values. In addition, shift tables presenting number of patients that either developed abnormalities in heart rate or QTc (or had such abnormalities subsume) since baseline will be provided.

The number of patients with at least one clinically relevant finding detected during the physical examination or the ECG will be summarized in frequency tables for each scheduled assessment or visit.

11.5.3 OTHER ANALYSES

11.5.3.1 EXPLORATORY ANALYSES

Exploratory analyses will be described in an appropriate SAP before the analyses take place.

11.5.3.2 SUB-GROUPS ANALYSES

All subgroup analyses will be descriptive in nature. It will include primary, secondary and exploratory endpoints analyses.

Detailed definition of subgroups will be provided in SAP.

11.5.4 PLANNED INTERIM ANALYSES

Primary endpoint analysis for each study arm (dabigatran, apixaban, or rivaroxaban) will be performed independently. Proportion of subjects achieving primary endpoint for active treatment and placebo as well as difference of proportions together with 82% Confidence Interval (CI) will be calculated. One-sided noninferiority test with 15% margin for difference in proportions will be performed. A p value lower than 0.176 will be considered as statistically significant.

11.5.4.1 INTERIM ANALYSIS OF EFFICACY AND SAFETY ENDPOINTS

An interim analysis is planned after half of the randomized subjects reach primary endpoint (functional status assessment with modified Rankin scale on day 90). All collected data will be analysed. Primary endpoint will be tested using one sided α=0.0238.

11.5.4.2 PLANNED INTERIM ANALYSIS OF EARLY SAFETY DATA

Analysis well cover safety endpoints. For this purpose, data listings of adverse events, vital signs, adverse events, physical examinations, ECG and echocardiography will be prepared.

11.5.4.3 INDEPENDENT DATA MONITORING COMMITTEE (IDMC)

An IDMC (see Section 10.3.3) will be established to reassure participants overall safety and to monitor the progress of the clinical study at regular intervals. This will include reviewing unblinded safety and tolerability data and the efficacy data from the Interim Analysis.

12 SUPPORTING DOCUMENTATION AND OPERATIONAL CONSIDERATIONS

12.1 REGULATORY, ETHICAL, AND STUDY OVERSIGHT CONSIDERATIONS

This study will be conducted in accordance with the protocol and with the following:

- Consensus ethical principles derived from local and international guidelines including the Declaration of Helsinki
- Applicable ICH GCP Guidelines
- Applicable laws and regulations

The study protocol, protocol amendments, ICF, IB, and other relevant documents will be submitted to an IRB/IEC by the investigator for review and approval by the IRB/EC before the study is initiated.

Any amendments to the study protocol will require IRB/IEC and applicable Regulatory Authority approval before implementation of changes made to the study design, except for changes necessary to eliminate an immediate hazard to study participants.

50Bio CRO (on behalf of the Sponsor) will be responsible for obtaining the required authorisations to conduct the study from the concerned Regulatory Authority.

12.1.1 CASE REPORT FORMS (CRFS)

Study data will be collected via web-based electronic Case Report Form (eCRF).

BioStat Ltd. will be responsible for eCRF design, maintenance and oversight of the database created based on information entered in the eCRF

12.1.2 TRIAL DATA AND DOCUMENTATION HELD AT SITES

Study records and documents, including signed ICFs, related to the conduct of the STROACT study must be retained by the investigator according to local regulations or institutional policies No records may be destroyed during the retention period without the written notification of the Sponsor.

12.1.3 STUDY DISCONTINUATION AND CLOSURE

The study may be terminated before the planned end of study by the principal investigator's decision if it is found that the patients participating in the study are exposed to unnecessary health risks. Such decision should be based on IDMC recommendations and the well-being of patients will always be put first in the considerations before decision about premature study termination is taken.

12.1.4 CONFIDENTIALITY AND PRIVACY

All personnel involved in the STROACT study must comply with applicable data protection laws and regulations. To anonymize patient’s data collected during the study standard procedures will be applied.

- Participants will be assigned a unique identifier by each study centre (screening number specific for the patient and study centre) and also randomisation number (specific number in the study to allocate study treatment) which will be generated in the eCRF system (see Section 6.2 and Section 6.4). Any participant records or datasets that are transferred to the sponsor will contain the identifier only; participant names or any information which would make the participant identifiable will not be shared (transferred).

- The participant will be informed that his/her personal study-related data will be used by the Sponsor in accordance with local data protection law. The level of disclosure and use of their data will also be explained to the participant in the informed consent.

12.1.5 FUTURE USE OF STORED SPECIMENS AND DATA

There is no plan to collect specimens for future evaluation or analyses.

Clinical data obtained during the study can be used for future analyses, if deemed appropriate by participating Investigators.

12.1.6 KEY ROLES AND STUDY GOVERNANCE

The EC (see Section 10.3.1) will provide operational management of the STROACT study including protocol design and general oversight of the study conduct. Specific responsibilities will be delegated to CROs as appropriate.

The SC (see Section 10.3.2) will be responsible for implementation and oversight of study procedures at the study centre level, monitor progress in enrolment, and contribute to the ongoing analysis and interpretation of incoming clinical efficacy and safety data for individual patients.

12.1.7 SAFETY OVERSIGHT

The investigator and any designees at each study centre are responsible for patient safety during participation in the STROACT study.

To reassure adequate safety oversight at the study level the IDMC will be established (see Section 10.3.3).

Individual safety information will be monitored on the ongoing basis and accumulated data will be reviewed periodically. Specification of key safety date, frequency and format will be described in Safety Management Plan (SMP) and SAP.

12.1.8 CLINICAL MONITORING

50Bio (CRO) will be responsible for clinical monitoring during the study duration and will follow own applicable SOPs, unless agreed otherwise with the Sponsor.

12.1.9 PROTOCOL DEVIATIONS AND VIOLATIONS

For purposes of the STROACT clinical trial, a **protocol deviation (PD)** is defined as a departure from the approved **protocol's** procedures made with or without prior EC/IRB approval. Such departures may be major or minor/administrative in nature.

Protocol violation (PV) is defined as an important **protocol deviation** which may have potential impact on safety of the research participant or scientific integrity of the study

Study will be monitored for occurrence of PDs/PVs on the ongoing basis and identified cases will be assessed and reported according to the local competent authorities and appropriate EC/IRB requirements.

Identified PDs/PVs will be also reported in the Clinical Study Report.

Prospective approval of protocol deviations to recruitment and enrolment criteria, also known as protocol waivers or exemptions, is not permitted.

12.1.10 STUDY RECORDS RETENTION

Each site / Principal Investigator is responsible for study records retention. Records and documents, including signed ICFs, pertaining to the conduct of this study must be retained by the investigator in accordance with local regulations after study completion unless local regulations or institutional policies require a longer retention period.

12.1.11 FINANCING AND INSURANCE

The STROACT project is supported by the grant from the Medical Research Agency based on a proposal from the Medical University of Gdańsk No: 2019/ABM/01/00084.

The sponsor is responsible for the possession of the OC insurance for damage caused in connection with the conduct of the clinical trial, in accordance with the rules laid down in the Regulation of the Minister of Finance of 30 April 2004 on compulsory insurance against civil liability of the investigator and sponsor.

12.1.12 PUBLICATION AND DATA SHARING POLICY

The results of the STROACT study are intended to be published and/or presented at scientific meetings. The EC will be responsible for study data analysis, presenting and publishing (see also Section 10.3.1)

12.2 ABBREVIATIONS

Abbreviation	Description
ADL	Activities of Daily Living
AE	Adverse Events
AF	Atrial Fibrillation
aICH	Asymptomatic Intracranial Haemorrhage
AIS	Acute Ischaemic Stroke
AHA	American Heart Association
AlAT	Alanine aminotransferase
aPTT	Activated partial thromboplastin time
ASA	American Stroke Association
ASPECTS	Alberta Stroke Program Early CT Score
BI	Barthel Index
BNP	Brain natriuretic peptide
BP	Blood Pressure
CBC	Complete Blood Count
CRP	C-reactive protein
CSR	Clinical Study Report
CT	Computed Tomography
CTA	CT angiography
CTCAE	Common Terminology Criteria for Adverse Events
CTP	Clinical Trial Protocol
DBP	Diastolic blood pressure
EC	Executive Committee
eCRF	Electronic Case Report Form
eGFR	Estimated Glomerular Filtration Rate
ECASS	European Cooperative Acute Stroke Study
ECG	Electrocardiogram
EOS	End of Study
ESO	European Stroke Organisation
HADS	Hospital Anxiety and Depression Scale
HR	Heart Rate

IB	Investigator’s Brochure
ICF	Informed Consent Form
ICH	Intracranial Haemorrhage
IDMC	Independent Data Monitoring Committee
INR	International normalised ratio
IS	Ischaemic stroke
IQCODE	Informant Questionnaire on Cognitive Decline in the Elderly
IVT	Intravenous Thrombolysis
LPI	Lead Principal Investigator
LVO	Large Vessel Occlusion
MoCA	Montreal Cognitive Assessment
MRA	MRI angiography
MRI	Magnetic resonance imaging
mRS	Modified Rankin Scale
MT	Mechanical thrombectomy
NIHSS	National Institute of Health Stroke Scale
MRI	Magnetic Resonance Imaging
NOAC	Non-Vitamin K Antagonist Oral Anticoagulant
OAC	Oral Anticoagulant
PI	Principal Investigator
rtPA	Recombinant tissue plasminogen activator
SAE	Serious Adverse Event
SAP	Statistical Analysis Plan
SBP	Systolic blood pressure
SC	Steering Committee
sICH	Symptomatic Intracranial Haemorrhage
SITS-MOST	Safe Implementation of Thrombolysis in Stroke-Monitoring Study
SmPC	Summary of Product Characteristics
SoA	Schedule of Assessments
SOC	System Organ Class
SUSAR	Suspected Unexpected Serious Drug Reaction
STROACT	**STR**oke on **O**ral **A**nti**C**oagulants for **T**hrombolysis
TFPI	Tissue Factor Pathway Inhibitor

13 REFERENCES

Actilyse SmPC. (2019). *Summary of Product Characteristics.*

Agresti and Caffo. (2000). Simple and Effective Confidence Intervals for Proportions and Differences of Proportions Result from Adding Two Successes and Two Failures. *Am Statistician, 54*, 280–288.

Connolly SJ et al. (2019). Full Study Report of Andexanet Alfa for Bleeding Associated with Factor Xa Inhibitors. *N Engl J Med, 380*, 1326-35.

Emberson J et al. (2014). Effect of treatment delay, age, and stroke severity on the effects of intravenous thrombolysis with alteplase for acute ischaemic stroke: a meta-analysis of individual patient data from randomised trials. *Lancet, 384*, 1929–1935.

ESC Guidelines. (2016). 2016 ESC Guidelines for the management of atrial fibrillation developed in collaboration with EACTS. *Eur Heart J, 37*, 2893-2962.

Feigin VL et al. (2019). Global, regional, and national burden of stroke, 1990-2016: a systematic analysis for the Global Burden of Disease Study 2016. *Lancet Neurol, 18*, 439-458.

Hacke W et al. (2008). Thrombolysis with Alteplase 3 to 4.5 Hours after Acute Ischemic Stroke. *N Engl J Med, 359*, 1317-29.

Hindricks G et al. (2020). ESC Scientific Document Group, 2020 ESC Guidelines for the diagnosis and management of atrial fibrillation developed in collaboration with the European Association for Cardio-Thoracic Surgery (EACTS): The Task Force for the diagnosis and management of atr. *Eur Heart J*, ehaa612. Retrieved from https://doi.org/10.1093/eurheartj/ehaa612

Karaszewski B et al. (2015). What causes intracerebral bleeding after thrombolysis for acute ischaemic stroke? Recent insights into mechanisms and potential biomarkers. Journal of Neurology,. *J Neurol Nuerosurg Psych, 86*, 1127-1136.

Kerr DM et ala. (2012). Seven-Day NIHSS Is a Sensitive Outcome Measure for Exploratory Clinical Trials in Acute Stroke Evidence From the Virtual International Stroke Trials Archive. *Stroke, 43*, 1401-1403.

Lindsay MP et al. (2019). World Stroke Organization (WSO): Global Stroke Fact Sheet 2019. International Journal of Stroke. *Int J Stroke, 14*, 806-817.

Lip GY et al. (2010). Refining clinical risk stratification for predicting stroke and thromboembolism in atrial fibrillation using a novel risk factor-based approach: the euro heart survey on atrial fibrillation. *Chest, 137*, 263-272.

Meyer L et al. (2020). Early clinical surrogates for outcome prediction after stroke thrombectomy in daily clinical practice. Meyer L, Broocks G, Bechstein M, et al. *J Neurol Neurosurg Psychiatry, 91*, 1055–1059.

Nunn A et al. (2016). Analysis of the Modified Rankin Scale in Randomised Controlled Trials of Acute Ischaemic Stroke: A Systematic Review. Stroke Research and Treatment. *Stroke Res Treatment, Article ID 9482876*, 1-7.

Ondexxya SmPC. (2020, 11 20). *Summary of Product Characteristics.* Retrieved from EMA: https://www.ema.europa.eu/en/documents/product-information/ondexxya-epar-product-information_en.pdf

PNS Guidelines. (2019). Polish Neurological Society: Guidelines for stroke management (in Polish). *Pol Przegl Neurol, 15 (Suppl)*, A1-155.

Pollack CV et al. (2017). Pollack CV et al. 2017: Idarucizumab for Dabigatran Reversal — Full Cohort Analysis. *N Engl J Med, 377*, 431-41.

Powers WJ et al. (2019). Guidelines for the Early Management of Patients With Acute Ischemic Stroke: 2019 Update to the 2018 Guidelines for the Early Management of Acute Ischemic Stroke. A Guideline for Healthcare Professionals From the American Heart Association/American Stroke. *Stroke, 50*, e344–e418.

Praxbind SmPC. (2020, 10 16). *Summary of Prodcut Characteristics.* Retrieved from EMA: https://www.ema.europa.eu/en/documents/product-information/praxbind-epar-product-information_en.pdf

Sandercock P et al. (2012). The benefits and harms of intravenous thrombolysis with recombinant tissue plasminogen activator within 6 h of acute ischaemic stroke (the third international stroke trial [IST-3]): a randomised controlled trial. *Lancet 2012; 379:, 379*, 2352-63.

Saver JL. (2011). Optimal Endpoints for Acute Stroke Therapy Trials: Best Ways to Measure Treatment Effects of Drugs and Devices. *Stroke, 42*, 2356–2362.

Whiteley WN et al. (2016). Risk of intracerebral haemorrhage with alteplase after acute ischaemic stroke: a secondary analysis of an individual patient data meta-analysis. *Lancet Neurol, 15*, 925-933.

Yaghi S et al. (2014). Symptomatic intracerebral hemorrhage in acute ischemic stroke after thrombolysis with intravenous recombinant tissue plasminogen activator: A review of natural history and treatment. *JAMA Neurol, 71*, 1181-1185.

14 APPENDICES

14.1 ADVERS EVENTS: DEFINITIONS AND PROCEDURES FOR RECORDING, EVALUATING, FOLLOW-UP, AND REPORTING

14.1.1 DEFINITION OF ADVERSE EVENTS (AE)

An adverse event (AE) is defined as the development of any untoward medical occurrence in a patient / study participant administered a medicinal product / intervention and which does not necessarily have a causal relationship with this treatment. An AE can therefore be any unfavourable and unintended sign (for example an abnormal laboratory result) and symptom (for example nausea, pain), or disease temporally associated with the use of a medicinal (investigational) product / intervention, whether or not related to the medicinal (investigational) product / intervention.

AE includes complaints reported by the patient (patient interview), abnormalities found during the patient's examination (physical examination), or ancillary examinations (abnormal laboratory tests, findings in radiological examinations).

The term AE includes both serious and non-serious AEs and can include a worsening / deterioration (for example an increase in their frequency, intensity, or severity) of a medical condition pre-existing (including chronic diseases) before inclusion in the study. An AE may occur at any time, including screening even when no study treatment has been administered.

In this study AEs will be reported and recorded in the appropriate eCRF section from the moment of obtaining informed consent until end of study (Visit 6; End of Study).

14.1.2 DEFINITION OF SERIOUS ADVERSE EVENTS (SAE)

A serious adverse event (SAE) is defined as an AE occurring during the study that fulfils one or more of the following criteria:

- Results in death
- Is (immediately) life-threatening,
- Requires in-patient hospitalization or prolongation of existing hospitalization,
- Results in persistent or significant disability/incapacity, or
- Is a congenital anomaly or birth defect
- Is an important medical event that may jeopardise the patient or may require medical treatment to prevent one of the outcomes listed above

Notes / additional explanations:

Life-threatening

"Life-threatening" means that the patient / study participant was at immediate risk of death from the AE as it occurred, or it is suspected that use or continued use of the product would result in the patient's death. "Life-threatening" does not mean that the patient / study participant had an AE in a more severe form

Hospitalisation

Outpatient treatment in an emergency room is not in itself a serious AE (SAE), although the reasons for it may be (e.g., bronchospasm, laryngeal oedema). Hospital admissions and/or surgical operations planned before or during a study are not considered AEs, if the illness or disease existed before the patient was enrolled in the study and providing that it did not deteriorate in an unexpected way during the study.

Important medical event

Important medical event means an AE that may not be immediately life-threatening or result in death or hospitalisation but may jeopardise the subject or require medical or surgical intervention to prevent one of the outcomes listed for SAE.

14.1.3 EVENTS OF SPECIAL INTEREST

Events of Special Interest (ESI) are those events thought to be associated, or potentially associated with the investigational treatment and/or AIS course. In the STROACT study there are prespecified AEs / events which are considered as ESI. These events will be monitored during the study and will be analysed as the critical safety endpoints comparing the study treatment and placebo groups.

- Mortality at 7-, 30- and 90-days post AIS:
- Neurovascular events at 7-, 30- and 90-days post AIS:
  - Recurrent ischaemic stroke
  - Neurological deterioration (NIHSS)
- Incidence of intracranial bleeding (symptomatic and asymptomatic) assessed with CT/MRI and assessed according to European Cooperative Acute Stroke Study (ECASS II) classification at 24h and 7 days after study treatment administration.
- Incidence and severity of major extracranial haemorrhage defined as:
  - fatal
  - severe enough to require transfusion or operation
  - an absolute decrease in haemoglobin > 5 g/dL
  - a decrease in haematocrit of > 15%
  - bleeding associated with persistent or serious disability)
- Post-treatment thromboembolic and cardiovascular events.

14.1.4 ADVERSE REACTION (AR)

Adverse reaction (AR) to an investigational medicinal product is defined as all untoward and unintended responses to an investigational medicinal product related to any dose administered. All adverse events judged by either the reporting investigator or the sponsor as having a reasonable causal relationship to a medicinal product qualify as adverse reactions. The expression reasonable causal relationship means to convey in general that there is evidence or argument to suggest a causal relationship.

14.1.5 SUSPECTED UNEXPECTED SERIOUS ADVERSE REACTION

A Suspected Unexpected Serious Adverse Reaction (SUSAR) is defined as an untoward and unintended response to a study drug, which is not listed is the applicable product information, and meets one of the following serious criteria: results in death, is life-threatening, requires hospitalisation or prolongation of an existing hospitalisation, results in persistent or significant disability or incapacity, or is a congenital anomaly or birth defect.

14.1.6 ASSESSMENT OF ADVERSE EVENTS

The National Cancer Institute CTCAE latest version (CTCAE version 5.03) will be used for assessing AE intensity (severity of event) for all events with an assigned CTCAE grading. For those events without assigned CTCAE grades, the recommendation in the CTCAE criteria on converting mild, moderate, and severe events into CTCAE grades should be used.

A copy of the CTCAE can be downloaded from the website http://ctep.cancer.gov.

14.1.6.1 SEVERITY OF EVENT

14.1.6.2 RELATIONSHIP TO STUDY INTERVENTION

The investigator will assess causal relationship between drug / study intervention and each AE. Assessing causality (relationship to study intervention) the following factors when deciding if there is a "reasonable possibility" that an AE may have been caused by the drug / intervention should be considered:

- Time Course / Exposure to suspect drug.
  (Has the patient actually received the suspect drug? Did the AE occur in a reasonable temporal relationship to the administration of the suspect drug?)
- Consistency with known drug profile.
  (Was the AE consistent with the previous knowledge of the suspect drug [pharmacology and toxicology] or drugs of the same pharmacological class? Or could the AE be anticipated from its pharmacological properties?)
- De-challenge experience.
  (Did the AE resolve or improve on stopping or reducing the dose of the suspect drug?)
- No alternative cause.
  (The AE cannot be reasonably explained by another aetiology such as the underlying disease, other drugs, other host or environmental factors.)
- Re-challenge experience.
  (Did the AE reoccur if the suspected drug was reintroduced after having been stopped?)
- Laboratory tests. A specific laboratory investigation (if performed) has confirmed the relationship.

14.2 PERFORMANCE AND FUNCTIONAL ASSESSMENT FORMS

14.2.1 NIHSS ASSESMENT FORM

The Polish version of the Barthel Index Assessment Form.

Skala Udarów Narodowego Instytutu Zdrowia **Data:**
National Institutes of Health Stroke Scale (NIHSS) **Godzina:**

**1A. Poziom świadomości**<br>- pełna, chory czuwający **0**<br>- chory podsypiający, wybudza się przy niewielkiej stymulacji SOMNOLENCJA **1**<br>- chory głęboko senny, w celu wybudzenia konieczne wzmocnienie bodźca lub bodziec bolesny STUPOR **2**<br>- śpiączka (prężenia ruchowe i/lub odruchy autonomiczne, wzgl. brak reakcji) **3**					
**1B. Odpowiedź na pytanie o miesiąc i wiek (**liczy się pierwsza odpowiedź)<br>- prawidłowa na oba pytania **0**<br>- prawidłowa na jedno lub dyzartria, intubacja, bariera językowa **1**<br>- obie nieprawidłowe lub brak odpowiedzi **2**					
**1C. Spełnianie poleceń** (zamknięcia lub otwarcia oczu, oraz wyprostowania lub zgięcia palców ręki)<br>- spełnia oba **0**<br>- spełnia jedno **1**<br>- nie spełnia żadnego **2**					
**2. Zbaczanie głowy i gałek ocznych** (ruchy gałek ocznych w prawo i w lewo)<br>- brak zbaczania, ruchy gałek w pełnym zakresie **0**<br>- częściowe zbaczanie gałek ocznych, nieutrwalone, ustępujące przy manewrze oczno-głowowym **1**<br>- utrwalone zbaczanie, nieustępujące **2**					
**3. Pole widzenia** (prezentacja bodźca w poszczególnych kwadrantach pola widzenia)<br>- bez ubytków **0**<br>- częściowe niedowidzenie połowicze (np. kwadrantowe) **1**<br>- pełne niedowidzenie połowicze **2**<br>- ślepota (np. korowa) lub obustronne niedowidzenie połowicze **3**					
**4. Czynność nerwu twarzowego** (pacjent proszony o uśmiech, uniesienie brwi, zaciśnięcie powiek)<br>- symetria twarzy zachowana **0**<br>- nieznaczne wygładzenie fałdu nosowo-wargowego, asymetria przy uśmiechu **1**<br>- wyraźne obniżenie kącika ust **2**<br>- porażenie mięśni połowy twarzy (jedno- lub obustronnie) **3**					
**5. Siła mięśniowa kończyny górnej** (uniesienie wyprostowanej kończyny przez 10 sekund)<br>- gdy utrzymuje kończynę pod kątem 45° przez 10 sekund (także amputacja lub patologia stawu) **0**<br>**lewa**<br>- gdy kończyna zaczyna opadać przed upływem 10 sekund **1**					
- nie utrzymuje kończyny w poziomie, ale widoczny jest opór przeciw sile ciężkości **2**<br>**prawa**<br>- kończyna opada natychmiast, ślad ruchu **3**<br>- całkowity brak ruchów czynnych **4**					
**6. Siła mięśniowa kończyny dolnej** (uniesienie wyprostowanej kończyny przez 5 sekund)<br>- gdy utrzymuje uniesioną pod kątem 30° przez 5 sekund (także amputacja lub patologia stawu) **0**<br>- gdy kończyna powoli opada przed upływem 5 sekund **1**<br>**lewa**					
- opada szybko przed upływem 5 sek., lecz zachowany jest opór przeciw sile ciężkości **2**<br>**prawa**<br>- opada natychmiast, ślad ruchów **3**<br>- całkowity brak ruchów czynnych **4**					

**7. Ataksja** (próba palec-nos, pięta-kolano)<br>- brak (także amputacja, patologia stawu, niedowład lub brak rozumienia) **0**<br>**-** obecna w kończynie górnej lub dolnej **1**<br>- obecna w obu kończynach **2**					
**8. Czucie** (badane przez ukłucie w zakresie twarzy, kończyn, tułowia), gdy afazja lub stupor 0 lub 1 pkt.<br>- prawidłowe **0**<br>- zaburzenia w stopniu niewielkim lub umiarkowanym, tj. osłabienie czucia bólu lub ukłucie odczuwalne jako tępe, ewent. pacjent odczuwa tylko dotyk **1**<br>- znacznie osłabione, lub brak (także śpiączka i/lub porażenie czterokończynowe) **2**					
**9. Język** (nazywanie przedmiotów, opisanie obrazu, czytanie/powtarzanie tekstu)<br>- bez zaburzeń **0**<br>- błędne nazywanie, niezdolność do dobierania właściwego słowa „parafazje", lub/i zaburzenia rozumienia 1<br>- zaburzenia znacznego stopnia: w pełni rozwinięta afazja typu Broca lub Wernickego (ewent. inny rodzaj) 2<br>- całkowita afazja, mutyzm, śpiączka<br>**3**					
**10. Dyzartria** (wymowa, czytanie względnie powtarzanie słów)<br>- brak (także intubacja lub brak możliwości zbadania) **0**<br>- umiarkowana, rozumiany z trudem „bełkocze", możliwa komunikacja werbalna **1**<br>- znacznego stopnia, słowa całkowicie niezrozumiałe, mutyzm **2**					
**11. Zespół nieuwagi połowiczej (modalność wzrokowa, słuchowa, czuciowa, przestrzenna, osobowa)**<br>- bez zaburzeń **0**<br>- wygaszanie bodźca jednego rodzaju (czucia, słuchu, wzroku) **1**<br>- nasilone objawy/ wygaszanie więcej niż jednego rodzaju bodźców **2**					
**Wynik badania:**					

14.2.2 MRS ASSESSMENT FORM

The Polish version of the mRS Assessment Form.

DATA	NAZWISKO I IMIĘ PACJENTA	OSOBA BADAJĄCA

Źródło informacji (można wybrać więcej niż jedno): ☐ pacjent ☐ rodzina pacjenta ☐ inne proszę podać: ________________

5 PACJENT LEŻĄCY	
**5.1 Czy pacjent jest osobą leżącą?**<br>Pacjent nie jest w stanie chodzić nawet z pomocą drugiej osoby.<br>Wymaga ciągłej obecności opiekuna lub nie kontroluje mikcji/wypróżnień.	☐ **Tak (mRS = 5)**<br>☐ Nie

Jeżeli tak, wyjaśnij ____

____

4 POMOC W CHODZENIU / PRZEMIESZCZANIU SIĘ POZA ŁÓŻKIEM	
**4.1 Czy pomoc drugiej osoby jest niezbędna do przemieszczaniu się poza łóżkiem?**<br>Pomoc drugiej osoby jest niezbędna przy każdej próbie chodzenia/poruszania się poza łóżkiem. Przez pomoc rozumie się zarówno bezpośrednią pomoc fizyczną, instruktaż słowny jak i bierny nadzór.<br><br>Pacjent oceniany jest jako osoba chodząca również jeżeli porusza się samodzielnie korzystając z pomocy ortopedycznych (m.in. chodzika lub wózka inwalidzkiego), ale nie wymaga żadnej pomocy innych (np. by dokonać transferu na wózek)	☐ **Tak (mRS = 4)**<br>☐ Nie

Jeżeli tak, wyjaśnij ____

____

3	POMOC W ZAJMOWANIU SIĘ SPRAWAMI WŁASNYMI PACJENTA	
	Przez pomoc rozumie się nie tylko bezpośrednią pomoc fizyczną, ale też instruktaż słowny lub bierny nadzór.<br>**Zasadnicze pytanie: Czy pacjent mógłby funkcjonować bez jakiejkolwiek pomocy osób trzecich przez 7 dni, gdyby był do tego zmuszony?**	
3.1	**Czy pomoc jest absolutnie konieczna do przygotowania prostego posiłku?**<br>(na przykład śniadania lub przekąski)	☐ **Tak (mRS = 3)**<br>☐ Nie
3.2	**Czy pomoc jest absolutnie konieczna do wykonywania podstawowych obowiązków domowych ?**<br>(np. przygotowanie świeżego ubrania i odłożeniu ubrania do prania, posprzątanie po posiłku; nie dotyczy obowiązków, które nie musza być wykonywane codziennie – np. odkurzania podłóg)	☐ **Tak (mRS = 3)**<br>☐ Nie
3.3	**Czy pomoc jest absolutnie konieczna do zajmowania się sprawami finansowymi własnego gospodarstwa domowego?**	☐ **Tak (mRS = 3)**<br>☐ Nie
3.4	**Czy pomoc jest absolutnie niezbędna do odbycia lokalnej podróży?**<br>(pacjent może prowadzić samochód, korzystać ze środków transportu publicznego lub taksówki, o ile jest w stanie sam ją wezwać i poinstruować kierowcę)	☐ **Tak (mRS = 3)**<br>☐ Nie
3.5	**Czy pomoc jest absolutnie konieczna przy robieniu podstawowych zakupów?**<br>(wystarczająca jest możliwość nabycia przynajmniej jednego przedmiotu na raz)	☐ **Tak (mRS = 3)**<br>☐ Nie

Przy jakich podstawowych czynnościach dnia codziennego pacjent wymaga pomocy i kto tej pomocy udziela

____

____

Rankin Focused Assessment - Ambulation w wersji 3.0 z 17.05.2013r. (Patel R i wsp. *J Stroke Cerebrovasc Dis* 2016;25:2172-6.)
W modyfikacji własnej uwzględniającej m.in. dodatkowe instrukcje z wersji 2.0 z 01.04.2009r.
Tłumaczenie na język polski: M.Karliński, Instytut Psychiatrii i Neurologii, Warszawa, luty 2020r.

Strona 1 z 2

DATA	NAZWISKO I IMIĘ PACJENTA	OSOBA BADAJĄCA

2	ZWYKŁE OBOWIĄZKI I CZYNNOŚCI	
2.1	<u>**Praca:**</u><br>**Czy bieżący udar w sposób znaczący ograniczył (w porównaniu ze stanem wcześniejszym) zdolność pacjenta do pracy zawodowej lub nauki?**<br>Na przykład: zmiana wymiaru zatrudnienia na niepełny etat, zmiana zakresu obowiązków lub całkowity brak zdolności do pracy / nauki.	☐ **Tak (mRS = 2)**<br>☐ Nie
2.2	<u>**Odpowiedzialność wobec pozostałych członków rodziny:**</u><br>**Czy bieżący udar w sposób znaczący ograniczył (w porównaniu ze stanem wcześniejszym) zdolność pacjenta do opiekowania się własną rodziną?**	☐ **Tak (mRS = 2)**<br>☐ Nie
2.3	<u>**Życie towarzyskie i aktywności w czasie wolnym:**</u><br>**Czy bieżący udar ograniczył (w porównaniu ze stanem wcześniejszym) dotychczasowe regularne aktywności prowadzone w czasie wolnym od pracy o więcej niż o połowę?**<br>Aktywności w czasie wolnym obejmują uprawianie hobby i realizację zainteresowań. Dotyczą zarówno aktywności wykonywanych poza domem (np. chodzenie do kawiarni, barów/pubów, restauracji, klubów, kościoła, kina, odwiedzanie przyjaciół, spacerowanie) oraz aktywności wykonywanych w domu (np. „aktywne" uczestnictwo, takie jak robienie na drutach, szycie, malowanie, granie w gry towarzyskie, czytanie książek, majsterkowanie).	☐ **Tak (mRS = 2)**<br>☐ Nie
2.4	<u>**Ograniczenia wynikające z innych schorzeń lub ograniczeń fizycznych:**</u><br>**Czy zdolność do pracy zawodowej / nauki, opiekowania się własną rodziną lub aktywności prowadzone w czasie wolnym od pracy zostały w sposób znaczący zaburzone przez schorzenia lub ograniczenia fizyczne niezwiązane z udarem ?**	☐ **Tak (mRS = 2)**<br>☐ Nie

Zmiany jakie zaszła pomiędzy stanem sprzed udaru a chwila obecną z odnotowaniem ich związku z udarem

____

____

____

____

1	REZYDUALNE OBJAWY UDAR MÓZGU	
1. 1	**Czy pacjent ma jakiekolwiek negatywne objawy wynikające z udaru mózgu?**<br>Problemy z czytaniem/pisaniem, mową, równowagą lub koordynacją, widzeniem, zaburzenia czucia, osłabienie siły mięśniowej, zaburzenia połykania lub jakiekolwiek inne trwałe negatywne skutki udaru mózgu.	☐ **Tak (mRS = 1)**<br>☐ Nie (mRS = 0)
1.2	**Czy pacjent w związku z objawami wynikającymi ze schorzeń innych niż bieżący udar mózgu ograniczył w sposób odczuwalny wykonywane uprzednio aktywności?**	☐ **Tak (mRS = 1)**<br>☐ Nie (mRS = 0)

Proszę odnotować rezydualne objawy bieżącego udaru mózgu oraz objawy niewynikające bezpośrednio z udaru

____

____

____

OSTATECZNA OCENA W ZMODYFIKOWANEJ SKALI RANKINA<br>(w razie wątpliwość, który z dwóch stopni niesprawności najlepiej oddaje stan pacjenta - należy wybrać wyższy z nich)
**punktacja mRS:**________________ **Podpis osoby oceniającej:**________________________________

Rankin Focused Assessment - Ambulation w wersji 3.0 z 17.05.2013r. (Patel R i wsp. *J Stroke Cerebrovasc Dis* 2016;25:2172-6.)
W modyfikacji własnej uwzględniającej m.in. dodatkowe instrukcje z wersji 2.0 z 01.04.2009r.
Tłumaczenie na język polski: M.Karliński, Instytut Psychiatrii i Neurologii, Warszawa, luty 2020r.

Strona 2 z 2

14.2.3 BARTHEL INDEX ASSESSMENT FORM

The Polish version of the Barthel Index Assessment Form.

WZÓR

KARTA OCENY ŚWIADCZENIOBIORCY

KIEROWANEGO DO OBJĘCIA /OBJĘTEGO[1)] PIELĘGNIARSKĄ OPIEKĄ

DŁUGOTERMINOWĄ DOMOWĄ

1. Ocena świadczeniobiorcy wg skali Barthel[2)]

Imię i nazwisko świadczeniobiorcy

....

Adres zamieszkania

....

Numer PESEL, a w przypadku braku numeru PESEL, numer dokumentu potwierdzającego tożsamość

....

Ocena świadczeniobiorcy wg skali Barthel

Lp.	Czynność[3)]	Wynik[4)]
1.	**Spożywanie posiłków:**<br>**0 -** nie jest w stanie samodzielnie jeść<br>**5 -** potrzebuje pomocy w krojeniu, smarowaniu masłem, itp., lub wymaga zmodyfikowanej diety<br>**10** - samodzielny, niezależny	
2.	**Przemieszczanie się z łóżka na krzesło i z powrotem, siadanie:**<br>**0** - nie jest w stanie; nie zachowuje równowagi przy siedzeniu<br>**5** - większa pomoc fizyczna (jedna lub dwie osoby)<br>**10** - mniejsza pomoc słowna lub fizyczna<br>**15** - samodzielny	
3.	**Utrzymywanie higieny osobistej:**<br>**0** - potrzebuje pomocy przy czynnościach osobistych<br>**5** - niezależny przy myciu twarzy, czesaniu się, myciu zębów (z zapewnionymi pomocami)	
4.	**Korzystanie z toalety (WC)**<br>**0** - zależny<br>**5** - potrzebuje pomocy, ale może coś zrobić sam<br>**10** - niezależny, zdejmowanie, zakładanie, ubieranie się, podcieranie się	
5.	**Mycie, kąpiel całego ciała:**<br>**0** - zależny<br>**5** - niezależny lub pod prysznicem	
6.	**Poruszanie się po powierzchniach płaskich:**<br>**0** - nie porusza się lub < 50 m<br>**5** - niezależny na wózku; wliczając zakręty > 50 m<br>**10** - spacery z pomocą słowną lub fizyczną jednej osoby > 50 m<br>**15** - niezależny, ale może potrzebować pewnej pomocy, np. laski > 50m	

7.	**Wchodzenie i schodzenie po schodach:**<br>**0** - nie jest w stanie<br>**5** - potrzebuje pomocy słownej, fizycznej; przenoszenie<br>**10** - samodzielny	
8.	**Ubieranie się i rozbieranie:**<br>**0** - zależny<br>**5** - potrzebuje pomocy, ale może wykonywać połowę czynności bez pomocy<br>**10** - niezależny w zapinaniu guzików, zamka, sznurowadeł, itp.	
9.	**Kontrolowanie stolca /zwieracza odbytu:**<br>**0** - nie panuje nad oddawaniem stolca lub potrzebuje lewatyw<br>**5** - czasami popuszcza (zdarzenia przypadkowe)<br>**10** - panuje, utrzymuje stolec	
10.	**Kontrolowanie moczu /zwieracza pęcherza moczowego:**<br>**0** - nie panuje nad oddawaniem moczu lub cewnikowany i przez to niesamodzielny<br>**5** - czasami popuszcza (zdarzenia przypadkowe)<br>**10** - panuje, utrzymuje mocz	
	**Wynik kwalifikacji**[5)]	

....

....

....

data, podpis i pieczątka pielęgniarki ubezpieczenia zdrowotnego albo pielęgniarki opieki długoterminowej domowej

2. Wynik oceny stanu zdrowia:

Stwierdzam, że wyżej wymieniona osoba wymaga / nie wymaga[1)] pielęgniarskiej opieki długoterminowej domowej.

....

....

....

data, podpis i pieczątka lekarza ubezpieczenia zdrowotnego

[1)] Niepotrzebne skreślić
[2)] Mahoney FI, Barthel D. "Badanie funkcjonalne: Wskaźnik Barthel." Maryland State Med Journal 1965; 14:56-61. Wykorzystane za zgodą. Skala ta może być używana bez ograniczeń dla celów niekomercyjnych.
[3)] W Lp. 1-10 należy wybrać i podkreślić jedną z możliwości najlepiej opisującą stan świadczeniobiorcy
[4)] Należy wpisać wartość punktową przypisaną wybranej możliwości
[5)] Należy wpisać uzyskaną sumę punktów

14.2.4 MOCA ASSESSMENT FORM

The Polish version of the Barthel Index Assessment Form.

MONTREAL COGNITIVE ASSESSMENT (MOCA®)
Version 8.1 Polish (Poland)

Nazwisko:
Wykształcenie:
Płeć:

Data urodzenia:
DATA:

PUNKTY

FUNKCJE WZROKOWO-PRZESTRZENNE

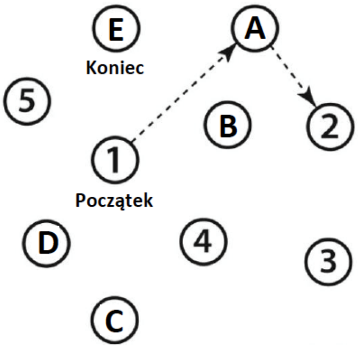

[ ]

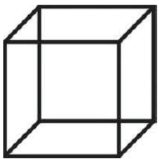

Skopiuj sześcian

[ ]

Narysuj ZEGAR. (Dziesięć po jedenastej)
(3 pkt.)

[ ] Kontur [ ] Liczby [ ] Wskazówki

__/5

NAZYWANIE

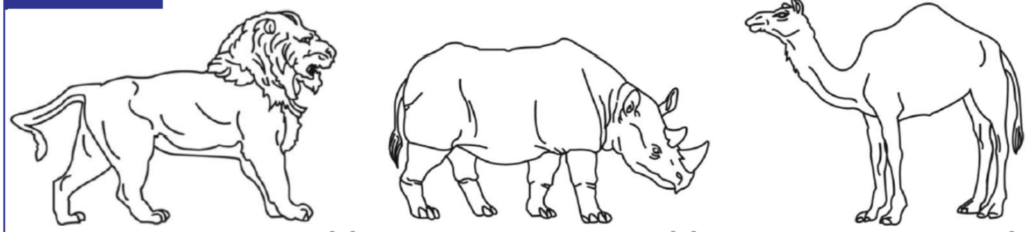

[ ] [ ] [ ]

__/3

PAMIĘĆ

Przeczytaj listę wyrazów. Badany/a powinien/powinna je powtórzyć. Przeprowadź 2 próby, nawet jeśli pierwsza zakończy się sukcesem. Poproś o powtórzenie wyrazów ponownie po 5 minutach.

	TWARZ	AKSAMIT	KOŚCIÓŁ	STOKROTKA	CZERWONY
PIERWSZA PRÓBA					
DRUGA PRÓBA					

BEZ PUNKTÓW

UWAGA

Przeczytaj listę cyfr (w tempie 1 cyfra/sek.)

Badany/a powinien/powinna je powtórzyć w prawidłowej kolejności. [ ] 2 1 8 5 4
Badany/a powinien/powinna je powtórzyć w odwrotnej kolejności. [ ] 7 4 2

__/2

Przeczytaj ciąg liter. Badany/a powinien/powinna klasnąć za każdym razem, kiedy czytana jest litera A. Nie otrzymuje punktów, gdy popełni 2 lub więcej błędów.

[ ] F B A C M N A A J K L B A F A K D E A A A J A M O F A A B

__/1

Seria odejmowania po 7 zaczynając od 100. [ ] 93 [ ] 86 [ ] 79 [ ] 72 [ ] 65

4 lub 5 prawidłowych wyników: **3 pkt.**, 2 lub 3 wyniki prawidłowe: **2 pkt.**, 1 wynik prawidłowy: **1 pkt.**, 0 wyników prawidłowych: **0 pkt.**

__/3

JĘZYK

Powtórz zdania: Wiem tylko, że to Jan ma dzisiaj pomagać. [ ]
Kot zawsze chował się pod kanapą, gdy psy były w pokoju. [ ]

__/2

Fluencja / Wymień w ciągu minuty jak najwięcej słów, zaczynających się na literę F. [ ] ____ (N ≥ 11 słów)

__/1

ABSTRAHOWANIE

Proszę podać w czym są do siebie podobne: np. banan i pomarańcza = owoce [ ] pociąg – rower [ ] zegarek – linijka

__/2

PRZYPOMINANIE ODROCZONE

Punktacja Wskaźnika Pamięci (MIS)

(MIS)		TWARZ	AKSAMIT	KOŚCIÓŁ	STOKROTKA	CZERWONY	
X3	Odtwarzanie słów BEZ PODPOWIEDZI	[ ]	[ ]	[ ]	[ ]	[ ]	Punkty wyłącznie za odtwarzanie BEZ PODPOWIEDZI
X2	Kategoria						
X1	Wybór z listy						MIS = ____ / 15

__/5

ORIENTACJA

[ ] Data [ ] Miesiąc [ ] Rok [ ] Dzień [ ] Miejsce [ ] Miasto

__/6

© Z. Nasreddine MD www.mocatest.org

MIS: /15
(Norma ≥ 26/30)
Dodać 1 pkt., jeśli wykształcenie ≤ 12 lat

RAZEM __/30

Badanie przeprowadzone przez: ____________________

W celu zapewnienia rzetelności wymagane są szkolenie i certyfikacja

MOCA - Poland/Polish - Version of 19 Feb 2018 - Mapi.
ID061021 / MOCA-8.1-Test_AU1.0_pol-PL.doc

14.2.5 HADS ASSESSMENT FORM

The Polish version of the HADS Assessment Form.

HADS-M
Zmodyfikowana Skala HADS
Autorzy wersji oryginalnej: A.S Zigmond. R.P. Snaith
Opracowanie: M. Majkowicz, K de Walden-Gałuszko, G. Chojnacka-Szawłowska
Lekarze są przekonani, że emocje odgrywają rolę w wielu chorobach. Jeśli Pana (i) lekarz pozna lepiej Pana (i) uczucia będzie mógł lepiej Panu (i) pomóc. Kwestionariusz ten jest przeznaczony do pomocy lekarzowi celem poznania Pana (i) samopoczucia. Proszę przeczytać każde zdanie i zakreślić każdą kratkę z odpowiedzią, która jest najbliższa temu, jak się Pan (i) czuł (a) podczas ostatniego tygodnia. Proszę nie zastanawiać się zbyt długo nad odpowiedzią – Pana (i) natychmiastowa odpowiedź na każdy z punktów będzie prawdopodobnie bardziej trafna niż odpowiedź udzielona po dłuższym zastanowieniu.

1. Czułem (-am) się napięty (-a) lub podenerwowany (-a)

Większość czasu		[redacted]
Sporo czasu		[redacted]
Od czasu do czasu		[redacted]
Wcale		[redacted]

2. Wciąż cieszą mnie rzeczy, które zwykle sprawiały mi radość

Zdecydowanie tak samo	[redacted]	
Niezupełnie tak samo	[redacted]	
Tylko trochę	[redacted]	
Zupełnie nie	[redacted]	

3. Odczuwałem (-am) przerażające uczucie, jakby miało się zdarzyć coś okropnego

Tak, bardzo wyraźnie, coś bardzo złego		[redacted]
Wyraźnie, ale nie tak bardzo złego		[redacted]
Trochę, ale nie martwiło mnie to		[redacted]
Wcale czegoś takiego nie odczuwałem (-am)		[redacted]

4. Potrafię się śmiać i dostrzegać zabawną stronę zdarzeń

Tak samo jak kiedyś	[redacted]	
Teraz nie tak bardzo jak kiedyś	[redacted]	
Znacznie mniej niż kiedyś	[redacted]	
W ogóle nie	[redacted]	

5. Nachodzą mnie smutne myśli

Większą część czasu		[redacted]
Sporo czasu		[redacted]
Od czasu do czasu, ale niezbyt często		[redacted]
Przypadkowo, nieregularnie		[redacted]

6. Czuję się wesoły (-a) i pogodna (-a)

Wcale nie	[redacted]	
Nieczęsto	[redacted]	
Czasem	[redacted]	
Większość czasu	[redacted]	

7. Mogę siedzieć spokojnie i czuć się zrelaksowany (-a)

Zdecydowanie tak		[redacted]
Zwykle		[redacted]
Często		[redacted]
Wcale nie		[redacted]

8. Czuję się jakbym był (-a) „w psychicznym dołku"

Przez cały czas	[redacted]	
Bardzo często	[redacted]	
Od czasu do czasu	[redacted]	
Wcale nie	[redacted]	

9. Mam zatrważające uczucie, jakby mi się coś trzęsło w środku

Wcale nie		[redacted]
Od czasu do czasu		[redacted]
Dość często		[redacted]
Bardzo często		[redacted]

10. Przestałem (-am) interesować się swoim wyglądem zewnętrznym

Całkowicie przestałem(-am) się interesować	[redacted]	
Nie dbam o siebie tak jak powinien (powinnam)	[redacted]	
Nie jestem w stanie dbać o siebie	[redacted]	
Dbam o siebie tak jak zawsze	[redacted]	

11. Nie mogę spokojnie usiedzieć na miejscu

W bardzo znacznym stopniu		[redacted]
W znacznym stopniu		[redacted]
Rzadko		[redacted]
Mogę usiedzieć spokojnie		[redacted]

12. Oczekuję z radością na różne sprawy

Tak bardzo jak kiedyś	[redacted]	
Mniej niż kiedyś	[redacted]	
Zdecydowanie mniej niż zwykle	[redacted]	
Wcale nie	[redacted]	

13. Miewam nagle uczucie panicznego lęku

Bardzo często		[redacted]
Dość często		[redacted]
Niezbyt często		[redacted]
Wcale		[redacted]

14. Mogę się cieszyć dobrą książką, programem RTV

Często	[redacted]	
Czasami	[redacted]	
Niezbyt często	[redacted]	
Bardzo rzadko	[redacted]	

15. Zdarzało się, że w ciągu ostatniego tygodnia wybuchałem (-am) gniewem

Często		[redacted]
Czasami		[redacted]
Rzadko		[redacted]
Wcale		[redacted]

16. Zdarzało się, że denerwowałem (-am) się, „złościłem (-am) się” wewnętrznie

Często	[redacted]	
Czasami	[redacted]	
Rzadko	[redacted]	
Wcale	[redacted]	

14.2.6 IQCODE ASSESSMENT FORM

The Polish version of the IQCODE Assessment Form.

IQCODE

The Informant Questionnaire on Cognitive Decline in the Elderly.

Ankieta dotycząca sprawności funkcji poznawczych u osób w starszym wieku.

Proszę zastanowić się na tym jaka jest różnica pomiędzy obecnym zachowaniem Pana/i członka rodziny/przyjaciela obecnie w porównaniu do jego/jej zachowania się 10 lat temu. 10 lat temu był rok 20.....

Poniżej opisane są sytuacje, w których osoba której dotyczy ten formularz musi posłużyć się swą pamięcią, inteligencją. Proszę postarać się ocenić, czy przez te 10 lat doszło do pogorszenia lub może poprawy, czy też nie zaszły żadne zmiany w sposobie codziennego zachowania, funkcjonowania badanej osoby w poniżej podanych sytuacjach. Proszę zwrócić uwagę na **porównanie** sposobu zachowania osoby **obecnie i 10 lat temu**.

Jeżeli osoba, której dotyczy ten formularz 10 lat temu zapominała, gdzie odłożyła jakąś rzecz i obecnie także zapomina powinien Pan/i zakreślić odpowiedź:

Brak istotnej zmiany w zachowaniu.

Data : ....

Imię i nazwisko pacjenta: ....

Rodzaj pokrewieństwa / znajomości osoby wypełniającej ankietę

w stosunku do pacjenta: ....

Proszę zaznaczyć zaobserwowane zmiany przez zakreślenie kółkiem właściwej odpowiedzi.
W porównaniu do 10 lat wstecz osoba, której dotyczy ten formularz:

	1	2	3	4	5
1. Rozpoznaje twarze osób z rodziny i przyjaciół.	znaczna poprawa	lekka poprawa	brak istotnych zmian	lekkie pogorszenie	znaczne pogorszenie
2. Pamięta imiona osób z rodziny i przyjaciół.	znaczna poprawa	lekka poprawa	brak istotnych zmian	lekkie pogorszenie	znaczne pogorszenie
3. Pamięta rzeczy dotyczące rodziny i przyjaciół (np. zawód, dzień urodzin, adresy).	znaczna poprawa	lekka poprawa	brak istotnych zmian	lekkie pogorszenie	znaczne pogorszenie
4. Pamięta rzeczy, które miały niedawno miejsce.	znaczna poprawa	lekka poprawa	brak istotnych zmian	lekkie pogorszenie	znaczne pogorszenie
5. Potrafi odtworzyć rozmowę sprzed kilku dni.	znaczna poprawa	lekka poprawa	brak istotnych zmian	lekkie pogorszenie	znaczne pogorszenie
6. Zapomina w środku rozmowy co chciał/a powiedzieć.	znaczna poprawa	lekka poprawa	brak istotnych zmian	lekkie pogorszenie	znaczne pogorszenie
7. Pamięta swój adres i numer telefonu.	znaczna poprawa	lekka poprawa	brak istotnych zmian	lekkie pogorszenie	znaczne pogorszenie
8. Orientuje się jaki jest dzień tygodnia, miesiąc, rok.	znaczna poprawa	lekka poprawa	brak istotnych zmian	lekkie pogorszenie	znaczne pogorszenie
9. Pamięta, gdzie zazwyczaj trzymane są różne rzeczy.	znaczna poprawa	lekka poprawa	brak istotnych zmian	lekkie pogorszenie	znaczne pogorszenie
10.Umie przypomnieć sobie, gdzie można znaleźć rzeczy odłożone w inne niż zazwyczaj miejsce.	znaczna poprawa	lekka poprawa	brak istotnych zmian	lekkie pogorszenie	znaczne pogorszenie
11. Umie przystosować się do zmian w co- dziennym planie dnia.	znaczna poprawa	lekka poprawa	brak istotnych zmian	lekkie pogorszenie	znaczne pogorszenie
12.Umie posługiwać się zmechanizowanym sprzętem gospodarstwa domowego.	znaczna poprawa	lekka poprawa	brak istotnych zmian	lekkie pogorszenie	znaczne pogorszenie
13. Jest w stanie nauczyć się jak używać nowych przyrządów lub maszyn w gospodarstwie domowym.	znaczna poprawa	lekka poprawa	brak istotnych zmian	lekkie pogorszenie	znaczne pogorszenie

14. Posiada ogólną zdolność uczenia się różnych nowych rzeczy.	znaczna poprawa	lekka poprawa	brak istotnych zmian	lekkie pogorszenie	znaczne pogorszenie
15. Pamięta wydarzenia, które miały miejsce w jego / jej młodości.	znaczna poprawa	lekka poprawa	brak istotnych zmian	lekkie pogorszenie	znaczne pogorszenie
16. Pamięta rzeczy, których nauczył(a) się w młodości.	znaczna poprawa	lekka poprawa	brak istotnych zmian	lekkie pogorszenie	znaczne pogorszenie
17. Rozumie znaczenie rzadko używanych słów.	znaczna poprawa	lekka poprawa	brak istotnych zmian	lekkie pogorszenie	znaczne pogorszenie
18. Rozumie artykuły z czasopism lub gazet.	znaczna poprawa	lekka poprawa	brak istotnych zmian	lekkie pogorszenie	znaczne pogorszenie
19. Jest zdolny śledzić przebieg akcji w książkach lub serialach telewizyjnych.	znaczna poprawa	lekka poprawa	brak istotnych zmian	lekkie pogorszenie	znaczne pogorszenie
20. Daje sobie radę z napisaniem listu do przyjaciela lub w sprawach służbowych.	znaczna poprawa	lekka poprawa	brak istotnych zmian	lekkie pogorszenie	znaczne pogorszenie
21.Orientuje się w ważnych wydarzeniach historycznych.	znaczna poprawa	lekka poprawa	brak istotnych zmian	lekkie pogorszenie	znaczne pogorszenie
22. Podejmuje decyzje dotyczące codziennych spraw.	znaczna poprawa	lekka poprawa	brak istotnych zmian	lekkie pogorszenie	znaczne pogorszenie
23. Umie właściwie gospodarować pieniędzmi na zakupach.	znaczna poprawa	lekka poprawa	brak istotnych zmian	lekkie pogorszenie	znaczne pogorszenie
23. Potrafi zajmować się finansami (np. emerytura, renta, załatwienie spraw w banku, na poczcie).	znaczna poprawa	lekka poprawa	brak istotnych zmian	lekkie pogorszenie	znaczne pogorszenie
24. Daje sobie radę z innymi problemami życia codziennego (umie określić jak dużo kupić żywności, na kiedy planowane są odwiedziny przyjaciół).	znaczna poprawa	lekka poprawa	brak istotnych zmian	lekkie pogorszenie	znaczne pogorszenie

26.Umie zorientować się, co się wokół niego / niej dzieje, rozumie, dlaczego tak się dzieje.	znaczna poprawa	lekka poprawa	brak istotnych zmian	lekkie pogorszenie	znaczne pogorszenie

Komentarz:
IQCODE >=104 otępienie
Polska wersja:
Czułość 85%, swoistość 94%
Na podstawie pracy doktorskiej: Kraków 2005
Uniwersytet Jagielloński Collegium Medicum Wydział Lekarski
Aleksandra Klimkowicz-Mrowiec
„Czynniki ryzyka i częstość otępienia po udarze mózgu"
„Kwestionariusz dotyczący sprawności intelektualnej u osób starszych" to 26-punktowy, ustrukturyzowany wywiad, w którym oceniający – członek rodziny lub przyjaciel mający stały kontakt z ocenianym – ma za zadanie porównać obecną sprawność funkcji poznawczych badanego ze stanem sprzed 10 lat (okres 10 lat został wybrany jako punkt odcięcia, ponieważ dane epidemiologiczne wykazują, że czas od momentu ujawnienia się otępienia do śmierci jest zwykle krótszy) [79]. Na każde pytanie kwestionariusza można odpowiedzieć, wybierając spośród pięciu identycznych dla każdego pytania odpowiedzi o wartości punktowej od 1 do 5. Wynik, jaki może uzyskać oceniany, mieści się w przedziale od 26 do 130 punktów.
Przeprowadzone do tej pory badania z użyciem IQCODE w różnych wersjach językowych, dotyczące diagnozy otępienia o różnej etiologii, wykazały przydatność tego narzędzia zarówno do oceny sprawności poznawczej w ogólnej populacji, jak i w badaniach klinicznych dotyczących różnych jednostek chorobowych. Istnieją również dowody potwierdzające skuteczność IQCODE jako metody przesiewowej w kierunku oceny otępienia niezależnie od jego etiologii i stopnia nasilenia. Czułość i swoistość kwastionariusza są różne w zależności od przyjętego punktu odcięcia. Najlepszą równowagę uzyskuje się dla wyniku IQCODE □ 104 punktów. Czułość i swoistość dla tego punktu odcięcia wynoszą odpowiednio 88% i 92,7% [78]. Najczęściej używanym narzędziem w diagnostyce przesiewowej w kierunku otępienia jest Krótka Skala Oceny Funkcji Poznawczych (MMSE) [50]. Jako punkt odcięcia dla diagnozy otępienia za pomocą tej skali przyjmuje się przeważnie 23 punkty na 30 możliwych do uzyskania. Czułość i swoistość MMSE dla wyniku 23 punkty ocenia się odpowiednio na 75% i 82,3% [85]. Na wynik uzyskany w MMSE przez badanego wpływa poziom wykształcenia, wiek oraz ogólna sprawność fizyczna, natomiast na wynik uzyskany w IQCODE nie wpływa wcześniejszy poziom inteligencji, ogólna stopień wykształcenia, sprawność fizyczna, wiek czy pozycja socjalno-ekonomiczna [78, 81, 109], gdyż IQCODE mierzy nie sam poziom sprawności poznawczej, ale jego ewentualną zmianę [79].
Opisano dobrą korelację wyników w IQCODE z wynikami baterii testów neuropsychologicznych wynoszącą od 0,2 do 0,4 w zależności od zastosowanego testu. Najwyższą korelację zanotowano dla odtwarzania historii (r=0,42), rozpoznawania słów (r=0,44) i czasu reakcji (r=0,40) [77]. Wynik IQCODE wysoko koreluje z wynikiem MMSE (r=0,74) [65]. Korelacja pomiędzy wynikiem IQCOCE a depresją i lękiem jest niska i wynosi odpowiednio r=0,14 i r=0,10 [77].
Kwestionariusz ten jest narzędziem użytecznym w różnych kulturach i w różnych wersjach językowych. Na potrzeby obecnego badania po uzyskaniu zgody autora kwestionariusza tłumacz dokonał przekładu pytań z języka angielskiego na język polski. Następnie kolejny tłumacz dokonał przekładu z języka polskiego na język angielski. Obydwie wersje kwestionariusza w języku angielskim zostały porównane; uzyskano wysoką zgodność tłumaczeń. Polska wersja kwestionariusza z2ostała następnie zamieszczona na stronie internetowej utworzonej przez autora IQCODE (**http://www.anu.edu.au/iqcode/**).

Największą zaletą IQCODE w aspekcie otępienia przedudarowego, które ocenia się w ostrej fazy udaru, jest możliwość wypełnienia go bez udziału chorego w okresie, kiedy dokładna diagnoza neuropsychologiczna jest najczęściej niemożliwa. Czas potrzebny do wypełnienia kwestionariusza to 5 - 10 minut. Warunkiem zastosowania tej metody jest obecność wiarygodnego, dobrze znającego chorego informatora. W badanym materiale tylko niecały 1% chorych z udarem przyjętych do szpitala w okresie rekrutacji nie miał wiarygodnego informatora.
